# Axicabtagene ciloleucel as second-line therapy in large B cell lymphoma ineligible for autologous stem cell transplantation: a phase 2 trial

**DOI:** 10.1038/s41591-023-02572-5

**Published:** 2023-09-14

**Authors:** Roch Houot, Emmanuel Bachy, Guillaume Cartron, François-Xavier Gros, Franck Morschhauser, Lucie Oberic, Thomas Gastinne, Pierre Feugier, Rémy Duléry, Catherine Thieblemont, Magalie Joris, Fabrice Jardin, Sylvain Choquet, Olivier Casasnovas, Gabriel Brisou, Morgane Cheminant, Jacques-Olivier Bay, Francisco Llamas Gutierrez, Cédric Menard, Karin Tarte, Marie-Hélène Delfau, Cédric Portugues, Emmanuel Itti, Xavier Palard-Novello, Paul Blanc-Durand, Yassine Al Tabaa, Clément Bailly, Camille Laurent, François Lemonnier

**Affiliations:** 1 Department of Hematology, University Hospital of Rennes, UMR U1236, INSERM, University of Rennes, French Blood Establishment, Rennes, France; 2 Department of Hematology, Lyon Sud Hospital Center, INSERM U1111, Lyon, France; 3 Department of Hematology, University Hospital of Montpellier, UMR-CNRS 5535, Montpellier, France; 4 Department of Clinical Hematology and Cellular Therapy, University Hospital of Bordeaux, Bordeaux, France; 5 ULR 7365 - GRITA, University Hospital of Lille, Lille, France; 6 Department of Hematology, Cancer University Institute of Toulouse Oncopole, Toulouse, France; 7 Department of Hematology, University Hospital of Nantes, Nantes, France; 8 Department of Hematology, University Hospital of Nancy, INSERM 1256, University of Lorraine, Vandœuvre-lès-Nancy, France; 9 Department of Clinical Hematology and Cellular Therapy, Sorbonne University, Saint-Antoine Hospital, AP-HP, INSERM UMR938, Paris, France; 10 Department of Hematology and Oncology, Saint-Louis Hospital, AP-HP, Paris, France; 11 Department of Hematology, University Hospital of Amiens, Amiens, France; 12 Department of Clinical Hematology, Henri Becquerel Center, INSERM U1245, Rouen, France; 13 Department of Hematology, University Hospital Pitié Salpêtrière, AP-HP, Sorbonne University, Paris, France; 14 Department of Clinical Hematology, Dijon University Hospital, INSERM UMR1231, Dijon, France; 15 Department of Hematology, Institut Paoli-Calmettes, Marseille, France; 16 Department of Clinical Hematology, Necker-Enfants Malades University Hospital, AP-HP, INSERM UMR1163, Paris, France; 17 Department of Clinical Hematology and Cellular Therapy, Clermont-Ferrand University Hospital Center, Clermont-Ferrand, France; 18 Department of Anatomopathology, University Hospital of Rennes, Rennes, France; 19 French Blood Establishment and SITI Laboratory, UMR U1236, INSERM, University of Rennes, University Hospital Center of Rennes, Rennes, France; 20 Department of Immunology, Henri Mondor Hospital, Créteil, France; 21 Department of Biostatistics, LYSARC, Lyon-Sud Hospital, Pierre-Bénite, France; 22 Department of Nuclear Medicine, Henri Mondor Hospital, Créteil, France; 23 Department of Nuclear Medicine, University of Rennes, CLCC Eugène Marquis, INSERM, Rennes, France; 24 Department of Nuclear Medicine, CHU H. Mondor, U-PEC, AP-HP, Créteil, France; 25 Scintidoc Nuclear Medicine Center, Clinique Clémentville, Montpellier, France; 26 Nantes-Angers Cancer Research Center CRCI2NA, University of Nantes, INSERM UMR1307, CNRS-ERL6075, Nantes, France; 27 Department of Pathology, Cancer University Institute of Toulouse Oncopole, CHU Toulouse, CRCT INSERM U1037, Toulouse, France; 28 Lymphoid Malignancies Unit, Henri Mondor Hospital, Mondor Institute for Biomedical Research, INSERM U955, University Paris-Est, Créteil, France

**Keywords:** Phase II trials, B-cell lymphoma, Cancer immunotherapy

## Abstract

Axicabtagene ciloleucel (axi-cel) demonstrated superior efficacy compared to standard of care as second-line therapy in patients with high-risk relapsed/refractory (R/R) large B cell lymphoma (LBCL) considered eligible for autologous stem cell transplantation (ASCT); however, in clinical practice, roughly half of patients with R/R LBCL are deemed unsuitable candidates for ASCT. The efficacy of axi-cel remains to be ascertained in transplant-ineligible patients. ALYCANTE, an open-label, phase 2 study, evaluated axi-cel as a second-line therapy in 62 patients with R/R LBCL who were considered ineligible for ASCT. The primary end point was investigator-assessed complete metabolic response at 3 months from the axi-cel infusion. Key secondary end points included progression-free survival, overall survival and safety. The study met its primary end point with a complete metabolic response of 71.0% (95% confidence interval, 58.1–81.8%) at 3 months. With a median follow-up of 12.0 months (range, 2.1–17.9), median progression-free survival was 11.8 months (95% confidence interval, 8.4–not reached) and overall survival was not reached. There was no unexpected toxicity. Grade 3–4 cytokine release syndrome and neurologic events occurred in 8.1% and 14.5% of patients, respectively. These results support axi-cel as second-line therapy in patients with R/R LBCL ineligible for ASCT. ClinicalTrials.gov Identifier: NCT04531046.

## Abstract (web summary)

In the ALYCANTE trial, second-line treatment with axicabtagene ciloleucel, a CD19-targeting CAR T-cell therapy, resulted in high response rates and durable remissions in patients with high-risk large B-cell lymphoma deemed ineligible for autologous stem cell transplantation.

## Main

Large B cell lymphoma (LBCL) is successfully treated in approximately two-thirds of patients with rituximab-based chemoimmunotherapy^1,2^. Until recently, the standard of care (SOC) for second-line therapy consisted of salvage chemoimmunotherapy followed, if possible, by consolidation with high-dose chemotherapy (HDCT) and autologous stem cell transplantation (ASCT) in fit and responding patients^3^. However, patients who are primary refractory or who relapse early after a rituximab-containing first-line therapy, notably within a year from initial therapy, have a poor prognosis with standard salvage chemoimmunotherapy^4–6^. The recent advent of chimeric antigen receptor (CAR)-T cell therapy has led to an important paradigm shift in the management of these patients with high-risk relapsed/refractory (R/R) LBCL^7–10^.

In patients with high-risk R/R LBCL considered eligible for ASCT, axicabtagene ciloleucel (axi-cel), an autologous anti-CD19 CAR-T cell therapy, demonstrated superior efficacy over the SOC as a second-line therapy in the ZUMA-7 trial^8^. In this international, phase 3 trial, patients intended for transplant were randomized to receive either a single infusion of axi-cel after fludarabine and cyclophosphamide lymphodepleting chemotherapy or SOC consisting of two or three cycles of chemoimmunotherapy followed by HDCT/ASCT in patients who achieved a complete or partial remission. At a median follow-up of 24.9 months, axi-cel demonstrated superior efficacy compared to SOC, with an estimated median event-free survival (EFS) of 8.3 versus 2.0 months, respectively (*P* < 0.001)^8^. Moreover, at a median follow-up of 47.2 months, overall survival (OS) was superior in the axi-cel arm compared to the SOC (median not reached versus 31.1 months, respectively; *P* = 0.03)^11^.

In clinical practice, about half of patients with R/R LBCL are considered ineligible for HDCT/ASCT^3^. This population has not been evaluated in the ZUMA-7 trial^8^. Several factors may preclude patients from receiving HDCT/ASCT including advanced age, frailty and coexisting medical conditions^12^. Furthermore, patients who have undergone a previous ASCT as first-line consolidation are usually considered ineligible for a second ASCT^12^.

The prognosis of patients with R/R LBCL who are ineligible for HDCT/ASCT is usually poor with standard salvage chemoimmunotherapy^6,13^. Typical therapeutic choices within this context encompass rituximab, gemcitabine and oxaliplatin (R-GemOx)^14^; polatuzumab vedotin plus bendamustine and rituximab (Pola-BR)^15^; and tafasitamab plus lenalidomide (Tafa-Len)^16^. The R-GemOx regimen is one of the most widely used because of its acceptable tolerability. The real-world use of R-GemOx was evaluated in a large retrospective analysis of 196 patients with R/R LBCL who were not eligible for HDCT/ASCT^6^. At a median follow-up of 22 months, R-GemOx demonstrated modest efficacy, with a complete response rate of 33% and median progression-free survival (PFS) and OS of 5 and 10 months, respectively^6^. Patients who were refractory or who relapsed within 1 year after first-line chemoimmunotherapy with rituximab, cyclophosphamide, doxorubicin, vincristine and prednisone (R-CHOP) (*n* = 60) had a particularly poor prognosis when treated with second-line R-GemOx, with a complete response rate of 12% and a PFS of 22% at 6 months and 14% at 1 year^17^. Thus, in patients with R/R LBCL who are not eligible for HDCT/ASCT, the outcome is poor after second-line chemoimmunotherapy, notably in patients who are refractory or relapse early (within 12 months from first-line therapy).

Although axi-cel has not been evaluated as a second-line therapy in patients with R/R LBCL who are ineligible for HDCT/ASCT, clinical trial data and real-world evidence have shown that CAR-T cell therapy is feasible in a subset of transplant-ineligible patients, notably in elderly and less-fit patients^12,18–23^. In this context, we conducted a phase 2 study (ALYCANTE) to assess the efficacy and safety of a single axi-cel infusion as a second-line therapy in patients with high-risk R/R LBCL deemed ineligible for ASCT but eligible for CAR-T cell therapy.

## Results

The primary end point was investigator-assessed complete metabolic response (CMR) at 3 months from the axi-cel infusion. Secondary end points were objective response rate (ORR) at 3 months from the axi-cel infusion, CMR at 6 months from the axi-cel infusion, best ORR, best CMR, duration of response (DOR), EFS from leukapheresis, PFS from infusion, OS from infusion and the incidence, nature and severity of adverse events. Additional planned secondary end points not reported in this manuscript include health-related quality of life and the cell product characteristics and cellular kinetics of axi-cel.

In the initial study protocol, we calculated that a sample size of 40 patients with aggressive B cell non-Hodgkin lymphoma that was refractory to or had relapsed no more than 12 months after first-line chemoimmunotherapy and who were ineligible for HDCT/ASCT based on a physician’s assessment was sufficient to test the efficacy of axi-cel infusion. Our initial part of the study (*n* = 40) met its primary end point with an investigator-assessed CMR of 67.5% at 3 months after infusion. The protocol was amended to enroll additional patients to enable a balanced comparison of the efficacy and toxicity of axi-cel in different age subgroups (<70 and ≥70 years). Here, we report the results of the study cohort of 62 patients who received axi-cel infusion. Of note, patient characteristics and outcomes were similar between the initial (*n* = 40) and expanded (*n* = 62) cohorts (Extended Data Table 1).

### Patients and treatment

**Fig. 1:**
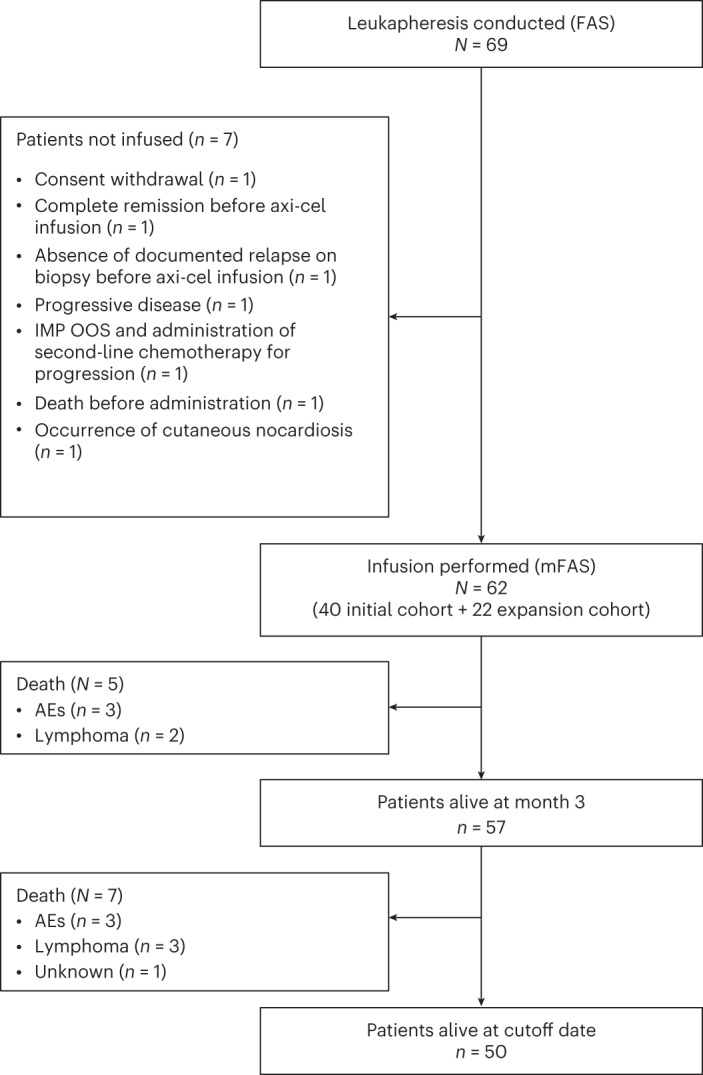
Patient disposition at the data cutoff date of 19 January 2023. IMP OOS, investigational medicinal product out of specification.

**Extended Data Fig. 1:**
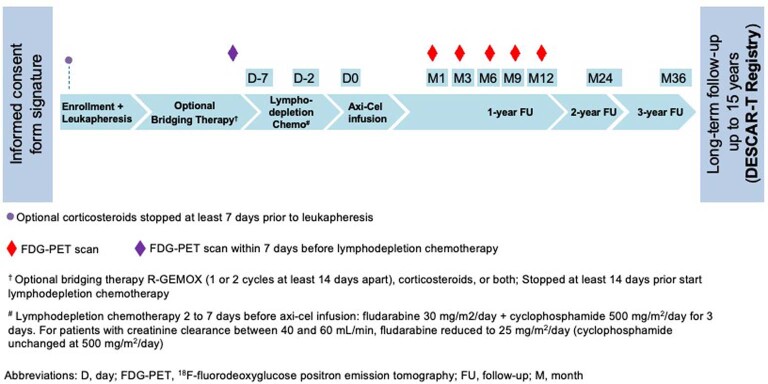
Study design and overview of study procedures.

Between 19 March 2021 and 4 May 2022, a total of 69 patients were enrolled and underwent leukapheresis, representing the full analysis set (FAS). Of these 69 patients, 62 (89.8%) received a single axi-cel infusion between 26 April 2021 and 16 June 2022 and were consequently included in the modified full analysis set (mFAS). There were five important protocol deviations from inclusion criteria: three related to a relapsed disease occurring beyond 12 months from completion of first-line chemoimmunotherapy and two related to patients with a histological diagnosis of grade 1–3A follicular lymphoma after central review. The patient disposition flow diagram is shown in Fig. 1. The study design is summarized in Extended Data Fig. 1. Overall, seven patients underwent leukapheresis but did not receive axi-cel infusion because of disease progression (*n* = 1), investigational medicinal product out of specification (*n* = 1), CMR before axi-cel infusion (*n* = 1) based on the investigator’s assessment, absence of documented relapse on biopsy before axi-cel infusion (*n* = 1), consent withdrawal (*n* = 1), occurrence of cutaneous nocardiosis (*n* = 1) and lymphoma-related death (*n* = 1) (Fig. 1). At the data cutoff date of 19 January 2023, median time between study inclusion and axi-cel infusion was 41.5 days (interquartile range (IQR), 38.0–48.0) and median follow-up duration from axi-cel infusion was 12.0 months (IQR, 9.1–12.6).

**Extended Data Fig. 2:**
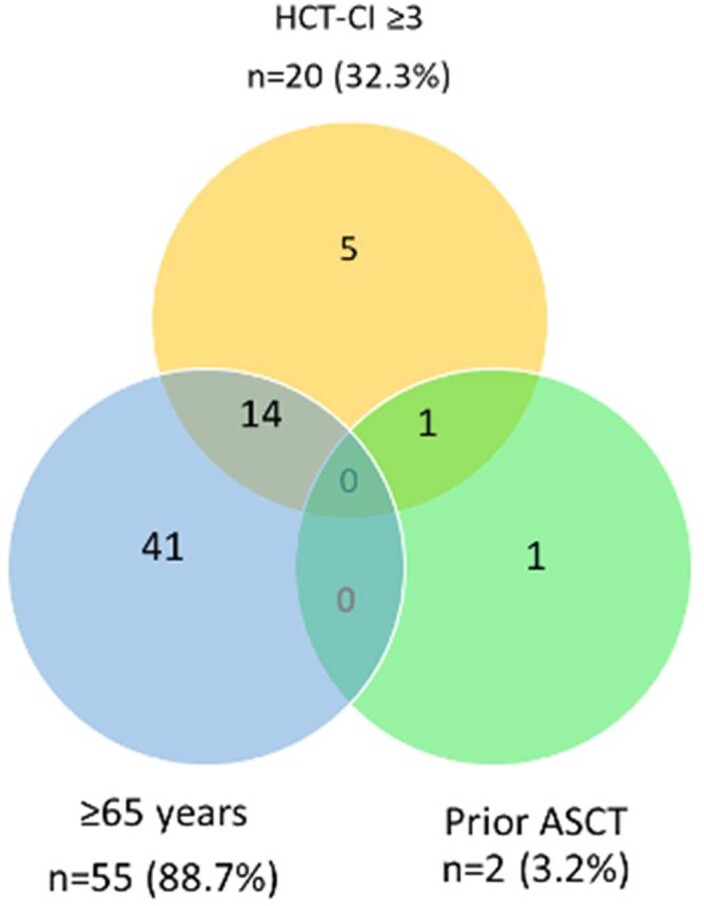
Causes of autologous stem cell transplantation (ASCT)-ineligibility in the modified full analysis set (N = 62). Abbreviation: HCT-CI, hematopoietic cell transplantation-specific comorbidity index.

The demographics and disease characteristics of patients in the mFAS (*N* = 62) are summarized in Table 1. Median age was 70 years (range, 49–81), 15 patients (24.2%) were female and 35 patients (56.5%) had an international prognostic index (IPI) of 3 or higher. Almost all patients had an Eastern Cooperative Oncology Group (ECOG) performance status of 0 or 1 (*n* = 61; 98.4%). The majority of patients (*n* = 52; 83.9%) were histologically diagnosed with diffuse large B cell lymphoma (DLBCL). In total, 34 patients (54.8%) had primary refractory disease to first-line chemoimmunotherapy. Patients were deemed ineligible for HDCT/ASCT because of age ≥65 years (88.7%), high hematopoietic cell transplantation-specific comorbidity index (HCT-CI) score ≥3 (32.3%)^24^ and/or previous ASCT (3.2%) (Extended Data Fig. 2). Overall, 52 patients (83.9%) received bridging therapy after leukapheresis at the investigator’s discretion. In 51 of these 52 patients (98.1%), bridging therapy consisted of R-GemOx administered for one cycle (*n* = 25), two cycles (*n* = 25) or three cycles (*n* = 1). Additionally, 9 out of 52 patients (17.3%) received corticosteroids, of whom one patient received corticosteroids alone without R-GemOx. Among patients who received bridging therapy, 63.4% did not respond (stable disease or progressive disease).

**Table 1:** Baseline characteristics of the modified full analysis set (*N* = 62)

	Patients who received axi-cel (*N* = 62)
**Median age (minimum–maximum), years**	70.0 (49.0–81.0)
**Age groups,** ***n*** **(%)**	
≥65 years	55 (88.7)
≥70 years	33 (53.2)
≥75 years	7 (11.3)
**Sex,** ***n*** **(%)**	
Female	15 (24.2)
Male	47 (75.8)
**ECOG performance status at screening,** ***n*** **(%)**	
0–1	61 (98.4)
2	1 (1.6)
**Ann Arbor stage at screening,** ***n*** **(%)**	
I–II	16 (25.8)
III–IV	46 (74.2)
**Presence of** ≥**2 extranodal sites at screening,** ***n*** **(%)**	20 (32.3)
**IPI at screening,** ***n*** **(%)**	
0–1	7 (11.3)
2	20 (32.3)
3	23 (37.1)
4	12 (19.4)
5	0 (0.0)
**HCT-CI score,** ***n*** **(%)**	
<3	42 (67.7)
≥3	20 (32.3)
**TMTV,** ***n*** **(%)**	
≤80 ml	38 (61.3)
>80 ml	22 (35.5)
Missing data	2 (3.2)
**Median TMTV (minimum–maximum), ml**	44.0 (0–829)
**Histological subtype at screening by central review,** ***n*** **(%)**	
DLBCL, not otherwise specified	52 (83.9)
HGBCL with *MYC*-R and *BCL2*-R +/− *BCL6*-R	6 (9.7)
Follicular lymphoma with possible transformation into DLBCL, not otherwise specified	1 (1.6)
Follicular lymphoma, grade 1, 2 and 3A	2 (3.2)
Follicular lymphoma, grade 3B	1 (1.6)
**Refractory status to first-line chemoimmunotherapy,** ***n*** **(%)**	34 (54.8)
**First-line therapy,** ***n*** **(%)**	
** Chemoimmunotherapy**	62 (100)
R-CHOP^a^	36 (58.1)
R-CHOP + X^a^	25 (40.3)
R-COPADEM-based treatment	1 (1.6)
** ASCT**	2 (3.2)
**Best response to first-line therapy,** ***n*** **(%)**	
Complete response	30 (48.4)
Partial response	16 (25.8)
Stable disease	1 (1.6)
Progressive disease	14 (22.6)
Not evaluated^b^	1 (1.6)
**Bridging therapy between study enrollment and axi-cel infusion,** ***n*** **(%)**	52 (83.9)
R-GemOx	51/52 (98.1)
Corticosteroids^c^	9/52 (17.3)
** Response to bridging therapy (*N*** = **52),** ***n*** **(%)**	
Complete response	4/52 (7.7)
Partial response	14/52 (26.9)
Stable disease	14/52 (26.9)
Progressive disease	19/52 (36.5)
Not evaluated	1/52 (1.9)
**Metabolic disease status before axi-cel infusion (*N*** = **62),** ***n*** **(%)**	
Complete response	4 (6.5)
Partial response	15 (24.2)
Stable disease	18 (29.0)
Progressive disease	23 (37.1)
Not evaluated	2 (3.2)
**Elevated lactate dehydrogenase level at axi-cel infusion,** ***n*** **(%)**	7 (11.3)
**C-reactive protein level** **>** **30** **mg** **l**^**−1**^ **at axi-cel infusion,** ***n*** **(%)**	12 (19.4)
**Median time between end of first-line therapy and refractory disease/relapse (minimum–maximum), months**	2.1 (0.0–17.0)
**Median time between end of first-line therapy and relapse (minimum–maximum), months**	5.9 (1.0–17.0)
**Median time between study inclusion and axi-cel infusion (minimum–maximum), days**	41.5 (21.0-71.0)

^a^Three patients (one treated with R-CHOP and two treated with R-CHOP + methotrexate) received radiotherapy following chemoimmunotherapy.

^b^Enrolled in the study because of refractory disease.

^c^One patient received corticosteroids only.

ASCT, autologous stem cell transplantation; DLBCL, diffuse large B cell lymphoma; ECOG, Eastern Cooperative Oncology Group; HCT-CI, hematopoietic cell transplantation-specific comorbidity index; HGBCL with *MYC*-R and *BCL2*-R ± *BCL6*-R, high-grade B cell lymphoma with *MYC* and *BCL2* rearrangements associated or not with *BCL6* rearrangement; IPI, international prognostic index; R-CHOP, rituximab plus cyclophosphamide, doxorubicin, vincristine and prednisone; R-CHOP + X, addition of methotrexate (*n* = 22), etoposide (*n* = 1) or another investigational chemotherapy drug (*n* = 2) to R-CHOP; R-COPADEM, rituximab, cyclophosphamide, vincristine, prednisone, doxorubicin and methotrexate; R-GemOx, rituximab, gemcitabine and oxaliplatin; TMTV, total metabolic tumor volume.

### Primary efficacy outcome

**Extended Data Fig. 3:**
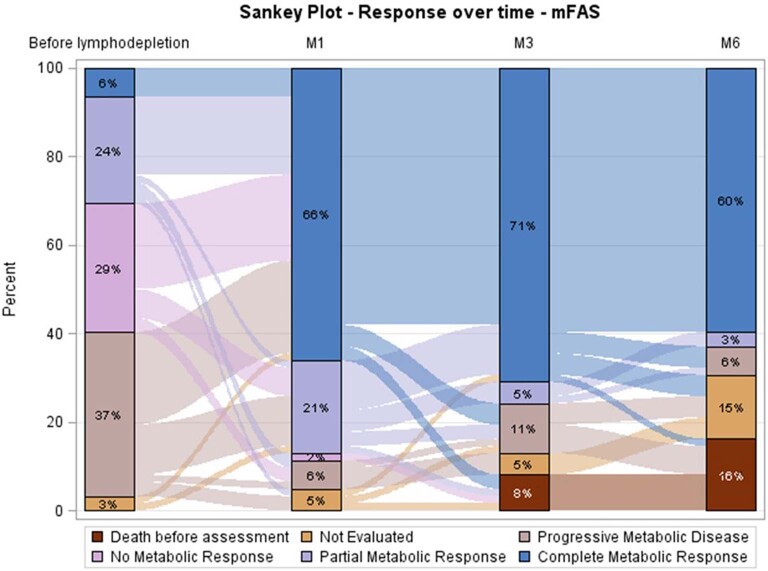
Metabolic response over time in the modified full analysis set (mFAS) (N = 62).

The CMR at 3 months from the axi-cel infusion, as assessed by the investigator according to the Lugano response criteria^25^, was 71.0% (95% confidence interval (CI), 58.1–81.8%) in the mFAS (*N* = 62) (Table 2). Compared to the mFAS, the sensitivity analysis on the FAS (*N* = 69) did not show a notable difference in the investigator-assessed CMR at 3 months, which was 66.7% (95% CI, 54.3–77.6%). Likewise, a post-hoc analysis, which excluded two patients with grade 1–3A follicular lymphoma and four patients who achieved complete response with bridging therapy, found an investigator-assessed CMR at 3 months of 67.9% (95% CI, 54.0–79.7%), which is comparable to the 3-month CMR reported in the mFAS. Of note, out of ten patients with a partial metabolic response (PMR) at 1 month after axi-cel infusion, five patients converted to a CMR at 3 months without any additional therapy (Extended Data Fig. 3).

**Table 2:** Metabolic response in the modified full analysis set (*N* = 62) according to the Lugano response criteria^25^

Response	Investigator-assessed (%)	Assessed by a central review panel (%)
**Response at 3 months**		
Objective response	47 (75.8)	43 (69.4)
Complete response	44 (71.0)	41 (66.1)
Partial response	3 (4.8)	2 (3.2)
Stable disease	0	1 (1.6)
Progressive disease	7 (11.3)	9 (14.5)
Not evaluated	8^a^ (12.9)	9 (14.5)
**Best response**		
Objective response	56 (90.3)	57 (91.9)
Complete response	49 (79.0)	51 (82.3)
Partial response	7 (11.3)	6 (9.7)
Stable disease	3 (4.8)	1 (1.6)
Progressive disease	3 (4.8)	4 (6.5)
Not evaluated	0	0

All data are expressed as *n* (%).

^a^Five patients died before reaching the evaluation at 3 months (three due to adverse effects and two due to lymphoma) and three patients relapsed before reaching the evaluation at 3 months.

### Secondary efficacy outcomes

At 3 months from the axi-cel infusion, the investigator-assessed ORR was 75.8% (95% CI, 63.3–85.8%). In total, 37 patients (59.7%) remained in CMR, as assessed by the investigator, at 6 months from the axi-cel infusion (95% CI, 46.5–72.0%) (Extended Data Fig. 3). The investigator-assessed best ORR and best CMR were 90.3% and 79.0%, respectively. When assessed by a central review panel, CMR and ORR at 3 months were 66.1% (95% CI, 53.0–77.7%) and 69.4% (95% CI, 56.4–80.4%), respectively. The best ORR and best CMR as assessed by the central review panel were 91.9% and 82.3%, respectively (Table 2).

**Fig. 2:**
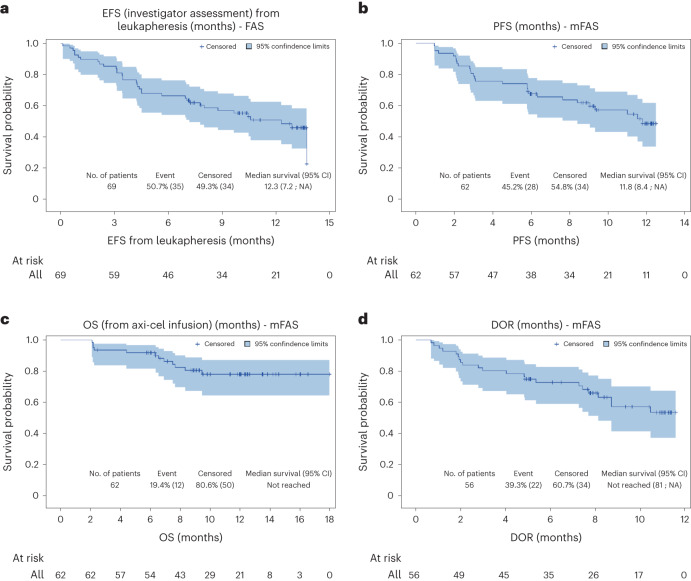
Kaplan–Meier estimates of event-free survival from leukapheresis, progression-free survival from axi-cel infusion, overall survival from axi-cel infusion and duration of response. **a**–**d**, Kaplan–Meier estimates of EFS from leukapheresis (**a**), PFS from axi-cel infusion (**b**), OS from axi-cel infusion (**c**) and DOR (**d**). The blue-shaded areas around the survival curve represent the Hall–Wellner 95% confidence bands. NA, not attained.

At a median follow-up of 12.0 months, the median EFS from leukapheresis was 12.3 months (95% CI, 7.2–not reached; Fig. 2a) in the FAS (*n* = 69). The estimated EFS rates at 6 and 12 months were 66.7% (95% CI, 54.2–76.4%) and 51.2% (95% CI, 38.2–62.8%), respectively. Median PFS from axi-cel infusion was 11.8 months (95% CI, 8.4–not reached; Fig. 2b) in the mFAS (*N* = 62). The estimated PFS rates at 6 and 12 months were 67.7% (95% CI, 54.5–77.8%) and 48.8% (95% CI, 34.0–62.0%), respectively. Median OS from axi-cel infusion was not reached (Fig. 2c) in the mFAS (*N* = 62). The estimated OS rates at 6 and 12 months were 91.9% (95% CI, 81.6–96.5%) and 78.3% (95% CI, 64.7–87.1%), respectively. Median DOR was not reached (Fig. 2d).

### Safety

All study participants had at least one adverse effect of any grade. Adverse effects of grade 3 or higher occurred in 59 out of 62 patients (95.2%). The most commonly reported adverse effects of grade 3 or higher were neutropenia (occurring in 66.1% of patients), anemia (38.7%) and thrombocytopenia (38.7%) (Extended Data Table 2). Various infections of any grade (that is, COVID-19, urinary tract infection, sepsis, respiratory tract infection, skin infection) occurred in 33 patients (53.2%). The most frequent infection was COVID-19, which was reported in 15 patients (24.2%), including 7 with grade ≥3 and 2 with grade 5.

Adverse effects of special interest related to CAR-T cell toxicities are reported in Table 3. Cytokine release syndrome (CRS) occurred in 93.5% of patients, with CRS of grade 3 or higher reported in 8.1% of patients. The median time to onset of CRS was 1.5 days (IQR, 1.0–3.0) after axi-cel infusion and the median CRS duration was 5.0 days (IQR, 4.0–9.0). Immune effector cell-associated neurotoxicity syndrome (ICANS) occurred in 51.6% of patients, with ICANS of grade 3 or higher reported in 14.5% of patients. The median time to onset of ICANS was 6.0 days (IQR, 5.0–8.0) after axi-cel infusion and the median ICANS duration was 5.0 days (IQR, 3.0–8.0). No deaths related to CRS or neurologic events occurred. CAR-T cell toxicities were mainly managed with the interleukin-6 receptor antagonist tocilizumab (administered in 77.4% of patients) and/or corticosteroids (64.5% of patients). A total of 16 patients (25.8%) were admitted to the intensive care unit (ICU) as a result of CAR-T cell toxicities. Grade 3 or worse prolonged cytopenias (defined as a grade ≥3 laboratory result of anemia, neutropenia and/or thrombocytopenia not resolved 30 days after axi-cel infusion) occurred in 23 out of 62 patients (37.1%).

**Table 3:** Adverse events of special interest in the modified full analysis set (*N* = 62)

	Patients who received axi-cel (*N* = 62)
**CRS,** ***n*** **(%)**	
Any	58 (93.5)
Grade 1–2	53 (85.5)
Grade 3–4	5 (8.1)
Median time to onset (Q1–Q3), days	1.5 (1.0–3.0)
Median duration (Q1–Q3), days	5.0 (4.0–9.0)
**ICANS,** ***n*** **(%)**	
Any	32 (51.6)
Grade 1–2	23 (37.1)
Grade 3–4	9 (14.5)
Median time to onset (Q1–Q3), days	6.0 (5.0–8.0)
Median duration (Q1–Q3), days	5.0 (3.0–8.0)
**Grade** ≥ **3 prolonged cytopenia**^a^, ***n*** **(%)**	23 (37.1)
Grade ≥3 prolonged neutropenia	15 (24.2)
Grade ≥3 prolonged anemia	14 (22.6)
Grade ≥3 prolonged thrombocytopenia	14 (22.6)
**Use of tocilizumab to manage CAR-T cell toxicities,** ***n*** **(%)**	48 (77.4)
**Use of corticosteroids to manage CAR-T cell toxicities,** ***n*** **(%)**	40 (64.5)
**ICU transfer due to CAR-T cell toxicities,** ***n*** **(%)**	16 (25.8)
**Infections,** ***n*** **(%)**	
Any	33 (53.2)
Grade 3–4	11 (17.7)
Grade 5	6 (9.7)
**Nonrelapse mortality,** ***n*** **(%)**	6 (9.7)

^a^Grade ≥3 prolonged cytopenia was defined as a grade ≥3 laboratory result of anemia, neutropenia and/or thrombocytopenia not resolved 30 days after axi-cel infusion. CAR, chimeric antigen receptor; CRS, cytokine release syndrome; ICANS, immune effector cell-associated neurotoxicity syndrome; ICU, intensive care unit; Q, quartile.

At the time of data cutoff, 12 patients died, 5 of whom from lymphoma and 1 of unknown reason. Nonrelapse mortality (NRM) was recorded in six patients (9.7%). All fatal adverse effects occurred late (beyond 2 months after axi-cel infusion) and were infections: two COVID-19, one aspergillosis, one mucormycosis, one sepsis and one perineal infection (Extended Data Table 3).

### Subgroup analysis

**Fig. 3:**
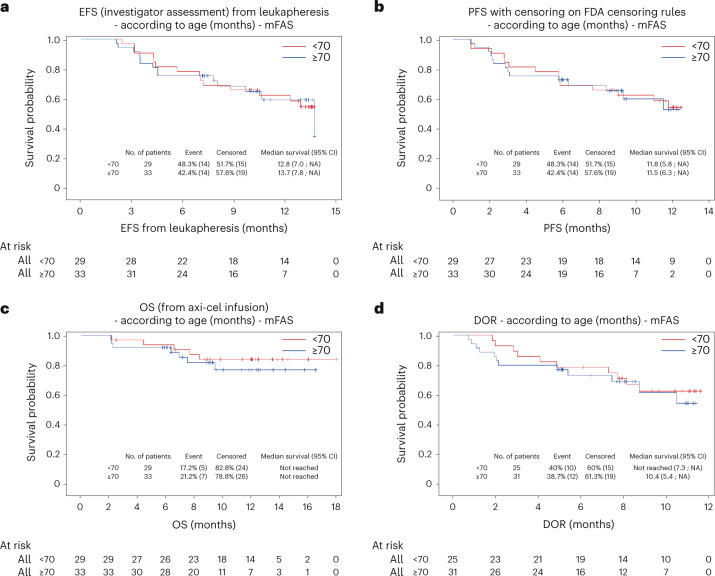
Kaplan–Meier estimates according to age of event-free survival from leukapheresis, progression-free survival from axi-cel infusion, overall survival from axi-cel infusion and duration of response. **a**–**d**, Kaplan–Meier estimates according to age (<70 versus ≥70 years) of EFS from leukapheresis (**a**), PFS from axi-cel infusion (**b**), OS from axi-cel infusion (**c**) and DOR (**d**).

**Extended Data Fig. 4:**
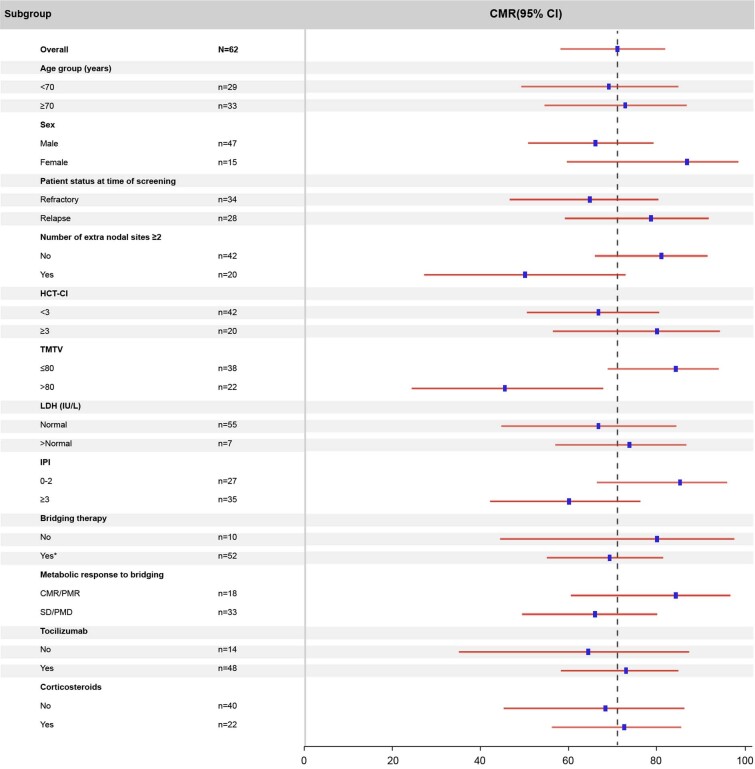
Forest plot illustrating complete metabolic response (CMR) rates at 3 months by subgroup. Blue squares denote CMR rates, and error bars indicate 95% two-sided 95% exact Clopper-Pearson confidence intervals (CIs). The overall study population is the modified full analysis set (N = 62), in which CMR at 3 months from the axi-cel infusion was 71.0% (95% CI, 58.1%–81.8%). Abbreviations: HCT-CI, hematopoietic cell transplantation-specific comorbidity index; IPI, international prognostic index; LDH, lactate dehydrogenase; PMD, progressive metabolic disease; PMR, partial metabolic response; SD, stable disease; TMTV, total metabolic tumor volume.

We investigated whether the efficacy and safety of axi-cel were consistent among different patient populations, especially in patients aged ≥70 years and with comorbidities defined by a HCT-CI score ≥3. In patients aged ≥70 years (*n* = 33), the 3-month CMR was 72.7% versus 69.0% in patients <70 years (*n* = 29) (Extended Data Table 4). Survival outcomes and DOR were comparable between patients aged <70 years and ≥70 years (Fig. 3). Patients aged ≥70 years did not show increased toxicity compared to those aged <70 years, with similar rates of CRS, ICANS and ICU transfer (Extended Data Table 4). Likewise, patients with a HCT-CI score ≥3 (*n* = 20) reported a 3-month CMR of 80.0%, with similar survival and no increase in toxicity compared to those with a HCT-CI score < 3 (*n* = 42) (Extended Data Table 5). Finally, a similar investigator-assessed CMR at 3 months was observed across all evaluated subgroups but one. The only exception was total metabolic tumor volume (TMTV), as a high TMTV > 80 ml at inclusion was associated with a reduced CMR at 3 months (Extended Data Fig. 4)^26^.

## Discussion

Patients with LBCL who are refractory or who relapse early after first-line chemoimmunotherapy are often chemorefractory and have a very limited cure rate after standard chemoimmunotherapy^27^. The ALYCANTE study is the first to assess the efficacy and safety of axi-cel as a second-line therapy in patients with high-risk R/R LBCL who are deemed ineligible for HDCT/ASCT. In this prospective, multicenter, open-label, phase 2 trial, a single axi-cel infusion was associated with a manageable safety profile and a high antitumor activity. The study met its primary end point with a CMR at 3 months of 71% versus 12% with SOC (second-line chemoimmunotherapy) based on historical controls^6^.

In this patient population with poor prognostic features, including 54.8% with primary refractory disease and 63.4% who were refractory to bridging therapy, treatment with axi-cel resulted in high response rates and durable remissions. The investigator-assessed best ORR and best CMR were 90.3% and 79.0%, respectively. Median PFS was 11.8 months and median OS was not reached. Furthermore, the efficacy of axi-cel was maintained across key subgroups, including patients with high-risk features, such as age ≥70 years, HCT-CI score ≥3, IPI ≥ 3 and primary refractory disease. The only exception was in patients with a high TMTV at inclusion, as we observed a reduced CMR at 3 months among these patients. This observation is consistent with other studies evaluating CAR-T cell therapy for R/R LBCL, in which a high TMTV was associated with an increased risk of early relapse or progression^26,28–31^.

The findings of ALYCANTE are overall consistent with the results of the phase 3 ZUMA-7 trial^8,11^ evaluating axi-cel as second-line therapy in patients with high-risk R/R LBCL deemed eligible for HDCT/ASCT. In ZUMA-7, axi-cel resulted in a CMR of 65% and a median PFS of 14.7 months. At a median follow-up of 24.9 months, median OS was not reached^8^. There are, however, many differences in design and patient populations between ALYCANTE and ZUMA-7 (ref. ^8^). For instance, no bridging therapy was allowed in ZUMA-7, except for corticosteroid use, in contrast to ALYCANTE where bridging with R-GemOx was permitted to reflect real-world clinical practice. Indeed, many patients with aggressive lymphomas cannot be spared from bridging chemoimmunotherapy after leukapheresis^21^. As expected, patients were notably older in the ALYCANTE study than in the ZUMA-7 study with a median age of 70 versus 59 years, respectively. Despite the advanced age and comorbidity burden in ALYCANTE, the observed toxicity of axi-cel was overall consistent with that in ZUMA-7 (ref. ^8^). For instance, the incidences of severe CRS and ICANS were comparable, at 8% and 15% for grade 3–4 CRS and ICANS in ALYCANTE versus 6% and 21% in ZUMA-7, respectively^8^. Likewise, the rate of patients admitted to the ICU was 26% in ALYCANTE and 25% in ZUMA-7. NRM was also similar in ALYCANTE and ZUMA-7 (refs. ^8,11^), at 10% in both studies. It is nevertheless important to note that ZUMA-7 was conducted before the COVID-19 pandemic (between January 2018 and October 2019), whereas ALYCANTE was conducted between March 2021 and May 2022. If we omit the two cases of fatal COVID-19 infection that contribute to one-third of the NRM, the NRM in ALYCANTE would be 6%. This number is in a similar range to a post-marketing cohort study by Nastoupil et al. conducted before the COVID-19 pandemic, in which the NRM was 4% among 275 patients who received axi-cel for R/R LBCL^32^. Overall, the safety and efficacy of axi-cel seem comparable in the ALYCANTE and ZUMA-7 trials, supporting the role of axi-cel as a second-line therapy in a broad population of patients with R/R LBCL, regardless of transplant-eligibility.

Another CD19-directed CAR-T cell product, lisocabtagene maraleucel (liso-cel), has also been evaluated as a second-line therapy in the open-label, phase 2 PILOT study performed in 61 patients with R/R LBCL who are ineligible for ASCT^33^. In PILOT, liso-cel yielded a best ORR and a best CMR, as assessed by an independent review committee, of 80% and 54%, respectively, with a median PFS of 9.0 months^33^. These results are overall consistent with those of ALYCANTE, in which centrally assessed best ORR and best CMR were 92% and 82%, respectively. The results of PILOT also complement the TRANSFORM trial, a phase 3 trial evaluating liso-cel as second-line therapy in patients with R/R LBCL intended to receive HDCT/ASCT^7,10^. In TRANSFORM, at a median follow-up of 17.5 months, the ORR and the CMR were 87% and 74%, respectively and the median PFS was not reached. As previously reported with liso-cel^7,10^, acute toxicities appeared particularly low in the PILOT study, notably grade ≥3 CRS and ICANS (2% and 5% respectively). In PILOT, 15% of patients were admitted to the ICU and 3% experienced NRM^33^. Regarding delayed toxicities, 30% of patients experienced grade ≥3 prolonged cytopenias in PILOT compared to 37% in ALYCANTE.

Despite the comparable study designs and sample sizes of ALYCANTE and PILOT^33^, cross-trial comparisons should be approached with caution, particularly as eligibility criteria differed between these two studies. First, late relapses (beyond 12 months) were allowed in PILOT^33^, whereas only early relapses were allowed in ALYCANTE and TRANSFORM^7,10^. Overall, 25% of patients included in PILOT had late relapses^33^. Second, although ASCT ineligibility in both studies was based on physician’s assessment (subjective assessment), the objective criteria for ASCT ineligibility differed between ALYCANTE and PILOT^33^. In ALYCANTE, patients had to meet at least one of the three protocol-defined criteria for ASCT ineligibility: age ≥65 years, HCT-CI score ≥3, or previous ASCT. On the other hand, patients in PILOT were required to meet at least one of the following criteria to define ASCT ineligibility: age ≥70 years; ECOG performance status of 2; diffusing capacity of the lung for carbon monoxide ≤60%; left ventricular ejection fraction <50%; creatinine clearance <60 ml min^−1^; aspartate aminotransferase (AST) or alanine aminotransferase (ALT) concentrations more than two times the upper limit of normal^33^.

Currently, there are no standard criteria nor consensus to determine whether a patient can undergo HDCT/ASCT. In most countries, a theoretical age limit for HDCT/ASCT is set to 65–70 years. The age cutoff to define ASCT ineligibility differed between ALYCANTE (65 years) and PILOT (70 years); however, in the present study, subgroup analysis demonstrated that efficacy and safety outcomes after axi-cel infusion were overall similar between patients aged <70 years and ≥70 years. Numerous investigations have additionally found that HCT-CI, a well-established prognostic model for comorbidities, can predict survival outcomes for both autologous and allogeneic stem cell transplantation^24,34,35^. A HCT-CI score ≥3 was shown to be independently associated with a higher risk of NRM (*P* < 0.001) and shorter survival (*P* = 0.03) among recipients of ASCT^34,35^. In ALYCANTE, the efficacy and safety of axi-cel were not altered in patients with high HCT-CI scores.

This study is limited by its single-arm design with no active control group. Therefore, selection bias cannot be ruled out. Although axi-cel compares favorably to second-line chemoimmunotherapy based on historical controls^6^, it remains to be compared to more recent regimens such as Tafa-Len, Pola-BR and bispecific antibodies^15,16,36–43^. Our study is also limited by a small sample size and a relatively short follow-up duration (median of 12.0 months); however, the ALYCANTE trial is still ongoing, with a planned follow-up of up to 3 years per patient to determine the long-term efficacy and safety of axi-cel in this patient population.

In conclusion, axi-cel as a second-line treatment resulted in high response rates and durable remissions in patients with high-risk R/R LBCL with poor prognostic features and who were not eligible for HDCT/ASCT. Moreover, despite advanced age and comorbidities, axi-cel had an acceptable safety profile in this population considered unfit for HDCT/ASCT. Together, these results support axi-cel as a second-line treatment in patients with R/R LBCL who are deemed ineligible for HDCT/ASCT.

## Methods

### Study design

ALYCANTE (ClinicalTrials.gov ID NCT04531046) is an ongoing prospective, single-arm, multicenter, open-label, phase 2 trial. Study participants were enrolled in 18 centers across France. The full study protocol is provided in the Supplementary Information. Supplementary Table 1 provides information on the ALYCANTE study team as well as the study’s investigators and co-investigators.

### Inclusion and ethics

The study protocol was approved by French Ethics Committee Est I (Dijon) N°20.07.08.66206, in accordance with applicable French laws and regulations. The study was performed in accordance with the Declaration of Helsinki and the International Conference on Harmonization Good Clinical Practice guidelines. Written informed consent was obtained from each participant before any study procedure.

### Participants

Eligible patients were aged 18 years or older with histologically confirmed aggressive B cell non-Hodgkin lymphoma, diagnosed according to the 2016 World Health Organization classification criteria^44^, as DLBCL, high-grade B cell lymphoma or follicular lymphoma grade 3B. Disease had to be refractory to or had relapsed no more than 12 months after the completion of first-line chemoimmunotherapy containing a monoclonal CD20 antibody and an anthracycline-containing regimen (CHOP or CHOP-like regimen). Refractory disease was defined as a lack of complete response to first-line therapy and relapsed disease as biopsy-proven disease relapse within 12 months from completion of first-line therapy. Patients must also have been ineligible for HDCT/ASCT based on a physician’s assessment and with at least one of the following criteria: age ≥65 years, HCT-CI score ≥3 (as reported by investigators)^24^ or previous ASCT (as first-line consolidation). Patients were enrolled regardless of sex, which was collected according to the identity information provided by the patients.

There were in total, three important protocol deviations from the inclusion criteria in the present study, which were all related to relapsed disease longer than 12 months after first-line chemoimmunotherapy. For two patients, the central histological diagnosis was not aggressive B cell non-Hodgkin lymphoma but grade 1–3A follicular lymphoma.

Patients were deemed eligible for CAR-T cell therapy based on a physician’s assessment and all of the following:

1. ECOG performance status of 0, 1 or 2.
2. Adequate vascular access for leukapheresis (peripheral or central venous line).
3. Absolute neutrophil count ≥1.0 × 10^9^ l^−1^.
4. Platelet count ≥75 × 10^9^ l^−1^.
5. Absolute lymphocyte count ≥0.1 × 10^9^ l^−1^.
6. Creatinine clearance (according to the Cockcroft–Gault equation or the modification of diet in renal disease equation) ≥40 ml min^−1^.
7. Serum ALT/AST ≤ 2.5 × upper limit of normal.
8. Total bilirubin ≤26 μmol l^−1^, except in patients with Gilbert’s syndrome.
9. Left ventricular ejection fraction ≥45%.
10. Oxygen saturation ≥92% on room air.

Key exclusion criteria included:

1. Receipt of more than one prior line of systemic therapy.
2. Previous CD19-targeted therapy.
3. Cardiac involvement.
4. Requirement for urgent therapy due to tumor mass effects such as bowel obstruction or blood vessel compression.
5. Clinically significant pleural effusion.
6. Known central nervous system disease.
7. History of cardiovascular disease within the past 6 months.
8. History of autoimmune disease requiring systemic immunosuppression and/or disease-modifying agents within the last year.
9. History of idiopathic pulmonary fibrosis, organizing pneumonia, drug-induced pneumonitis, idiopathic pneumonitis or evidence of active pneumonitis on a chest computed tomography scan at screening.
10. Active hepatitis B or C infection or positive HIV serology at the time of screening.

### Procedures

Extended Data Fig. 1 provides an overview of the study procedures. Within 15 days after screening, all eligible patients underwent leukapheresis to obtain enough peripheral blood mononuclear cells to produce axi-cel. Optional bridging therapy after leukapheresis consisting of 1–2 cycles of R-GemOx (rituximab at 375 mg m^−2^, gemcitabine at 1,000 mg m^−2^ and oxaliplatin at 100 mg m^−2^, intravenously administered every 2 weeks for one or two cycles), corticosteroids (type and dose at the investigator’s discretion) or both was allowed. Upon the availability of axi-cel, patients received lymphodepleting conditioning chemotherapy for 3 days with cyclophosphamide (at a dose of 500 mg m^−2^ d^−1^) and fludarabine (30 mg m^−2^ d^−1^), followed 2–7 days later by a single intravenous infusion of axi-cel (at a target dose of 2 × 10^6^ CAR-T cells per kilogram of body weight). Before lymphodepleting chemotherapy was administered, reconfirmation of study eligibility, including positron emission tomography (PET)-positive disease, was required. Hence, disease assessment by PET was performed at screening, within 7 days before the start of lymphodepleting chemotherapy, as well as at 1, 3, 6, 9 and 12 months after the axi-cel infusion. Clinical examination (including ECOG performance status), blood sampling and laboratory tests (including complete blood count and serum chemistry) were also performed at screening, on the day of axi-cel infusion, daily between day 1 and day 10 after the axi-cel infusion, on day 14 and at 1, 3, 6, 9 and 12 months following the axi-cel infusion. Patients were followed-up for 3 years after the axi-cel infusion and could then consent to participate in a long-term follow-up for up to 15 years via DESCAR-T (ClinicalTrials.gov ID NCT04328298), a French register of patients with malignant hemopathies eligible for CAR-T therapy.

Safety was monitored continuously throughout the study. CAR-T cell toxicities such as CRS and ICANS were graded according to the American Society for Transplantation and Cellular Therapy grading system^45^. All other Adverse effects were graded according to the National Cancer Institute Common Terminology Criteria for Adverse Events (CTCAE v.5.0).

### Outcomes

The primary end point was investigator-assessed CMR at 3 months from the axi-cel infusion according to the Lugano response criteria^25^.

Secondary efficacy end points included investigator-assessed ORR at 3 months from the axi-cel infusion, investigator-assessed CMR at 6 months from the axi-cel infusion and the best investigator-assessed ORR and CMR. CMR and ORR at 3 months from the axi-cel infusion were also assessed by a central review panel formed by two readers and an adjudicator. Other secondary efficacy outcomes included the best ORR and best CMR as assessed by the central review panel, DOR, EFS from leukapheresis, PFS from infusion and OS from infusion. DOR, EFS and PFS were investigator-assessed. DOR was defined as the time from attainment of PMR or CMR to the date of first documented disease progression/relapse or death from any cause. EFS was defined as the time between leukapheresis and any event preventing axi-cel infusion if axi-cel was never infused, or death, disease progression or instauration of a new lymphoma therapy for lymphoma progression after axi-cel infusion. PFS was defined as the time from axi-cel infusion to disease progression or death from any cause. OS was defined as the time from axi-cel infusion to death from any cause. The estimated rates of EFS, PFS and OS at 6 and 12 months were further evaluated.

Safety was evaluated as the incidence, nature and severity of adverse effects. Adverse effects of special interest were related to CAR-T cell toxicities and included CRS and ICANS. Mortality during the study was summarized by cause of death.

### Statistical analysis

A one-sample binomial design was used to calculate the initial sample size. We hypothesized that axi-cel would yield a CMR at 3 months of 34% compared to 12% with a historical SOC estimated from a retrospective, real-world cohort^6^. On the basis of this assumption, the initial sample size was calculated to be 40 infused patients, with 96% power and a 0.05 α-level (one-sided). To enable a balanced comparison of the efficacy of axi-cel in different age subgroups (<70 and ≥70 years), with a power of 85% and considering potential dropouts, the required sample size was increased to 62 patients.

Efficacy and safety analyses were performed on the mFAS, which included all patients who signed an informed consent and were infused with axi-cel. A sensitivity analysis of the primary end point was also conducted on all patients who signed an informed consent and had leukapheresis (FAS). We calculated CMR with exact Clopper–Pearson CI values, without adjustment for multiplicity. Patients without response assessment, due to any reason, were considered as nonresponders. We used the Kaplan–Meier method to estimate medians and 95% CI values for PFS, EFS, OS and DOR. If patients did not have an event at the time of the PFS, EFS and DOR analysis, they were censored at the date of the last disease assessment. For assessment of OS, alive patients were censored at their last follow-up date. Safety end points were assessed using descriptive statistics (counts and percentages). A subgroup analysis of CMR at 3 months was conducted for prespecified covariates (such as sex) and a Forest plot was provided.

Collected data were entered using the Electronic Data Capture system from Ennov v.8.1 (Ennov). Sample size calculation was performed using EAST v.6.5 (Cytel). All statistical analyses were performed using SAS v.9.3 or higher (SAS Institute) and AdClin v.3.2.2 or higher (AdClin).

### Reporting summary

Further information on research design is available in the Nature Portfolio Reporting Summary linked to this article.

## Online content

Any methods, additional references, Nature Portfolio reporting summaries, source data, extended data, supplementary information, acknowledgements, peer review information; details of author contributions and competing interests; and statements of data and code availability are available at 10.1038/s41591-023-02572-5.

### Supplementary information

Supplementary Information Supplementary Table 1 and Study Protocol.

Reporting Summary

## Figures and Tables

**Extended Data Table 1:**
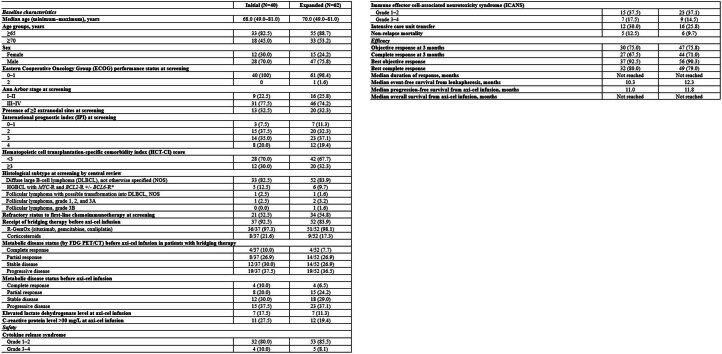
Patient characteristics and outcomes for the initial (N = 40) and expanded (N = 62) cohorts

Data are n (%), unless otherwise specified.

*All samples previously diagnosed as high-grade B cell lymphoma (double-hit/triple-hit/not otherwise specified) were classified as HGBCL with *MYC*-R and *BCL2*-R +/− *BCL6*-R (high-grade B cell lymphoma with *MYC* and *BCL2* rearrangements associated or not with *BCL6* rearrangement) after central review.

**Extended Data Table 2:**
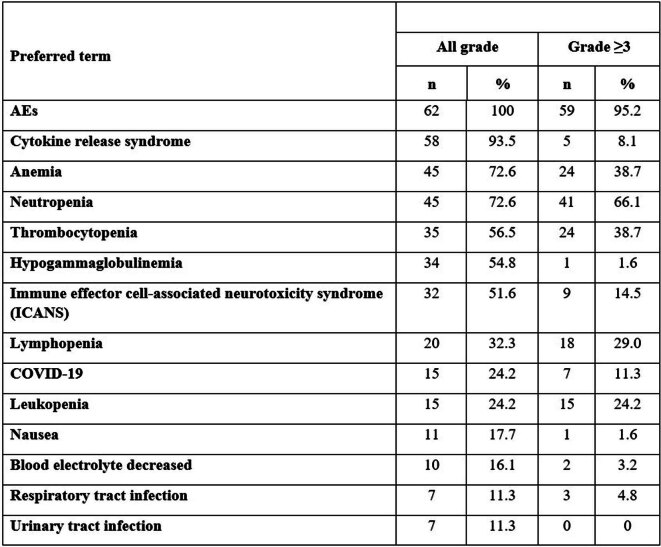
Most frequent (in more than 10% of patients) adverse events in the modified full analysis set (N = 62)

**Extended Data Table 3:**
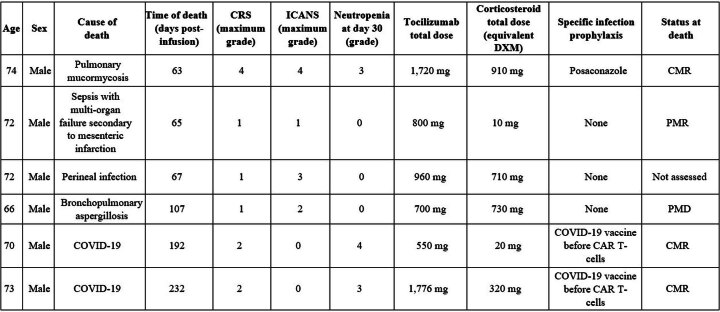
Patients with fatal adverse events

Abbreviations: CAR, chimeric antigen receptor; CMR, complete metabolic response; CRS, cytokine release syndrome; DXM, dexamethasone; ICANS, immune effector cell-associated neurotoxicity syndrome; PMD, progressive metabolic disease; PMR, partial metabolic response.

**Extended Data Table 4:**
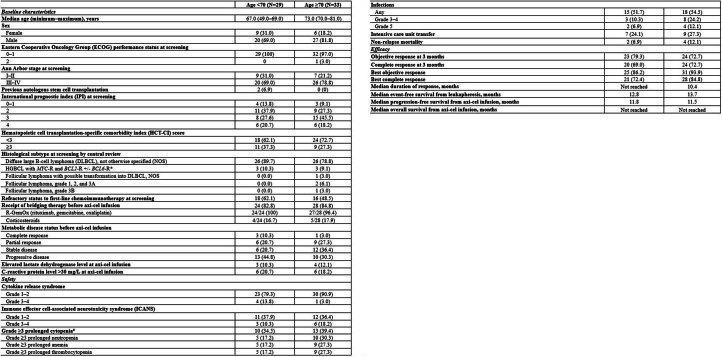
Patient characteristics and outcomes according to age (<70 *versus* ≥ 70 years)

Data are n (%), unless otherwise specified.

*All samples previously diagnosed as high-grade B-cell lymphoma (double-hit/triple-hit/not otherwise specified) were classified as HGBCL with *MYC*-R and *BCL2*-R +/− *BCL6*-R (high-grade B-cell lymphoma with *MYC* and *BCL2* rearrangements associated or not with *BCL6* rearrangement) after central review.

^#^Grade ≥3 prolonged cytopenia was defined as a grade ≥3 laboratory result of anemia, neutropenia, and/or thrombocytopenia not resolved on day 30 after axi-cel infusion.

**Extended Data Table 5:**
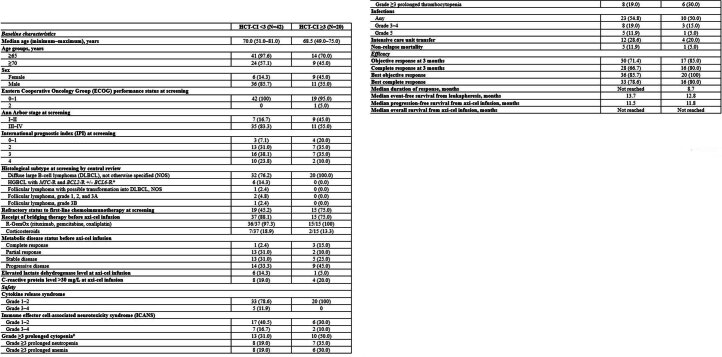
Patient characteristics and outcomes according to hematopoietic cell transplantation-specific comorbidity index (HCT-CI) (<3 *versus* ≥ 3)

Data are n (%), unless otherwise specified.

*All samples previously diagnosed as high-grade B-cell lymphoma (double-hit/triple-hit/not otherwise specified) were classified as HGBCL with *MYC*-R and *BCL2*-R +/− *BCL6*-R (high-grade B-cell lymphoma with *MYC* and *BCL2* rearrangements associated or not with *BCL6* rearrangement) after central review.

^#^Grade ≥3 prolonged cytopenia was defined as a grade ≥3 laboratory result of anemia, neutropenia, and/or thrombocytopenia not resolved on day 30 after axi-cel infusion.

## Data Availability

This trial is currently ongoing. Requests for access to aggregate data and supporting clinical documents will be reviewed and approved by an independent review panel on the basis of scientific merit. The datasets generated and/or analyzed during the current study are not publicly available due to proprietary considerations. All data provided are anonymized to respect the privacy of patients who have participated in the trial, in line with applicable laws and regulations. Data requests pertaining to the manuscript may be made to the corresponding author (R.H.; roch.houot@chu-rennes.fr). Requests will be processed within 12 weeks.
