# Publisher Correction: Axicabtagene ciloleucel as second-line therapy in large B cell lymphoma ineligible for autologous stem cell transplantation: a phase 2 trial

**DOI:** 10.1038/s41591-023-02624-w

**Published:** 2023-10-09

**Authors:** Roch Houot, Emmanuel Bachy, Guillaume Cartron, François-Xavier Gros, Franck Morschhauser, Lucie Oberic, Thomas Gastinne, Pierre Feugier, Rémy Duléry, Catherine Thieblemont, Magalie Joris, Fabrice Jardin, Sylvain Choquet, Olivier Casasnovas, Gabriel Brisou, Morgane Cheminant, Jacques-Olivier Bay, Francisco Llamas Gutierrez, Cédric Menard, Karin Tarte, Marie-Hélène Delfau, Cédric Portugues, Emmanuel Itti, Xavier Palard-Novello, Paul Blanc-Durand, Yassine Al Tabaa, Clément Bailly, Camille Laurent, François Lemonnier

**Affiliations:** 1 Department of Hematology, University Hospital of Rennes, UMR U1236, INSERM, University of Rennes, French Blood Establishment, Rennes, France; 2 Department of Hematology, Lyon Sud Hospital Center, INSERM U1111, Lyon, France; 3 Department of Hematology, University Hospital of Montpellier, UMR-CNRS 5535, Montpellier, France; 4 Department of Clinical Hematology and Cellular Therapy, University Hospital of Bordeaux, Bordeaux, France; 5 ULR 7365 - GRITA, University Hospital of Lille, Lille, France; 6 Department of Hematology, Cancer University Institute of Toulouse Oncopole, Toulouse, France; 7 Department of Hematology, University Hospital of Nantes, Nantes, France; 8 Department of Hematology, University Hospital of Nancy, INSERM 1256, University of Lorraine, Vandœuvre-lès-Nancy, France; 9 Department of Clinical Hematology and Cellular Therapy, Sorbonne University, Saint-Antoine Hospital, AP-HP, INSERM UMR938, Paris, France; 10 Department of Hematology and Oncology, Saint-Louis Hospital, AP-HP, Paris, France; 11 Department of Hematology, University Hospital of Amiens, Amiens, France; 12 Department of Clinical Hematology, Henri Becquerel Center, INSERM U1245, Rouen, France; 13 Department of Hematology, University Hospital Pitié Salpêtrière, AP-HP, Sorbonne University, Paris, France; 14 Department of Clinical Hematology, Dijon University Hospital, INSERM UMR1231, Dijon, France; 15 Department of Hematology, Institut Paoli-Calmettes, Marseille, France; 16 Department of Clinical Hematology, Necker-Enfants Malades University Hospital, AP-HP, INSERM UMR1163, Paris, France; 17 Department of Clinical Hematology and Cellular Therapy, Clermont-Ferrand University Hospital Center, Clermont-Ferrand, France; 18 Department of Anatomopathology, University Hospital of Rennes, Rennes, France; 19 French Blood Establishment and SITI Laboratory, UMR U1236, INSERM, University of Rennes, University Hospital Center of Rennes, Rennes, France; 20 Department of Immunology, Henri Mondor Hospital, Créteil, France; 21 Department of Biostatistics, LYSARC, Lyon-Sud Hospital, Pierre-Bénite, France; 22 Department of Nuclear Medicine, Henri Mondor Hospital, Créteil, France; 23 Department of Nuclear Medicine, University of Rennes, CLCC Eugène Marquis, INSERM, Rennes, France; 24 Department of Nuclear Medicine, CHU H. Mondor, U-PEC, AP-HP, Créteil, France; 25 Scintidoc Nuclear Medicine Center, Montpellier, France; 26 Nantes-Angers Cancer Research Center CRCI2NA, University of Nantes, INSERM UMR1307, CNRS-ERL6075, Nantes, France; 27 Department of Pathology, Cancer University Institute of Toulouse Oncopole, CHU Toulouse, CRCT INSERM U1037, Toulouse, France; 28 Lymphoid Malignancies Unit, Henri Mondor Hospital, Mondor Institute for Biomedical Research, INSERM U955, University Paris-Est, Créteil, France

**Keywords:** Phase II trials, B-cell lymphoma, Cancer immunotherapy

Correction to: *Nature Medicine* 10.1038/s41591-023-02572-5, published online 14 September 2023.

In the version of this article initially published, the title of the paper, now reading “Axicabtagene ciloleucel as second-line therapy in large B cell lymphoma ineligible for autologous stem cell transplantation: a phase 2 trial,” appeared incorrectly as “Axicabtagene ciloleucel in large B cell lymphoma ineligible for autologous stem cell transplantation: the phase 2 ALYCANTE trial.” The title has been updated in the HTML and PDF versions of the article.

