# Author Correction: Axicabtagene ciloleucel as second-line therapy in large B cell lymphoma ineligible for autologous stem cell transplantation: a phase 2 trial

**DOI:** 10.1038/s41591-024-03053-z

**Published:** 2024-05-14

**Authors:** Roch Houot, Emmanuel Bachy, Guillaume Cartron, François-Xavier Gros, Franck Morschhauser, Lucie Oberic, Thomas Gastinne, Pierre Feugier, Rémy Duléry, Catherine Thieblemont, Magalie Joris, Fabrice Jardin, Sylvain Choquet, Olivier Casasnovas, Gabriel Brisou, Morgane Cheminant, Jacques-Olivier Bay, Francisco Llamas Gutierrez, Cédric Menard, Karin Tarte, Marie-Hélène Delfau, Cédric Portugues, Emmanuel Itti, Xavier Palard-Novello, Paul Blanc-Durand, Yassine Al Tabaa, Clément Bailly, Camille Laurent, François Lemonnier

**Affiliations:** 1 Department of Hematology, University Hospital of Rennes, UMR U1236, INSERM, University of Rennes, French Blood Establishment, Rennes, France; 2 Department of Hematology, Lyon Sud Hospital Center, INSERM U1111, Lyon, France; 3 Department of Hematology, University Hospital of Montpellier, UMR-CNRS 5535, Montpellier, France; 4 Department of Clinical Hematology and Cellular Therapy, University Hospital of Bordeaux, Bordeaux, France; 5 ULR 7365 - GRITA, University Hospital of Lille, Lille, France; 6 Department of Hematology, Cancer University Institute of Toulouse Oncopole, Toulouse, France; 7 Department of Hematology, University Hospital of Nantes, Nantes, France; 8 Department of Hematology, University Hospital of Nancy, INSERM 1256, University of Lorraine, Vandœuvre-lès-Nancy, France; 9 Department of Clinical Hematology and Cellular Therapy, Sorbonne University, Saint-Antoine Hospital, AP-HP, INSERM UMR938, Paris, France; 10 Department of Hematology and Oncology, Saint-Louis Hospital, AP-HP, Paris, France; 11 Department of Hematology, University Hospital of Amiens, Amiens, France; 12 Department of Clinical Hematology, Henri Becquerel Center, INSERM U1245, Rouen, France; 13 Department of Hematology, University Hospital Pitié Salpêtrière, AP-HP, Sorbonne University, Paris, France; 14 Department of Clinical Hematology, Dijon University Hospital, INSERM UMR1231, Dijon, France; 15 Department of Hematology, Institut Paoli-Calmettes, Marseille, France; 16 Department of Clinical Hematology, Necker-Enfants Malades University Hospital, AP-HP, INSERM UMR1163, Paris, France; 17 Department of Clinical Hematology and Cellular Therapy, Clermont-Ferrand University Hospital Center, Clermont-Ferrand, France; 18 Department of Anatomopathology, University Hospital of Rennes, Rennes, France; 19 French Blood Establishment and SITI Laboratory, UMR U1236, INSERM, University of Rennes, University Hospital Center of Rennes, Rennes, France; 20 Department of Immunology, Henri Mondor Hospital, Créteil, France; 21 Department of Biostatistics, LYSARC, Lyon-Sud Hospital, Pierre-Bénite, France; 22 Department of Nuclear Medicine, Henri Mondor Hospital, Créteil, France; 23 Department of Nuclear Medicine, University of Rennes, CLCC Eugène Marquis, INSERM, Rennes, France; 24 Department of Nuclear Medicine, CHU H. Mondor, U-PEC, AP-HP, Créteil, France; 25 Scintidoc Nuclear Medicine Center, Clinique Clémentville, Montpellier, France; 26 Nantes-Angers Cancer Research Center CRCI2NA, University of Nantes, INSERM UMR1307, CNRS-ERL6075, Nantes, France; 27 Department of Pathology, Cancer University Institute of Toulouse Oncopole, CHU Toulouse, CRCT INSERM U1037, Toulouse, France; 28 Lymphoid Malignancies Unit, Henri Mondor Hospital, Mondor Institute for Biomedical Research, INSERM U955, University Paris-Est, Créteil, France

**Keywords:** Phase II trials, B-cell lymphoma, Cancer immunotherapy, Phase II trials, B-cell lymphoma, Cancer immunotherapy

Correction to: *Nature Medicine* 10.1038/s41591-023-02572-5, published online 14 September 2023

In the version of the article initially published, the affiliation of Yassine Al Tabaa was incomplete and has now been updated to read “Scintidoc Nuclear Medicine Center, Clinique Clémentville, Montpellier, France” in the HTML and PDF versions of the article.

