# Supplementary material for: Axicabtagene ciloleucel as second-line therapy in large B cell lymphoma ineligible for autologous stem cell transplantation: a phase 2 trial
Source: Nat Med. 2023 Sep 14;29(10):2593–601. doi: 10.1038/s41591-023-02572-5 (PMC10579056; doi:10.1038/s41591-023-02572-5)
Supplement: Supplementary file 1 — Supplementary Table 1 and Study Protocol. [file 41591_2023_2572_MOESM1_ESM.pdf]

nature medicine

Article https://doi.org/10.1038/s41591-023-02572-5

Axicabtagene ciloleucel as second-line therapy in large B cell lymphoma ineligible for autologous stem cell transplantation: a phase 2 trialIn the format provided by the authors and unedited

Supplementary Table 1. ALYCANTE study team and investigators/co-investigators

**ALYCANTE/LYSARC study team**	
**Name**	**Department**
Dr Florence BROUSSAIS	Medical department
Dr Pascale CONY-MAKHOUL	
Dr Fabienne DI GIAMBATTISTA	
Loic CHARTIER	Biostatistics department
Karina DORDONNE	
Jérôme PAGET	
Cedric PORTUGUES	
Micipsa AIT MOKHTAR	Monitoring department
Yael BIKATT-BIMAI	
Fida BOUGHATTAS	
Monique GRANDJEAN	
Mohamed KAROUNE	
Déborah LIGOUT	
Laetitia MELGAR	
Valentin MOINE	
Clémence PACAULT	
Mélanie PARAS	
Olivier RAMON	
Emmanuelle ROBERT-EYDOUX	
Dounya BOUDEBOUZE	Project management department
Elisa CHAREYRE	
Cyrielle DE LACHEISSERIE	
Delphine GERMAIN	
Camille JACQUINOT	
Stéphanie PICARD	
Marie-Christine PINAIL	
Véronique VACHER	
Catherine VERNADE	
Clémence CAPRON	Data management department
Sophie CHAMBRIARD	
Lucie BONNEFOY	Pharmacovigilance department
Pauline DJAKER	

Anaïs EL HACHEMI	
Valentina FUENZALIDA-VASQUEZ	
Clémence PONCET	
Juliette SUN	
Anne-Laure BOREL	Biology and histopathology department
Myriem CHIKHAOUI	
Carine DESCHODT	
Soumeia GREOUI	
Véronique JALOUX	
Juliette JOASSAINT	
Alexia SIMON	
Nadine VAILHEN	
Romain RICCI	Imaging department
Emelie VAN ZELE	
Marie-France COHEN	Regulatory and quality assurance department
Virginie DAVID	
Lise JACQUEROUX	
Linda KAINOU	
Satenik STEPANIAN	
Ombeline VERITE	
Anne VIOLA	
**List of investigators and co-investigators**	
**Name**	**Location**
Pr François LEMONNIER	Créteil
Dr Romain GOUNOT	
Dr Dehbia MENOUCHE	
Dr Fabien LE BRAS	
Dr Karim BELHADJ	
Pr Corinne HAIOUN	
Pr Catherine THIEBLEMONT	Paris
Dr Charlotte SCHMIDT-HIEBER	
Dr Loïc RENAUD	
Dr Anne BRIGNIER	
Dr Daphné KRZISCH	
Dr Hannah MOATTI	
Dr Roberta DI BLASI	

Dr Pauline BRICE	
Dr Alexandra JUDET	
Dr Nathalie PARQUET	
Pr Emmanuel BACHY	Pierre-Bénite
Dr Alizée MAAREK	
Dr Emmanuelle FERRANT	
Dr Hélène LEQUEU	
Dr Hervé GHESQUIERES	
Dr Pierre SESQUES	
Dr Violaine SAFAR	
Dr Guillaume AUSSEDAT	
Dr Camille GOLFIER	
Dr Anne LAZARETH	
Dr Lionel KARLIN	
Dr Lucie OBERIC	Toulouse
Dr Pierre BORIES	
Dr Caroline PROTIN	
Dr Loic YSEBAERT	
Dr Noémie GADAUD	
Dr Sarah GUENOUNOU	
Dr Cécile BOREL	
Pr Fabrice JARDIN	Rouen
Dr Mustafa ALANI	
Dr Florian BOUCLET	
Dr Anne-Lise MENARD	
Dr Hélène LANIC	
Dr Aspasia STAMATOULLAS	
Dr Stéphane LEPRETRE	
Dr Oana BREHAR	
Dr Vincent CAMUS	
Dr Emilie LEMASLE	
Dr Pascal LENAIN	
Dr Nathalie CONTENTIN	
Pr Hervé TILLY	
Dr Sylvain CHOQUET	Paris
Dr Silvia SOLORZANO GIMENEZ	
Dr Nicolas GAUTHIER	
Dr Damien ROOS-WEIL	

Dr Véronique MOREL-MALEK	
Dr Nathalie JACQUE	
Dr Maya OUZEGDOUH	
Pr Franck MORSCHHAUSER	Lille
Dr David BEAUVAIS	
Dr Sabine TRICOT	
Dr Jean-Baptiste BOSSARD	
Dr Micha SROUR	
Dr Olivier CASASNOVAS	Dijon
Dr Camille FAVENNEC	
Dr Alexandre PAYSSOT	
Dr Philippine ROBERT	
Dr Marie-Lorraine CHRETIEN	
Dr Ingrid LAFON	
Dr Jean-Noël BASTIE	
Dr Denis CAILLOT	
Dr Steeve CHEVREUX	
Pr Pierre FEUGIER	Vandœuvre-lès-Nancy
Dr Arnaud CAMPIDELLI	
Dr Caroline JACQUET	
Dr Luciane SCHIRMER	
Dr Marie-Thérèse RUBIO	
Dr Gabrielle ROTH GUEPIN	
Dr Suzanne MATHIEU-NAFISSI	
Dr Gabriel BRISOU	Marseille
Dr Nathalie CHARRIER	
Dr Sabine FURST	
Dr Morgane CHEMINANT	Paris
Dr Ilhem RAHAL	
Pr Roch HOUOT	Rennes
Dr Sophie DE GUIBERT	
Dr Guillaume MANSON	
Dr Olivier DECAUX	
Dr Tony MARCHAND	
Dr Faustine LHOMME	
Dr Pierre DAUFRESNE	
Dr Jean-Baptiste MEAR	

Dr Aline MOIGNET AUTREL	
Dr Martine ESCOFFRE-BARBE	
Dr Marc BERNARD	
Dr Stanislas NIMUBONA	
Pr Thierry LAMY DE LA CHAPELLE	
Dr Magalie JORIS	Amiens
Pr Jean-Pierre MAROLLEAU	
Dr Lina MUSTAPHA	
Dr Etienne PAUBELLE	
Dr Amandine CHARBONNIER	
Dr Delphine LEBON	
Dr Lavinia MERLUSCA	
Pr Guillaume CARTRON	Montpellier
Dr Hanane GUEDON	
Dr Robert NAVARRO	
Dr Charles HERBAUX	
Dr Eve GEHLKOPF	
Dr Jean-Jacques TUDESQ	
Dr Philippe QUITTET	
Dr Emmanuelle TCHERNONOG	
Dr Tarik KANOUNI	
Dr Thomas GASTINNE	Nantes
Pr Steven LE GOUILL	
Dr Sophie VANTYGHEM	
Dr Viviane DUBRUILLE	
Dr Benoit TESSOULIN	
Dr Thomas GASTINNE	
Dr Cyrille TOUZEAU	
Dr Rémy DULERY	Paris
Dr Florent MALARD	
Dr Anne VEKHOFF	
Dr Eolia BRISSOT	
Dr Simona SESTILI	
Pr Mohamad MOHTY	
Dr François-Xavier GROS	Pessac
Dr Kamal-Krimo BOUABDALLAH	

Dr Edouard FORCADE	
Dr Nathan MOTTAL	
Dr Carmen BOTELLA GARCIA	
Dr Titouan CAZAUBIEL	
Dr Harmony LEROY	
Dr Pierre-Yves DUMAS	
Pr Jacques-Olivier BAY	Clermont-Ferrand
Dr Carine CHALETEIX	
Dr Justyna KANOLD	
Dr Doriane CAVALIERI	
Dr Eric HERMET	
Dr Amandine FAYARD	
Dr Victoria CACHEUX	
Dr Anne-Pascale GRANDJEAN	
Dr Aurélie RAVINET	
Dr Sébastien BAILLY	
Dr Cécile MOLUCON-CHABROT	
Dr Benoit DE RENZIS	
Pr Romain GUIEZE	
Pr Olivier TOURNILHAC	

DocuSign Envelope ID: 4C1EC433-1B7A-4CD2-B02E-B91666107FC1

ALYCANTE

(**A**xi-Cel in **LY**mphoma **CA**R eligible but **N**on **T**ransplant **E**ligible)

Phase 2, Open-Label Study Evaluating Axi-Cel as a 2nd line therapy in patients with Relapsed/Refractory aggressive B-NHL who are ineligible to Autologous Stem Cell Transplantation

A STUDY SPONSORED BY:

LYSARC

THE LYMPHOMA ACADEMIC RESEARCH ORGANISATION

Centre Hospitalier Lyon Sud - Bâtiment 2D CENS-ELI

69495 PIERRE BÉNITE Cedex – France

**COORDINATING INVESTIGATOR**<br>**CO-COORDINATING INVESTIGATOR**	Prof Roch Houot<br>Dr François Lemonnier
**IMAGING COORDINATORS**	Prof Emmanuel Itti<br>Dr Clément Bailly
**BIOLOGICAL COORDINATORS**	Prof Karin Tarte<br>Prof Marie-Hélène Delfau
**PATHOLOGICAL COORDINATOR**	Prof Camille Laurent<br>Dr Francisco Llamas Gutierrez
**COORDINATION SITE**	LYSARC

**REGISTRATION (SEE SECTION 11-1)**	https://lysarc.ennov.com/EnnovClinical/login?etude=ALYCANTE
**SAE REPORTING (SEE SECTION 14)**	Fax to +33 (0)3 59 11 01 86

Version and date of Protocol: Version 4.0 dated 02-February-2023

EudraCT number: 2020-001868-28

CONFIDENTIALITY STATEMENT

The information contained in this document is the property of The Lymphoma Academic Research Organisation (LYSARC) and therefore is provided to you in confidence for review by you, your staff, an applicable Ethics Committee/Institutional Review and regulatory authorities. It is understood that the information will not be disclosed to others without prior written approval from LYSARC, except to the extent necessary to obtain informed consent from persons who may participate to the study.

PROTOCOL APPROVAL & SIGNATURE PAGE

ALYCANTE

PHASE 2, OPEN-LABEL STUDY
EVALUATING AXI-CEL AS A 2ND LINE THERAPY
IN PATIENTS WITH RELAPSED/REFRACTORY AGGRESSIVE B-NHL
WHO ARE INELIGIBLE TO AUTOLOGOUS STEM CELL TRANSPLANTATION

LYSARC

DocuSigned by:
Dr Pascal Bilbault
82549D050143400...

03-02-2023

Name: Dr Pascal BILBAULT **Date**
Title: General Manager

COORDINATING INVESTIGATORS

DocuSigned by:
Pr. Roch HOUOT
7AF35745AAE64B5...

07-02-2023

Name: Prof Roch HOUOT **Date**
Title: Coordinating Investigator

DocuSigned by:
François Lemonnier
294F8B7174C441A...

13-02-2023

Name: Prof François LEMONNIER **Date**
Title: Co-Coordinating Investigator

1 SYNOPSIS

**Sponsor**	LYSARC
**Study name/code**	**ALYCANTE** (**A**xi-Cel in **LY**mphoma **C**AR eligible but **N**on **T**ransplant **E**ligible)
**Study title**	Phase 2, Open-Label Study evaluating Axi-Cel as a 2nd line therapy in patients with Relapsed/Refractory aggressive B-NHL who are ineligible to Autologous Stem Cell Transplantation
**Identification # (EudraCT)**	2020-001868-28
**Protocol version**	Version 3.0
**Development phase**	Phase 2
**Investigational product**	Axicabtagene ciloleucel
**Coordinating investigator**<br>**Co-coordinating investigator**	Pr Roch Houot, MD, PhD, Rennes University Hospital, Rennes, France<br>Dr François Lemonnier, MD, PhD, Henri Mondor Hospital, Créteil, France
**Sites**	20 LYSA Centers in France and Belgium
**Rationale**	The addition of rituximab to CHOP chemotherapy (R-CHOP) has improved the outcome of patients with aggressive B-cell lymphoma. However, patients who are relapsing or are refractory after a rituximab containing first-line therapy have a very poor prognosis. Thus, improving outcome of patients with relapsed or refractory aggressive lymphoma represents an unmet medical need.<br>Axicabtagene ciloleucel (Yescarta®) is a chimeric antigen receptor (CAR) T-cell therapy directed against CD19 which has been approved for the treatment of relapse/refractory diffuse large B-cell lymphoma DLBCL and primary mediastinal large B-cell lymphoma (PMBCL) after 2 or more lines of systemic therapy. In the pivotal ZUMA-1 study, the best ORR was 83% with a complete response (CR) rate of 58%. The CR rate at month 3 post-infusion was 42%. After a median follow-up of 2 years, 37% of patients remained in CR, Progression-free survival (PFS) was 49%, 44% and 39% at 6, 12 and 24 months, respectively; and overall survival (OS) was 78%, 60% and 51% at 6, 12 and 24 months, respectively. Interestingly, several analysis suggest that efficacy and safety are similar in elderly patients. In a post-hoc analysis of the ZUMA-1 trial, patients ≥ 65 years (N=27) had increased CR (75% vs 53%, respectively), duration of response (median 12 vs 8.1 months, respectively), PFS (median 13.2 vs 5.6 months, respectively), and OS (54% vs 49% at 2 years, respectively) compared to patients <65 years (N=81). Elderly patients also experienced less severe cytokine release syndrome (CRS) (7% vs 12% grade ≥3, respectively) although they developed more frequently severe neurotoxicity (44% vs 28% grade≥3, respectively). Real-world evidence from the Center for International Blood and Marrow Transplant Research (CIBMTR) registry demonstrated that the efficacy and safety of Axicabtagene ciloleucel was comparable in DLBCL patients who were ≥ 65 years (N=196) and those who were <65 years (N=337). In patients who were 65 years old or above, the PFS at 6 months was ≈ 60%. Importantly, most patients after the age of 65 cannot tolerate ASCT while many remain eligible to CAR-T cell therapy. Furthermore, administrating CAR T-cells earlier in the therapeutic strategy may be beneficial to patients. Indeed, subset analysis done in the ZUMA-1 trial demonstrated that DLBCL patients who have received more lines of prior therapy tended to experience lower CAR T-cell expansion, less efficacy and more toxicity compared to those who were treated earlier.<br>Here, we hypothesize that Axi-cel will improve the outcome of patients with DLBCL who are refractory or relapse early (i.e. within 1 year from end of treatment) after first-line therapy and who are not eligible for ASCT. The primary endpoint will be

	complete metabolic response (CMR) at 3 months after Axi-cel infusion. This timepoint may be compared to the end of treatment evaluation after 8 cycles of R-GEMOX. Indeed, R-GEMOX lasts for approximately 4 months (8 x 2 weeks). The 3 months evaluation after Axi-cel will correspond approximately to month 4 after enrollment (1 month before Axi-cel infusion (leukapheresis + CAR-T manufacturing) + 3 months after infusion).<br>Transplant-ineligible patients will include those who are deemed ineligible for high-dose chemotherapy and HSCT due to age, comorbidity, or prior ASCT. For this purpose, the hematopoietic cell transplantation-comorbidity index (HCT-CI) has been established to estimate morbidity and treatment-related mortality after allogeneic stem cell transplantation. It encompasses rather easily dysfunctions of distinct organ systems. Recently, the HCT-CI score has been validated in a large prospective study also for recipients of autologous stem cell transplantation. Patients with HCT-CI scores ≥ 3 experienced a significantly higher non-relapse mortality and inferior OS. Thus, in the current study, patients will be considered ASCT ineligible if they are 65 years or older, if their HCT-CI score is ≥ 3 regardless of age, or if they underwent prior consolidation with ASCT during first line therapy.<br>Patients with HCT-CI score ≥ 3, although not eligible for ASCT, may remain eligible to CAR-T cell therapy after evaluation by a CAR-T expert and if they meet all the pre-defined criteria described in the protocol. These criteria have been determined based on prior clinical trials and real-world experience which showed that CAR T-cell therapy is feasible and safed in such patients.
**Study objectives and endpoints**	**Primary objective**:<br>To evaluate the complete metabolic response (CMR) at 3 months from Axi-cel infusion (without additional anticancer therapy) based on investigator disease assessment<br><br>**Primary endpoint**: CMR at 3 months based on investigator disease assessment according to PET-scan using the Lugano Response Criteria<br><br>**Secondary objectives**:<br>To evaluate efficacy and safety of Axi-cel infusion (without additional anticancer therapy) with respect to secondary efficacy endpoints.<br><br>**Secondary endpoints:**<br>• CMR at 3 months from Axi-cel infusion (without additional anticancer therapy) determined by central imaging review (using the Lugano Response Criteria)<br>• Event-free survival (EFS) at 3, 6 and 12 months from leukapheresis based on investigator disease assessment. EFS is defined as the time between leukapheresis and:<br>o any event preventing Axi-cel infusion if Axi-cel is never infused, or<br>o death, disease progression, or instauration of a new lymphoma therapy for lymphoma progression after Axi-cel infusion.<br>• EFS at 3, 6 and 12 months from leukapheresis based on central imaging review. EFS is defined as the time between leukapheresis and:<br>o any event preventing Axi-cel infusion if Axi-cel is never infused, or<br>o death, disease progression, or instauration of a new lymphoma therapy for lymphoma progression after Axi-cel infusion.<br>• Modified EFS (mEFS) at 6 and 12 months from leukapheresis based on investigator assessment and central imaging review. mEFS is defined as the time between leukapheresis and:<br>o any event preventing Axi-cel infusion if Axi-cel is never infused, or<br>o death, disease progression, or instauration of a new lymphoma therapy for lymphoma progression after Axi-cel infusion or failure to achieve a CMR at 6 and 12 months post-CAR infusion.<br>• Best objective response (complete and partial metabolic response)<br>• Duration of response (DOR)<br>• Progression-free survival (PFS) from Axi-cel infusion<br>• Overall survival (OS) from leukaphaeresis and Axi-cel infusion<br>• DOR, PFS, OS at 2 years and 3 years

	• Safety of Axi-cel<br>• Health-related Quality of life (EORTC QLQ-C30, EQ-5D-5L and QLQ-NHL-HG29)<br><br>**Exploratory objectives**:<br>To evaluate the mechanisms of action of Axi-cel (without additional anticancer therapy) with respect to efficacy and toxicity, in relation with:<br>• Baseline metabolic tumor volume and early metabolic response<br>• Characteristics of the tumor, the microenvironment, and the host<br>• Pre and post-treatment blood biomarkers including inflammation markers and PK/PD profiling<br>• Product characteristics and cellular kinetics *in vivo*<br>• Host immune response (including epitope spreading) and mechanisms of resistance<br><br>**Exploratory endpoints:**<br>• <u>Imaging</u>:<br>- Correlation between Total Metabolic Tumor Volume (TMTV) pre-Axi-cel infusion and efficacy/toxicity<br>- Correlation between early metabolic response (Day 14) and efficacy/toxicity<br><br>• <u>Biology</u>:<br>Biological exploratory endpoints will be described in relation with efficacy and toxicity and reported separately. They will be based on correlation with biological parameters such as:<br>- Histologic, phenotypic, genomic, transcriptomic, and molecular characteristics of malignant cells and tumor microenvironment pre and post treatment<br>- Cell product characteristics (*in vitro* proliferative potential, TCR repertoire, immunophenotype…) and cellular kinetics *in vivo* (such as peak concentration and persistence)<br>- Immune response markers in tumor and blood such as cytokine levels, immune cells, and TCR repertoire.<br>- cfDNA concentration and mutational patterns evolution in plasma<br>- Immune-escape mechanisms including resistance of tumor cells to T-cell killing and tumor-induced immune suppression.
**Study design**	This is a phase 2, open-label, multicenter study evaluating Axi-cel as a 2nd line therapy in patients with Relapsed/Refractory aggressive B-NHL who are ineligible to receive Autologous Stem Cell Transplantation but eligible to receive CAR T-cell therapy.<br><br>40 patients infused with Axi-cel will be enrolled in France and Belgium for the main criterion analysis.<br>20 additional patients infused with Axi-cel will be enrolled in France and Belgium.<br><br>Patients with relapse/refractory disease after 1st line R-CHOP or R-CHOP-like chemotherapy and deemed to be ineligible to autologous stem cell transplantation will be screened to receive axi-cel infusion. Following a multidisciplinary meeting, patient's eligibility for this study will be discussed.<br>Patients will undergo leukapheresis within 15 days post screening (ICF signature). If needed, patients are allowed to receive corticosteroids between screening and leukapheresis but corticosteroids (per os or IV route) must be discontinued at least 7 days prior to leukapheresis. After leukapheresis, patients may receive a bridging therapy with 1 or 2 cycles of R-GEMOX but the last infusion must be completed at least 14 days prior to the start of conditioning chemotherapy. Patients may also receive corticosteroids as a bridging therapy (type and dose at the discretion of the investigator) but administration of corticosteroids must be discontinued at least 2 days before pre-treatment PET-CT.<br>A pre-treatment PET-CT will be performed within 7 days before the start of conditioning chemotherapy.<br>When CAR T-cells are delivered to the sites, patients will receive 3 days of conditioning chemotherapy regimen consisting of fludarabine 30 mg/m²/day (for patients with creatinin clearance between 40 and 60 mL/min, fludarabine will be

	reduced to 25 mg/m^2/day, cyclophosphamide will remain unchanged at 500 mg/m^2/day) and cyclophosphamide 500 mg/m^2/day followed by 2 rest days minimum and a maximum of 7 days before Axi-cel infusion. If infusion cannot be done within 7 days after the conditioning chemotherapy regimen, this will be discussed with Coordinating investigators on a case by case basis.<br>A single infusion of Axi-cel will then be administered intravenously at a target dose of 2 x 10^6 anti-CD19 CAR T cells/kg.<br>Patients will be evaluated (PET-CT) at Day 14 and Month 1, 3, 6, 9 and 12 after Axi-cel infusion (day 0).<br>After that, patients will be followed annually at 2 and 3 years.<br>After 3 years, a long-term follow-up until 15 years will be done via DESCAR-T, French register of patients with malignant hemopathies and eligible for car-t cell therapy.

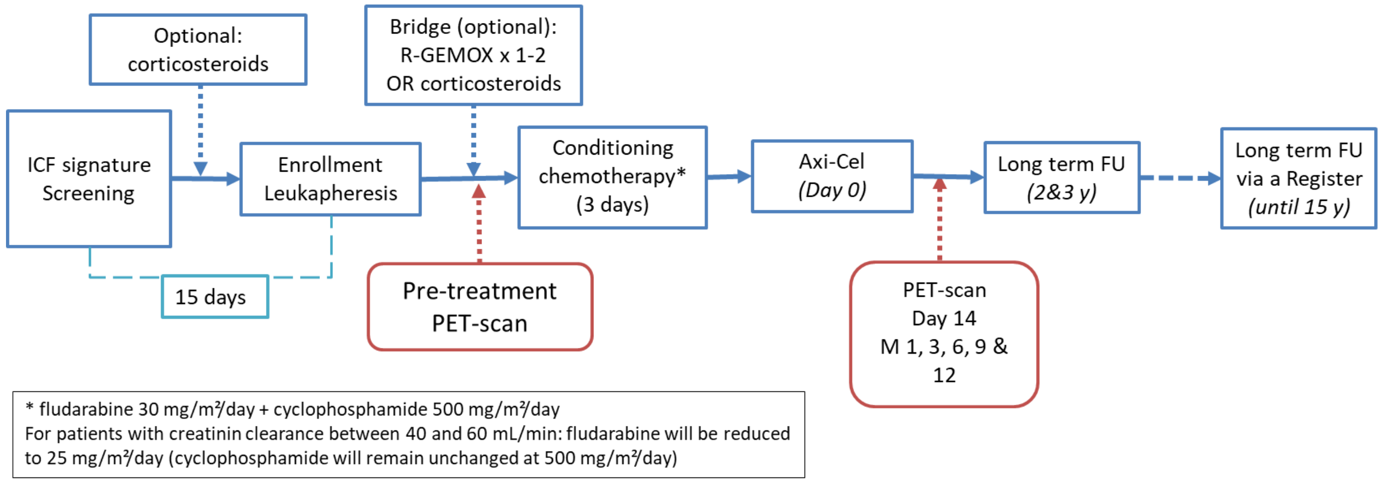

**Duration of the study**	Patients will be enrolled for 14 months, treated with a single infusion of Axi-cel and followed up to 3 years after last patient treated with Axi-cel. The total duration of the study is therefore 5 years.<br>Theoretical dates of studies:<br>- 1st patient included (FPFV): 01/12/2020<br>- Last patient included (LPFV): 28/02/2022<br>- End of study: 01/06/2025<br>The end of study is the 3 years of follow-up of the last patient treated.
**Number of patients**	40 patients having received Axi-Cel infusion and for 20 additional patients having received Axi-Cel infusion, for subgroup analysis.
**Inclusion criteria**	1. Signed written Informed Consent Form<br>2. Patient who understands and speaks one of the country official languages<br>3. Histologically proven relapsed or refractory aggressive B-cell non-Hodgkin lymphoma (B-NHL) of the following histology at relapse: diffuse large B-cell lymphoma (DLBCL), high-grade B-cell lymphoma (HGBL), follicular lymphoma Grade 3B per WHO 2016 classification and Primary mediastinal B-cell lymphomas. Indolent B-NHL who transformed into aggressive B-NHL and were previously treated with R-CHOP are eligible.<br>4. Tumoral tissue (at diagnosis or relapse) available for central pathology review, exploratory endpoints and ancillary studies<br>5. Positron-emission tomography (PET)-positive disease<br>6. Patients must have received adequate first-line therapy including at a minimum:<br>o An anti-CD20 monoclonal antibody (rituximab or obinutuzumab), and

	o CHOP or CHOP-like chemotherapy<br>**Note**: CHOP-like chemotherapy corresponds to ACVBP or EPOCH or COPADEM. Dose-reduced CHOP (i.e. miniCHOP) is excluded except for dose-reductions of vincristin due to peripheral neuropathy. Patients who have received additional drugs in combination with CHOP or CHOP-like regimen are eligible.<br>7. Relapsed or refractory disease after first-line chemoimmunotherapy (full dose of R-CHOP or R-CHOP-like regimen), documented by PET-scan:<br>o Relapsed disease defined as complete remission to first-line therapy followed by biopsy proven disease relapse within 12 months from end of first-line therapy.<br>Patients who received first line of R-CHOP or obinutuzumab-CHOP for an indolent B-NHL who relapse as transformed aggressive B-NHL within a year from the end of first-line therapy are eligible.<br>o Refractory disease defined as:<br>✓ Progressive disease (PD) during first-line therapy<br>✓ Stable disease (SD) as best response after at least 4 cycles of first-line therapy (e.g. 4 cycles of R-CHOP)<br>✓ Partial response (PR) as best response after at least 6 cycles, and biopsy-proven residual disease<br>8. At least 2 weeks must have elapsed since any prior systemic cancer therapy at the time the patient provides consent<br>9. Patients must be autologous stem cell transplantation (ASCT)-ineligible as defined by:<br>o Patient deemed ineligible for high-dose chemotherapy and ASCT based on physician's assessment<br>o AND at least one of the following criteria:<br>✓ Age ≥ 65 years or<br>✓ Age ≥ 18 years and Hematopoietic Cell Transplantation-specific Comorbidity Index (HCT-CI – Appendix 09) score ≥3 or<br>✓ Age ≥ 18 years and prior ASCT (as 1st line consolidation)<br>10. Patients must meet CAR-T-eligible as defined by:<br>o Patient deemed eligible for CAR T-cells therapy by the CAR-T physician<br>o AND all the following criteria:<br>✓ ECOG performance status of 0, 1 or 2<br>✓ Adequate vascular access for leukapheresis procedure (either peripheral or central venous line)<br>✓ Absolute neutrophil count (ANC) ≥ 1 G/L<br>✓ Platelets ≥ 75 G/L<br>✓ Absolute lymphocyte count ≥ 0,1 G/L<br>✓ Creatinine clearance (as estimated by Cockcroft Gault or MDRD) ≥ 40 mL/min<br>✓ Serum alanine aminotransferase/aspartate aminotransferase (ALT/AST) ≤ 2.5xULN<br>✓ Total bilirubin ≤ 26 µmol/L, except in patients with Gilbert's syndrome<br>✓ Cardiac ejection fraction ≥ 45%<br>✓ Baseline oxygen saturation ≥ 92% on room air<br>11. Females of childbearing potential must have a negative serum or urine pregnancy test (females who have undergone surgical sterilization or who have

	been postmenopausal for at least 12 months are not considered to be of childbearing potential)
**Exclusion criteria**	1. Patients who received more than one prior line of systemic therapy<br>2. Patients who are intolerant to first-line therapy or who received suboptimal first-line therapy, including dose-reduced R-CHOP ("R-miniCHOP"), and those who discontinued prematurely first-line therapy due to toxicity are not eligible (except for dose-reductions or discontinuation of vincristin due to peripheral neuropathy)<br>3. Prior CD19 targeted therapy<br>4. Patients with cardiac atrial or cardiac ventricular lymphoma involvement<br>5. Requirement for urgent therapy due to tumor mass effects, such as bowel obstruction or blood vessel compression<br>6. Patient with clinically significant pleural effusion<br>7. History of another primary malignancy that has not been in remission for at least 2 years (except for nonmelanoma skin cancer or carcinoma in situ (eg, cervix, bladder, breast)). A maintenance treatment is not allowed.<br>8. Patients with detectable Central Nervous System (CNS) lymphoma. Patients with a history of CNS lymphoma but no active CNS disease (after systematic MRI and lumbar puncture) at the time of enrollment will be eligible.<br>9. History or presence of non-malignant CNS disorder, such as seizure disorder requiring anti-convulsive therapy, cerebellar disease, or any autoimmune disease with CNS involvement disease<br>10. Active hepatitis B or hepatitis C infection at the time of screening<br>Active Hepatitis B Virus (HBV) infection defined as:<br>- HBs Ag positive<br>- HBs Ag negative, anti-HBs antibody positive and/or anti-HBc antibody positive with detectable viral DNA<br>11. Positive serology of human immunodeficiency virus (HIV) and syphilis at the time of screening<br>12. Uncontrolled systemic fungal, bacterial, viral or other infection despite appropriate antibiotics or other treatment at the time of leukapheresis or Axi-cel administration<br>13. History of any one of the following cardiovascular conditions within the past 6 months: Class III or IV heart failure as defined by the New York Heart Association, cardiac angioplasty or stenting, myocardial infarction, unstable angina, or other clinically significant cardiac disease<br>14. History of autoimmune disease requiring systemic immunosuppression and/or systemic disease modifying agents within the last year<br>15. History of idiopathic pulmonary fibrosis, organizing pneumonia (eg, bronchiolitis obliterans), drug-induced pneumonitis, idiopathic pneumonitis, or evidence of active pneumonitis per chest computed tomography (CT) scan at screening. History of radiation pneumonitis in the radiation field (fibrosis) is allowed.<br>16. History of severe immediate hypersensitivity reaction to tocilizumab or any of the agents used in this study<br>17. History of severe immediate hypersensitivity reaction attributed to aminoglycosides, cyclophosphamide and fludarabine

	18. Treatment with a live, attenuated vaccine within 6 weeks prior to initiation of study treatment or anticipation of need for such a vaccine during the course of the study<br>19. Women of childbearing potential who are pregnant or breastfeeding because of the potentially dangerous effects of chemotherapy on the fetus or infant. Patients of either sex who are not willing to practice birth control from the time of consent during treatment and for at least 6 months after conditioning chemotherapy dosing or axicabtagene ciloleucel dosing, whichever is later<br>20. In the investigator's judgment, the patient is unlikely to complete all protocol-required study visits or procedures, including follow-up visits, or comply with the study requirements for participation<br>21. Adult person unabled to provide informed consent because of intellectual impairment, any serious medical condition, laboratory abnormality or psychiatric illness.
**Study treatment**	Axicabtagene ciloleucel<br>A single infusion of axicabtagene ciloleucel will be administered intravenously at a target dose of 2 x 10^6 anti-CD19 CAR T cells/kg.
**Assessment schedule**	Clinical examinations (including ECOG performance status), laboratory safety tests (including complete blood counts, serum chemistries and serologies), tumor biopsy (if no tumoral tissue is available from diagnosis or relapse), cerebral MRI and quality of life (QoL) assessments will be obtained within 15 days prior to enrollment.<br>Relevant medical history will be collected before inclusion.<br>A pregnancy test will be performed within 15 days prior enrollment for all females with childbearing potential.<br>A neurological consultation will be performed prior to Axi-cel infusion.<br>Clinical examination, laboratory tests and blood sampling will be performed at baseline (before leukapheresis), at Day 0 of Axi-Cel infusion, daily during hospitalization (D1-D10), at day 14, and months 1, 3, 6, 9 and 12.<br>PET-CT scans will be performed within 7 days before the start of prior conditioning chemotherapy, at day 14, months 1, 3, 6, 9 and 12 post-Axi-Cel infusion.<br>To ensure comparability, prior treatment and on-study methods for response assessment will be performed using identical techniques.<br>Quality of life questionnaires will be administered the first day of conditioning chemotherapy administration and month 1, 3, 6 and 12. A long-term follow-up will be performed at 2 and 3 years. Survival status and new lymphoma therapy for lymphoma progression will be collected.
**Safety considerations - Pharmacovigilance**	All AEs of grade ≥ 2, regardless of the relationship to treatment, occurring after leukapheresis procedure to 30 days after Axi-Cel infusion will be recorded in the AE pages of the eCRF.<br>The following AEs are considered as of special interest and require attention from investigator if occurring: Cytokine Release Syndrome (CRS), neurotoxicities, prolonged/delayed cytopenias (beyond 1 month) and hypogammaglobulinemia from Grade 1, documented infections and emergent secondary malignancy. It has to be reported immediately in the eCRF until the end of follow-up, irrespective of seriousness criteria.<br>No Independent Data Monitoring Committee will be consulted.
**Screening in the study and Enrollement**	After ICF signature, patients will be screened and registered directly in the data entry system, via Internet at the following address: https://lysarc.ennov.com/EnnovClinical/login?etude=ALYCANTE<br><br>To access the interactive registration program, the investigator must enter the name of the study (ALYCANTE), his username and password.

Statistical consideration	**SAMPLE SIZE CALCULATION**<br>The primary endpoint in the study is the complete metabolic response at 3 months from Axi-cel infusion based on investigator disease assessment according to PET-scan review (Lugano criteria).<br>Sample size calculation was performed with EAST 6.5 using an exact single-stage phase II design (Jennison, C. and Turnbull, B.W. (2000). Group Sequential Methods with Applications to Clinical Trials. Chapman & Hall pp 235-244).<br>No interim analysis is planned.<br><br>*Hypothesis*<br>It is hypothesized that the 3 months-CMR is 12% with SOC (based on historical controls) and 34% with CAR-T cells.<br>The hypothesis are as follows:<br>- improvement of the CMR at 3 months from 12% to 34%<br>- one-sided alpha: 5%<br>- power: 96%<br>- enrollment duration: 14 months<br>- study duration: 5 years<br>Based on these hypotheses, 40 patients with infusion of Axi-Cel are needed.<br><br>*Sample Size*<br>Patients having signed their informed consent and with a confirmed enrollment will be included in this study.<br>It is necessary to ensure these assumptions are similar between patients who are over and under the age of 70.<br>It is hypothesized that distribution will be equivalent in both age subgroups.<br>In order to have a sufficient power in each subgroup, a minimum of 25 patients is required (power of 80%) and 30 patients are expected (power of 85%). Thirty (30) evaluable patients in each subgroup will allow to have a sufficient power and to prevent possible unbalanced age distribution.<br>This will require the addition of 20 evaluable patients. Assuming a 10% of drop-out, 22 additionnal patients need to be enrolled.<br>Enrollment will be stopped once 60 patients have been infused with Axi-Cel (mFAS).<br><br>**ANALYSIS POPULATION**<br>**Enrolled set (ES):** will include all patients having signed their informed consent.<br><br>**Full Analysis Set (FAS):** will include all patients having signed their informed consent and with a confirmed enrollment.<br><br>**modified Full Analysis Set (mFAS):** will include all patients having signed their informed consent and who have been infused with Axi-Cel<br><br>**The Safety Set (SS):** will include all patients included in the mFAS.<br><br>**Age subgroup:** will be set up to split patients under the age of 70 (strictly) from others.<br><br>**ECOG subgroup**: will be set up to split patients with ECOG 0-1 from others (only if percent of patients with ECOG≥2 is greater than 10%)<br><br>**STATISTICAL ANALYSIS PLAN**<br>Survival endpoints will be performed using Kaplan-Meier methodology. Survival probabilities, median survival and quartiles will be estimated (if reached) with their 95% CI. Survival curves will be provided.<br>Response rates will be expressed with 90% confidence limits (to be consistent with one-sided 5% level of significance) according to Pearson-Clopper method. Missing response will be considered as Not evaluated and therefore, as Non-responder. The number and percent of patients falling into each category of response (CMR, PMR, NMR, PMD, NMR, PD, Not evaluated) will be provided.<br>Secondary safety endpoints including AEs will also be described.

ANALYSES

Interim analysis

No interim analysis planned for CMR (primary endpoint).

Final analysis

<table>
<tr><th>Calendar of analysis</th><th>mFAS - 40 patients
Cut-off</th><th>mFAS – 62 patients
Cut-off</th></tr>
<tr><td>Main criterion analysis</td><td>3 months after 40th infused patients</td><td rowspan="2">6 months after 62 infused patients*</td></tr>
<tr><td>Secondary endpoints analysis</td><td>6 months after 62 infused patients*</td></tr>
<tr><td>Update of the secondary endpoints analysis</td><td colspan="2">12 months after 62 infused patients</td></tr>
<tr><td rowspan="2">Update of the survival endpoints analysis</td><td colspan="2">24 months after 62 infused patients*</td></tr>
<tr><td colspan="2">36 months after 62 infused patients**</td></tr>
</table>

* to avoid multiple analysis
** combined with end of study analysis

- Main criterion analysis

The final CMR analysis will be performed when the targeted patients (40 then 62 for subgroup analyses) having received Axi-cel infusion have performed the investigator disease assessment at 3 months from Axi-cel infusion.

- Secondary endpoints analysis (CMR rate by IRC, EFS, Best objective response, DOR, PFS, OS)

It will occur when all patients having received Axi-cel infusion have performed the investigator disease assessment at 6 months from Axi-cel infusion, have died, have withdrawn consent, or are lost to follow-up, whichever occurs first.
The CMR according to the investigator assessment will be analyzed on the mFAS by age subgroup on the 60 evaluable patients. Secondary endpoints will also be analyzed by age subgroup for exploratory purpose.

- Update of the secondary endpoints analysis

It will occur when all patients having received Axi-cel infusion have performed the investigator disease assessment at 12 months from Axi-cel infusion, have died, have withdrawn consent, or are lost to follow-up, whichever occurs first.

- Update of the survival endpoints analysis

It will occur when all patients having received Axi-cel infusion have performed the investigator disease assessment at 24 months and 36 months from Axi-cel infusion, have died, have withdrawn consent, or are lost to follow-up, whichever occurs first.

- End of study analysis

Update of the survival endpoints will be performed at the end of study when all patients having received Axi-cel infusion have completed follow-up, have died, have withdrawn consent, or are lost to follow-up, whichever occurs first.

2 TABLE OF CONTENTS

1 SYNOPSIS 3

2 TABLE OF CONTENTS 12

3 LIST OF ABBREVIATIONS AND GLOSSARY OF TERMS 16

4 RESPONSIBILITIES....18

4.1 Sponsor and program coordination center 18

4.1.1 Sponsor....18

4.1.2 Coordinating investigators 18

4.1.3 Biology, anatomopathology and imaging referents 18

4.1.4 Program coordination center 19

4.2 Investigators....19

4.3 Laboratory sites 20

5 BACKGROUND AND STUDY RATIONALE....21

5.1 Axicabtagene Ciloleucel 21

6 STUDY OBJECTIVES 23

6.1 Primary objective 23

6.2 Secondary objectives 23

6.3 Exploratory objectives 23

7 STUDY DESIGN 25

8 STUDY POPULATION 26

8.1 Inclusion criteria 26

8.2 Exclusion criteria 27

9 STUDY FLOW CHART AND SCHEDULE OF ASSESSMENTS....29

9.1 Study flow chart 29

9.2 Schedule of assessments 29

9.3 Informed consent....29

9.4 Baseline assessments 29

9.5 Enrollment and Leukapheresis 30

9.6 Pre-treatment PET scan 31

9.7 Conditioning Chemotherapy Administration (Day - 5 through Day - 3 before infusion of axi-cel) ..31

9.8 Assessments during treatment 33

9.8.1 Requirements for Axicabtagene Ciloleucel Infusion 33

9.8.2 Monitoring After Axicabtagene Ciloleucel Infusion 34

9.9 End of treatment and permanent study discontinuation assessments 35

9.10 Follow-up assessments (between 3 months – 1 year)....35

9.10.1 Patients who have completed treatment or discontinued treatment due to reasons other than progressive disease or relapse (meaning patients who did not experienced progression or relapse) 35

9.10.2 Patients who experienced progressive disease or relapse 36

9.11 Long term follow-up period assessments (≥ 1year)....36

9.12 Progression/relapse 36

10 TREATMENTS 37

10.1 Investigational Medicinal Product description, storage and handling 37

10.1.1 Description 37

10.1.2 Packaging and labeling....37

10.1.3 Storage conditions 37
10.1.4 Handling 37
10.2 Treatment schedule and design 38
10.2.1 Bridging Therapy (administered after leukapheresis) – optional 38
10.2.2 Conditioning chemotherapy 38
10.2.3 Axicabtagene Ciloleucel 38
10.3 Concomitant treatment 38
10.3.1 Prohibited therapies 39
10.3.2 Restricted/allowed therapies 39
10.4 Drug Dispensation 39
10.4.1 Responsibilities 39
10.4.2 Retrieval or destruction 39
10.4.3 Accountability and compliance 40
10.5 Prophylactic measures 40

11 STUDY PROCEDURES 41
11.1 Screening and enrollment procedure 41
11.2 Pathological diagnosis 41
11.2.1 Patient enrollment 41
11.2.2 Sample request 41
11.2.3 Sample centralisation at LYSA-P 42
11.2.4 Sample review 42
11.3 Biological banking studies 42
11.3.1 Informed consents 43
11.3.2 Biological banking and studies 43
11.3.3 Process 44
11.4 PET scan Review 46
11.5 Quality of life questionnaires 46

12 STUDY COMMITTEES 48
12.1 Centralized Review Committee (CRC) 48

13 CRITERIA FOR PERMANENT TREATMENT DISCONTINUATION OF THE STUDY 49
13.1 Permanent treatment discontinuation 49
13.2 Withdrawal of Consent 49
13.3 Patients Lost to Follow up 49
13.4 Discontinuation of the study 49

14 SAFETY PARAMETERS 50
14.1 Definitions 50
14.1.1 Adverse Events 50
14.1.2 Serious Adverse Events 50
14.1.3 Intensity 50
14.2 Adverse Events reporting rules 50
14.3 Serious Adverse Events reporting rules 52
14.3.1 Obligations of the investigator 52
14.3.2 Obligations of the Sponsor 53
14.4 Follow up of AEs and SAEs 53
14.5 Adverse Events of Special Interest 54
14.6 Pregnancy 55
14.6.1 Females of Childbearing Potential 55
14.6.2 Male patients 56

15 GENERAL STATISTICAL CONSIDERATIONS 57
15.1 Primary endpoint 57
15.2 Secondary efficacy endpoints 57
15.3 Secondary safety endpoints 59
15.4 Exploratory endpoints 59
15.5 Analysis sets 59
15.5.1 Enrolled Set (ES) 59
15.5.2 Full Analysis Set (FAS) 59
15.5.3 Modified Full Analysis Set (mFAS) 59
15.5.4 Safety Set (SS) 60
15.5.5 QoL Set 60
15.6 Statistical methods 60
15.6.1 Patients replacement 60
15.6.2 General Approach 60
15.6.3 Efficacy Analysis 60
15.6.4 Safety Analysis 61
15.7 Sample size 60
15.8 Analyses 61
15.8.1 Interim analysis 61
15.8.2 Final analysis 61
16 STUDY MONITORING 62
16.1 Responsibilities of investigators 62
16.2 Responsibilities of the sponsor 62
16.3 Source document requirements 62
16.4 Use and completion of electronic case report form (eCRF) 62
17 ETHICAL AND REGULATORY STANDARDS 63
17.1 Ethical principles 63
17.2 Laws and regulations 63
17.3 Informed consent 63
17.4 Ethics Review Committee and Competent Authorities submission 63
18 ADMINISTRATIVE PROCEDURES 64
18.1 Curriculum vitae 64
18.2 Confidentiality agreement 64
18.3 Record retention in investigating sites 64
18.4 Data Collection 64
18.5 Ownership of data and use of the study results 64
18.6 Publication 65
18.7 Insurance compensation 65
18.8 Company audits and inspections by regulatory agencies 65
18.9 Clinical study report 65
18.10 Protocol amendments 66
19 REFERENCES 67
20 APPENDICES 69
20.1 Appendix 01: Study Design 69
20.2 Appendix 02: Schedule of Assessments (study flow-chart) 70
20.3 Appendix 03: Schedule of biological banking samples 73
20.4 Appendix 04: Body Surface Area calculation 74

20.5 Appendix 05: Performance Status Criteria 75
20.6 Appendix 06: Ann Arbor staging....76
20.1 Appendix 07: International Prognostic Index (IPI) 77
20.2 Appendix 08: Quality of life questionnaires....78

NOT AT A QUITE VERY 79

ALL LITTLE A BIT MUCH....79

DURING THE PAST WEEK: NOT AT A QUITE VERY 79

ALL LITTLE A BIT MUCH....79

DURING THE PAST WEEK: NOT AT A QUITE VERY 80

ALL LITTLE A BIT MUCH....80
20.3 Appendix 09: Hematopoietic Cell Transplantation-specific Comorbidity Index (HCT-CI) 85
20.4 Appendix 10: Response Criteria for Lymphoma – Lugano Classification....87
20.5 Appendix 11: Deauville criteria for PET analysis 89
20.6 Appendix 12: PET SCANs 90
20.6.1 Timing of FDG PET scans 90
20.6.2 Patient preparation....90
20.6.3 PET scanner technical requirements....91
20.6.4 PET acquisition and reconstruction 91
20.7 Appendix 13: Acquisition protocol for PET_D14....92
20.8 Appendix 14: ASTCT CRS Grading....93
20.9 Appendix 15 - CRS Grading Scale (Excluding Neurologic Events) per Lee, 2014....94
20.10 Appendix 16 – Management of CRS (based on Lee criteria)....95
20.11 Appendix 17 -- ASTCT Consensus Grading for Neurologic Events 97
20.12 Appendix 18 - Management of Neurologic Events (based on CTCAE grading)....99

3 LIST OF ABBREVIATIONS AND GLOSSARY OF TERMS

Abbreviation	Term
AE	Adverse Event
ALC	Absolute Lymphocyte Count
ALT (SGPT)	ALanine Transaminase (Serum Glutamic Pyruvic Transaminase)
ANC	Absolute Neutrophil Count
ASBMT	American Society for Blood and Marrow Transplantation
ASCO	American Society of Clinical Oncology
ASCT	Autologous Stem Cell Transplantation
ASH	American Society of Hematology
AST (SGOT)	ASpartate Transaminase (Serum Glutamic Oxaloacetic Transaminase)
βHCG	beta-Human Chorionic Gonadotropin
BSA	Body Surface Area
CA	Competent Authorities
CAR	Chimeric Antigen Receptor
CBC	Complete Blood Cell Count
CD20	antigen expressed on the surface of normal and malignant B lymphocytes
CFR	Code of Federal Regulations
CHOP	cyclophosphamide, doxorubicin, vincristine, and prednison
CIBMTR	Center for International Blood and Marrow Transplant Research
CMR	Complete Metabolic Response
CR	Complete Response
CRF	Case Report Form
CRR	Complete Response Rate
CRS	Cytokine Release Syndrome
CSF	Cerebrospinal fluid
CT	Computed Tomography
CTCAE	Common Terminology Criteria for Adverse Events
DLBCL	Diffuse Large cell B-Cell Lymphoma
DSUR	Development Safety Update Report
DOR	Duration Of Response
EC	Ethics Committee
ECG	Electrocardiogram
ECHO	Echocardiogram
ECOG	Eastern Cooperative Oncology Group
eCRF	Electronic Case Report Form
EFS	Event Free Survival
EOR	End of treatment Response
FCBP	Female of Childbearing Potential
FDG	FluoroDeoxyGlucose (18^F)
GCP	Good Clinical Practice
G-CSF	Granulocyte Colony-Stimulating Factor
HBV	Hepatitis B Virus
HCV	Hepatitis C Virus
HIV	Human Immunodeficiency Virus
HSCT	Hematopoietic stem cell transplantation
ICANS	Immune effector cell-associated neurotoxicity syndrome
ICH	International Conference on Harmonization

IP	Investigational Product
IRC	Independent Review Committee
IRR	Infusion Related Reaction
IV	IntraVenous
LDH	Lactate DeHydrogenase
LYSA	The Lymphoma Study Association
LYSARC	The Lymphoma Academic Research Organisation
MDRD	Modification of Diet in Renal Disease
mEFS	Modified EFS
MRI	Magnetic Resonance Imaging
NHL	Non-Hodgkin's Lymphoma
NMR	No Metabolic Response
ORR	Overall Response Rate
OS	Overall Survival
PD	Progressive Disease
PET	18F-FDG Positon Emission Tomography
PFS	Progression Free Survival
PK	PharmacoKinetics
PMR	Partial Metabolic Response
PR	Partial Response
PS	Performance Status
RD	Recommended Dose
RPPS	Répertoire Partagé des Professionnels de Santé *(Health Professionals Shared Directory)*
RR	Response Rate
SAE	Serious Adverse Event
SD	Stable Disease
SPM	Second Primary Malignancy
SUSAR	Suspected Unexpected Serious Adverse Reaction
SUVmax	Maximum Standardized Uptake Value
TMA	Tissue Micro Array
ULN	Upper Limit of Normal
US	United States
WBC	White Blood Cell
WHO	World Health Organization

4 RESPONSIBILITIES

4.1 Sponsor and program coordination center

4.1.1 Sponsor

LYSARC (the Lymphoma Academic Research Organisation)
🖂: Centre Hospitalier Lyon Sud – Bâtiment 2D
69495 Pierre Bénite Cedex - France
☎: +33(0) 4 72 66 93 33
Fax: +33(0) 4 26 07 40 55
Email: alycante@lysarc.org

4.1.2 Coordinating investigators

France

Prof Roch HOUOT
🖂: CHU de Rennes - Hôpital Pontchaillou - 2 rue Henri Le Guilloux - 35033 Rennes Cedex - France
☎: +33 (0)2 99 28 42 26
Email: Roch.houot@chu-rennes.fr

Prof François LEMONNIER
🖂: Hôpital Henri Mondor – Lymphoid Malignancies Unit –51, avenue du Maréchal de Lattre de Tassigny – 94000 Créteil - France
☎: +33 (0)1 49 81 43 20
Email: francois.lemonnier@aphp.fr

Belgium

Dr Christophe BONNET
🖂: CHU de Liège – Hematology - Domaine du Sart Tilman, Bât B35 - B-4000 Liège - Belgium
☎: + 32 43 66 84 20
Email: christophe.bonnet@chuliege.be

4.1.3 Biology, anatomopathology and imaging referents

Biology referents

Prof Karin Tarte
🖂: Laboratoire de Suivi Immunologique des Thérapies Innovantes, Bâtiment Jean Dausset, CHU Pontchaillou, 2 rue Henri Le Guilloux, 35033 Rennes - France
☎: +33(0) 2 99 28 37 75
Email: karin.tarte@univ-rennes1.fr

Prof Marie-Hélène Delfau-Larue
🖂: INSERM U955 équipe 9, Laboratoire d'immunologie, Hôpital Henri-Mondor, 94000 Creteil - France
☎: +33(0)1 49 81 26 65
Email: marie-helene.delfau@aphp.fr

Anatomopathology referents

Prof Camille Laurent
🖂: Institut Universitaire du Cancer de Toulouse, Département de Pathologie 1 avenue Irène Joliot-Curie 31059 Toulouse - France
☎: +33(0)5 31 15 61 99

Email: laurent.c@chu-toulouse.fr

Dr Francisco Llamas Gutierrez
⌧: Centre Hospitalier Universitaire de Rennes, Département de Pathologie, 2 Rue Henri le Guilloux. 35000 Rennes - France
☎: +33(0)2 99 28 42 79
Email: Francisco.llamas.gutierrez@chu-rennes.fr

Imaging referents

Prof Emmanuel Itti
⌧: CHU Henri Mondor – Service de Médecine Nucléaire – 51 Av du Mal de Lattre de Tassigny - 94010 Créteil - France
☎: +33(0)1 49 81 27 80
Email: emmanuel.itti@aphp.fr

Dr Clément Bailly
⌧: CHU de Nantes, Hôtel Dieu – Service de Médecine Nucléaire, 1 place Alexis Ricordeau, 44000 Nantes - France
☎: +33(0) 2 40 08 41 36
Email: clement.bailly@chu-nantes.fr

4.1.4 Program coordination center

Project Management

⌧: LYSARC - CHU Lyon Sud - Bâtiment 2D - 69495 PIERRE BENITE Cedex - France
☎: +33(0) 4 72 66 93 33
Fax: +33(0) 4 26 07 40 55
Email: alycante@lysarc.org

Pharmacovigilance

Fax: +33 (0) 3 59 11 01 86
Email: pharmacovigilance@lysarc.org

Biology (LYSA-Bio)

☎: +33(0)4 27 01 27 26
Fax: +33(0)4 26 00 73 33
Email: tous.lysa-bio@lysarc.org

Anatomopathology (LYSA-P) and Imaging (LYSA-IM)

⌧: CHU Henri Mondor - 51, avenue du Maréchal De Lattre de Tassigny - 94010 CRETEIL – France
☎: LYSA-P: +33(0) 1 49 81 37 48 LYSA-IM: +33(0) 1 49 81 47 86
Fax: LYSA-P: +33(0) 1 49 81 37 49
Email: touslysap@lysarc.org Email: imagerie@lysarc.org

4.2 Investigators

Before any enrollment, each participating site must be declared to Ethics Committee and national Competent Authority according to its own country regulations and must have a study training delivered by the Sponsor or its delegate (i.e. initiation visit/call). To be declared as a participating site, the principal investigator must send to

LYSARC all administrative documents required for regulatory submissions (e.g. *curriculum vitae*, medical licence number etc.).

The investigational sites are authorized treatment centers and Investigators are appropriately trained to infuse Axi-cel and to properly monitor patients according to the National Risk Minimisation Measures approved by Competent Authority.

4.3 Laboratory sites

Laboratories of each study site must provide their normal values and an updated accreditation for quality control.

5 BACKGROUND AND STUDY RATIONALE

The addition of rituximab to CHOP chemotherapy (R-CHOP) has improved the outcome of patients with aggressive B-cell lymphoma. However, patients who are relapsing or are refractory after a rituximab containing first-line therapy have a very poor prognosis. Thus, improving outcome of patients with relapsed or refractory aggressive lymphoma represents an unmet medical need.

The population with the highest unmet need consists of patients who do not respond to first line combination chemotherapy (typically R-CHOP). Depending on the number of adverse prognostic factors from the International Prognostic Score (IPI), 20% to 50% of patients with DLBCL will be refractory to R-CHOP or will relapse after achieving complete response (CR). Among patients who progress during initial immunochemotherapy or soon after a brief CR, only 30% to 40% will respond to salvage chemotherapy. In the SCHOLAR-I study, patients (N=179) who where refractory to first line chemotherapy (defined as progressive disease or stable disease as best response after 4 cycles) had a very poor outcome with a median OS of 7.1 months (OS=29% at 1 year) (Crump et al, Blood 2017). In a review of 64 patients with DLBCL with disease progression during first line chemotherapy or only transient response (≤90 days) after end of induction treatment, the response rate to second line therapy was 15%, the median overall survival (OS) was 6 months, and no patient survived more than 26 months after first diagnosis (Josting et al, 2000). In the CORAL trial, patients treated with rituximab-based first-line therapy who relapsed within 1 year of diagnosis had poor outcomes. The 1-year EFS in this group (approximately 60% of the patients) was below 20% in those who relapse before 1 year compared to 50% in those with relapse after 1 year (Gisselbrecht et al, 2010).

Results of the PARMA trial (Philip et al, 1995) demonstrated superior outcome for salvage therapy plus ASCT compared with salvage chemotherapy alone for patients with r/r DLBCL (5-year EFS of 46% vs 12%, and OS of 53% and 32%, respectively), leading to the adoption of second-line chemotherapy plus ASCT as the standard of care. However, the majority of patients are unable to undergo ASCT due to age or comorbidities, and their outcome is poor. Numerous conventionally dosed chemotherapies for relapsed or refractory aggressive lymphoma have been evaluated so far. However, no standard has ever been established by phase-III trials. Rituximab, gemcitabine and oxaliplatin (R-GemOx) is the most common regimen used in this setting. In a pivotal phase-II trial (N=49), R-GemOx induced 44% CRs and 17% PRs, resulting in an ORR of 61%, which is comparable to R-DHAP or R-GDP (Mounier et al, Haematologica 2013). In patients who had relapsed within one year of the prior treatment (N=22), the CR/CRu was 18%. For the entire cohort, the 1-year PFS was 20%, 5-years PFS rate was 13%, while the 5-year OS rate was 14% indicating the poor prognosis of these patients. Among patients who were refractory to first-line therapy or who relapsed within 1 year after achieving a CR (N=17), the PFS was 17.6% at 1 year. In the real-world experience, DLBCL patients who were refractory or who relapsed within 1 year after 1st line treatment with R-CHOP (N=60) had a PFS of 22% at 6 months and 14% at 1 year when treated with 2nd line R-GemOx (Cazelles et al, ASH 2019). Among these patients, only 12% achieved a CR/CRu.

5.1 Axicabtagene Ciloleucel

Axicabtagene ciloleucel (Yescarta®) is a chimeric antigen receptor (CAR) T-cell therapy directed against CD19 which has been approved for the treatment of relapse/refractory DLBCL and primary mediastinal large B-cell lymphoma after 2 or more lines of systemic therapy. In the pivotal ZUMA-1 study, the best ORR was 83% with a complete response (CR) rate of 58%. The CR rate at month 3 post-infusion was 42%. After a median follow-up of 2 years, 37% of patients remained in CR, PFS was 49%, 44% and 39% at 6, 12 and 24 months, respectively; and OS was 78%, 60% and 51% at 6, 12 and 24 months, respectively (Neelapu et al, ASH 2018; Locke et al, Lancet 2019). Interestingly, several analysis suggest that efficacy and safety are similar in elderly patients. In a post-hoc analysis of the ZUMA-

1 trial, including patients ≥ 65 years (N=27) had increased CR (75% vs 53%, respectively), duration of response (median 12 vs 8.1 months, respectively), PFS (median 13.2 vs 5.6 months, respectively), and OS (54% vs 49% at 2 years, respectively) compared to patients <65 years (N=81) (Neelapu et al, Blood 2020). Elderly patients also experienced less severe CRS (7% vs 12% grade≥3, respectively) although they developed more frequently severe neurotoxicity (44% vs 28% grade≥3, respectively).

Additionaly, real-world evidence from the CIBMTR registry demonstrated that the efficacy and safety of Axicabtagene ciloleucel was comparable in DLBCL patients who were ≥ 65 years (N=196) and those who were <65 years (N=337) (Pasquini et al, abst 627, ASH 2019). In patients who were 65 years or older, the PFS at 6 months was ≈ 60%.

Importantly, most patients after the age of 65 cannot tolerate ASCT while many remain eligible to CAR-T cell therapy. Furthermore, administrating CAR T-cells earlier in the therapeutic strategy may be beneficial to patients. Indeed, subset analysis done in the pivotal ZUMA-1 trial demonstrated that DLBCL patients who have received more lines of prior therapy tended to experience lower CAR T-cell expansion, less efficacy and more toxicity compared to those who were treated earlier (Locke et al, Abst S801, EHA 2018).

Based on these findings, we hypothesize that Axi-cel will improve the outcome of patients with DLBCL who are refractory or relapse early (i.e. within 1 year from end of treatment) after first-line therapy and who are not eligible for ASCT. The primary endpoint will be complete response (CR) at 3 months after Axi-cel infusion. This timepoint may be compared to the end of treatment evaluation after 8 cycles of R-GEMOX. Indeed, R-GEMOX lasts for approximately 4 months (8 x 2 weeks). The 3 months evaluation after Axi-cel will correspond approximately to month 4 after enrollment (1 month before Axi-cel infusion (leukapheresis + CAR-T manufacturing) + 3 months after infusion).

Transplant-ineligible patients will include those who are deemed ineligible for high-dose chemotherapy and HSCT due to age, comorbidity, or prior ASCT. For this purpose, the hematopoietic cell transplantation-comorbidity index (HCT-CI) has been established to estimate morbidity and treatment-related mortality after allogeneic stem cell transplantation (Sorror ML et al, Blood 106:2912-9, 2005). It encompasses rather easily dysfunctions of distinct organ systems. Recently, the HCT-CI score has been validated in a large prospective study also for recipients of autologous stem cell transplantation (Sorror ML et al, Biology of Blood and Marrow Transplantation 21:1479-1487, 2015). Patients with HCT-CI scores ≥ 3 experienced a significant higher non-relapse mortality and inferior OS. Thus, in the current study, patients will be considered ASCT ineligible if they are 65 years or older, if their HCT-CI score is ≥ 3 regardless of age, or if they underwent prior consolidation with ASCT during first line therapy.

Patients with HCT-CI score ≥ 3, although not eligible for ASCT, may remain eligible to CAR-T cell therapy after evaluation by a CAR-T expert and if they meet all the pre-defined criteria described in the protocol. These criteria have been determined based on prior clinical trials and real-world experience which showed that CAR T-cell therapy is feasible and safed in such patients.

Patients will be considered eligible for CAR T-cell therapy based on the assessment of a CAR-T expert and if they meet all of the following criteria: ECOG performance status 0-2, absolute neutrophil count (ANC) ≥ 1000/µL, platelets ≥ 75,000/uL, absolute lymphocyte count ≥ 100/µL, creatinine clearance (as estimated by Cockcroft Gault) ≥ 40 mL/min, serum aminotransferase/aspartate aminotransferase (ALT/AST) ≤ 2.5xULN, total bilirubin ≤ 26 µmol/L, except in patients with Gilbert's syndrome, cardiac ejection fraction ≥ 45%, no evidence of pericardial effusion as determined by an echocardiogram (ECHO), and no clinically significant electrocardiogram (ECG) findings, no clinically significant pleural effusion, baseline oxygen saturation ≥ 92% on room air.

6 STUDY OBJECTIVES

6.1 Primary objective

The primary objective is to evaluate the Complete Metabolic Response (CMR) at 3 months from Axi-cel infusion (without additional anticancer therapy) based on investigator disease assessment according to PET-scan (using the Lugano Response Criteria).

6.2 Secondary objectives

The secondary objectives are:

- CMR at 3 months from Axi-cel infusion (without additional anticancer therapy) determined by central imaging review (using the Lugano Response Criteria)
- Event-free survival (EFS) at 3, 6 and 12 months from leukapheresis based on investigator disease assessment. EFS is defined as the time between leukapheresis and:
  - any event preventing Axi-cel infusion if Axi-cel is never infused, or
  - death, disease progression, or instauration of a new lymphoma therapy for lymphoma progression after Axi-cel infusion.
- EFS at 3, 6 and 12 months from leukapheresis based on central imaging review. EFS is defined as the time between leukapheresis and:
  - any event preventing Axi-cel infusion if Axi-cel is never infused, or
  - death, disease progression, or instauration of a new lymphoma therapy for lymphoma progression after Axi-cel infusion.
- Modified EFS (mEFS) at 6 and 12 months from leukapheresis. mEFS is defined as the time between leukapheresis and:
  - any event preventing Axi-cel infusion if Axi-cel is never infused, or
  - death, disease progression, or instauration of a new lymphoma therapy for lymphoma progression after Axi-cel infusion or failure to achieve a CMR at 6 and 12 months post-CAR infusion.
- Best objective response (complete and partial metabolic response)
- Duration of response (DOR)
- Progression-free survival (PFS) from Axi-cel infusion
- Overall survival (OS) from leukaphaeresis and Axi-cel infusion
- DOR, PFS, OS at 2 years and 3 years
- Safety of Axi-cel
- Health-related Quality of life (EORTC QLQ-C30, EQ-5D-5L and QLQ-NHL-HG29)

6.3 Exploratory objectives

Exploratory objectives:

To evaluate the mechanisms of action of Axi-cel (without additional anticancer therapy) with respect to efficacy and toxicity, in relation with:

- Baseline metabolic tumor volume and early metabolic response
- Characteristics of the tumor, the microenvironment, and the host
- Pre and post-treatment blood biomarkers including inflammation markers and PK/PD profiling
- Product characteristics and cellular kinetics *in vivo*
- Host immune response (including epitope spreading) and mechanisms of resistance

Exploratory endpoints:

- Imaging:
  - Correlation between Total Metabolic Tumor Volume (TMTV) pre-Axi-cel infusion and efficacy/toxicity
  - Correlation between early metabolic response (Day 14) and efficacy/toxicity
- Biology:

Biological exploratory endpoints will be described in relation with efficacy and toxicity and reported separately. They will be based on correlation with biological parameters such as:

- Histologic, phenotypic, genomic, transcriptomic, and molecular characteristics of malignant cells and tumor microenvironment pre and post treatment
- Cell product characteristics (*in vitro* proliferative potential, TCR repertoire, immunophenotype…) and cellular kinetics *in vivo* (such as peak concentration and persistence, etc)
- Immune response markers in tumor and blood such as cytokine levels, immune cells, and TCR repertoire.
- cfDNA concentration and mutational patterns evolution in plasma
- Immune-escape mechanisms including resistance of tumor cells to T-cell killing and tumor-induced immune suppression.

7 STUDY DESIGN

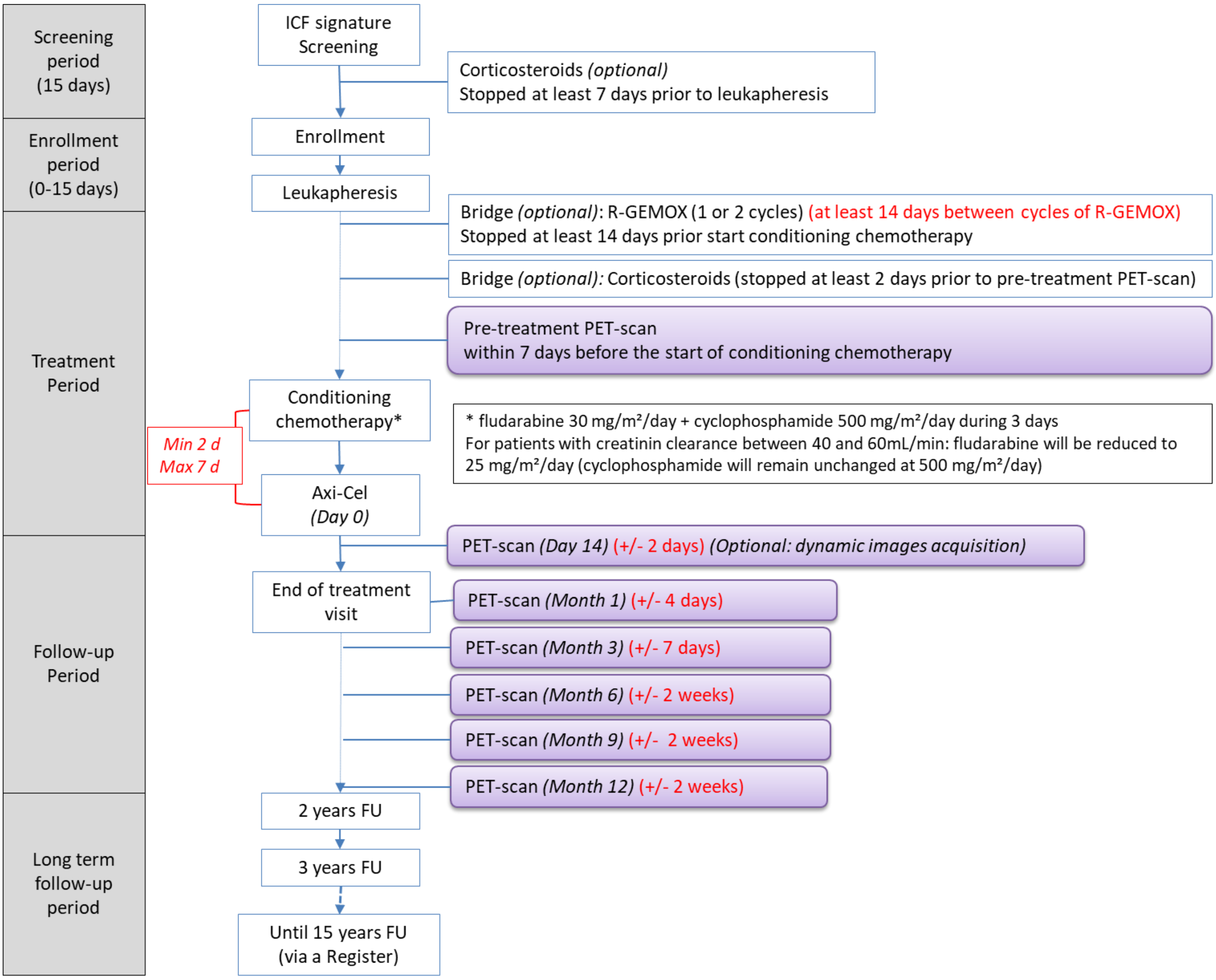

This study is a non-randomized, prospective, single arm, multicenter phase 2 trial.

Patients will be recruited over 14 months, treated with Axi-Cel and followed for up to 3 years after the last patient treated. The total duration of the study is therefore of 5 years.
The theoretical study dates (start / end) are:

- 1st patient enrolled (FPFV): 01/Dec/2020
- Last patient enrolled (LPFV): 28/Feb/2022
- Last patient followed for principal analysis: 01/May/2022.

A total of approximately 44 patients will be enrolled in the study for the main criterion analysis.
A total of 22 additionnal patients will be enrolled for subgroup analysis (analysis of the CMR based on the age and ECOG status depending on ECOG 2 subgroup size).
End of study is defined as end of 3 years of follow-up of the last patient treated in the study.

8 STUDY POPULATION

40 patients having received Axi-Cel infusion with aggressive B-NHL who are refractory or relapsed early after first-line therapy who are not eligible to autologous stem cell transplantation, but eligible to CAR T-cell therapy will be enrolled in this study.

20 additional patients having received Axi-Cel infusion with aggressive B-NHL who are refractory or relapsed early after first-line therapy who are not eligible to autologous stem cell transplantation, but eligible to CAR T-cell therapy will be enrolled in this study for an analysis based on age. Depending on the ECOG 2 subgroup size, an analysis could be also performed on ECOG status.

8.1 Inclusion criteria

Patients must meet the following criteria for study enrollment:

1. Signed written Informed Consent Form
2. Patient who understands and speaks one of the country official languages
3. Histologically proven relapsed or refractory aggressive B-cell non-Hodgkin lymphoma (B-NHL) of the following histology at relapse: diffuse large B-cell lymphoma (DLBCL), high-grade B-cell lymphoma (HGBL) and follicular lymphoma Grade 3B per WHO 2016 classification. Indolent B-NHL who transformed into aggressive B-NHL and were previously treated with R-CHOP are eligible. Primary mediastinal B-cell lymphoma are eligible.
4. Tumoral tissue (at diagnosis or relapse) available for central pathology review, exploratory endpoints and ancillary studies
5. Positron-emission tomography (PET)-positive disease
6. Patients must have received adequate first-line therapy including at a minimum:
    - An anti-CD20 monoclonal antibody (rituximab or obinutuzumab), and
    - CHOP or CHOP-like chemotherapy

<u>Note</u>: CHOP-like chemotherapy corresponds to ACVBP or EPOCH or COPADEM. Dose-reduced CHOP (i.e. miniCHOP) is excluded except for dose-reductions of vincristin due to peripheral neuropathy. Patients who have received additional drugs in combination with CHOP or CHOP-like regimen are eligible.

7. Relapsed or refractory disease after first-line chemoimmunotherapy (full dose of R-CHOP or R-CHOP-like regimen), documented by PET-scan:
    - <u>Relapsed disease</u> defined as complete remission to first-line therapy followed by biopsy proven disease relapse within 12 months from end of first-line therapy.
      Patients who received first line of R-CHOP or obinutuzumab-CHOP for an indolent B-NHL who relapse as transformed aggressive B-NHL within a year from the end of first-line therapy are eligible.
    - <u>Refractory disease</u> defined as:
        - ✓ Progressive disease (PD) during first-line therapy
        - ✓ Stable disease (SD) as best response after at least 4 cycles of first-line therapy (e.g. 4 cycles of R-CHOP)
        - ✓ Partial response (PR) as best response after at least 6 cycles, and biopsy-proven residual disease
8. At least 2 weeks must have elapsed since any prior systemic cancer therapy at the time the patient provides consent
9. Patients must be Autologous Stem Cell Transplantation (ASCT)-ineligible as defined by:
    - Patient deemed ineligible for high-dose chemotherapy and ASCT based on physician's assessment

- AND at least one of the following criteria:
  - ✓ Age ≥ 65 years or
  - ✓ Age ≥ 18 years and Hematopoietic Cell Transplantation-specific Comorbidity Index (HCT-CI – Appendix 09) score ≥3 or
  - ✓ Age ≥ 18 years and prior ASCT (as 1st line consolidation)

10. Patients must meet CAR-T-eligible as defined by:
    - Patient deemed eligible for CAR T-cells therapy by the CAR-T physician
    - AND all the following criteria:
      - ✓ ECOG performance status of 0, 1 or 2
      - ✓ Adequate vascular access for leukapheresis procedure (either peripheral or central venous line)
      - ✓ Absolute neutrophil count (ANC) ≥ 1 G/L
      - ✓ Platelets ≥ 75 G/L
      - ✓ Absolute lymphocyte count ≥ 0,1 G/L
      - ✓ Creatinine clearance (as estimated by Cockcroft Gault or MDRD) ≥ 40 mL/min
      - ✓ Serum alanine aminotransferase/aspartate aminotransferase (ALT/AST) ≤ 2.5xULN
      - ✓ Total bilirubin ≤ 26 µmol/L, except in patients with Gilbert's syndrome
      - ✓ Cardiac ejection fraction ≥ 45%,
      - ✓ Baseline oxygen saturation ≥ 92% on room air
11. Females of childbearing potential must have a negative serum or urine pregnancy test (females who have undergone surgical sterilization or who have been postmenopausal for at least 12 months are not considered to be of childbearing potential)

8.2 Exclusion criteria

Patients meeting any of the following criteria will not be enrolled in the study:

1. Patients who received more than one prior line of systemic therapy
2. Patients who are intolerant to first-line therapy or who received suboptimal first-line therapy, including dose-reduced R-CHOP ("R-miniCHOP"), and those who discontinued prematurely first-line therapy due to toxicity are not eligible (except for dose-reductions or discontinuation of vincristin due to peripheral neuropathy)
3. Prior CD19 targeted therapy
4. Patients with cardiac atrial or cardiac ventricular lymphoma involvement
5. Requirement for urgent therapy due to tumor mass effects, such as bowel obstruction or blood vessel compression
6. Patient with clinically significant pleural effusion
7. History of another primary malignancy that has not been in remission for at least 2 years (except for nonmelanoma skin cancer or carcinoma in situ (eg, cervix, bladder, breast)). A maintenance treatment is not allowed.
8. Patients with detectable Central Nervous System (CNS) lymphoma. Patients with a history of CNS lymphoma but no active CNS disease (after systematic MRI and lumbar puncture) at the time of enrollment will be eligible.

9. History or presence of non-malignant CNS disorder, such as seizure disorder requiring anti-convulsive therapy , cerebellar disease, or any autoimmune disease with CNS involvement disease
10. Active hepatitis B or hepatitis C infection at the time of screening

    Active Hepatitis B Virus (HBV) infection defined as:

    - HBs Ag positive

    - HBs Ag negative, anti-HBs antibody positive and/or anti-HBc antibody positive with detectable viral DNA
11. Positive serology of human immunodeficiency virus (HIV) and syphilis at the time of screening
12. Uncontrolled systemic fungal, bacterial, viral or other infection despite appropriate antibiotics or other treatment at the time of leukapheresis or Axi-cel administration
13. History of any one of the following cardiovascular conditions within the past 6 months: Class III or IV heart failure as defined by the New York Heart Association, cardiac angioplasty or stenting, myocardial infarction, unstable angina, or other clinically significant cardiac disease
14. History of autoimmune disease requiring systemic immunosuppression and/or systemic disease modifying agents within the last year
15. History of idiopathic pulmonary fibrosis, organizing pneumonia (eg, bronchiolitis obliterans), drug-induced pneumonitis, idiopathic pneumonitis, or evidence of active pneumonitis per chest computed tomography (CT) scan at screening. History of radiation pneumonitis in the radiation field (fibrosis) is allowed.
16. History of severe immediate hypersensitivity reaction to tocilizumab or any of the agents used in this study
17. History of severe, immediate hypersensitivity reaction attributed to aminoglycosides, cyclophosphamide and fludarabine
18. Treatment with a live, attenuated vaccine within 6 weeks prior to initiation of study treatment or anticipation of need for such a vaccine during the course of the study
19. Women of childbearing potential who are pregnant or breastfeeding because of the potentially dangerous effects of chemotherapy on the fetus or infant. Patients of either sex who are not willing to practice birth control from the time of consent during treatment and for at least 6 months after conditioning chemotherapy dosing or axicabtagene ciloleucel dosing, whichever is later
20. In the investigator’s judgment, the patient is unlikely to complete all protocol-required study visits or procedures, including follow-up visits, or comply with the study requirements for participation
21. Adult person unabled to provide informed consent because of intellectual impairment, any serious medical condition, laboratory abnormality or psychiatric illness.

9 STUDY FLOW CHART AND SCHEDULE OF ASSESSMENTS

9.1 Study flow chart

See on Appendix 01.

9.2 Schedule of assessments

See on Appendix 02 and 03.

9.3 Informed consent

To participate in this study and before any non-routine baseline or screening evaluation, each patient must be informed and have signed a written consent.

A written informed consent for biological studies and sample collections must be signed before the optional tumor sampling. A written informed consent for genetics analyzes must be signed before performing analyses on patient's constitituonnal DNA. Biological and genetic consents are optional. All these consents are signed after investigator gave all required information to the patient and the patient asked all his questions.

The patient and the investigator will date and sign the informed consent forms.

Two original copies of the signed consent will be completed. A copy will be provided to the patient; a copy will be maintained in the investigator's study file.

The investigator will attest on the eCRF that the patient has signed and dated the informed consent and indicate if the patient has signed the biological and genetic consents. The participation to the clinical trial will be tracked in the patient's medical record.

9.4 Baseline assessments

During the Multidisciplinary Team meeting in the hospital, the potential eligibility of the patient and administration of a bridge therapy will be discussed before ICF signature.

The patient's eligibility has to be evaluated during the screening period prior to the enrollment and leukapheresis. The assessments are to be performed from ICF signature to enrollment (**within 15 days**) **(except for diagnostic tissue and PET-scan).**

- Age, gender, weight, height, BSA (**Appendix 04 "Body Surface Area calculation"**)
- Clinical examination
- ECOG Performance Status (see **Appendix 05 "Performance Status Criteria"**)
- HCT-CI score (see **Appendix 09: "Hematopoietic Cell Transplantation-specific Comorbidity Index (HCT-CI)**")
- Vital signs (heart rate, blood pressure and body temperature) and oxygene saturation
- B symptoms
- Relevant medical history
  Any abnormal medical condition not due to lymphoma but already present before ICF signature, will be reported on medical history pages.
  Laboratory abnormal values before enrollment are not reported on haematology/biochemistry pages.
- History of the NHL.
- An archival tumor biopsy must be made available during the trial for confirmation and exploratory/ancillary studies of the disease (see section 11.2)
- Tumor biopsies at screening (before leukapheresis):
  - Mandatory if no archival tumoral tissue is available from diagnosis or relapse for review and exploratory/ancillary studies;
  - Highly recommended if archival tumoral tissue is available (from diagnosis or relapse);

  with at least:
    - 2 FFPE Tumor core needle (18 Gauge, or wider) biopsies

  - 1 Frozen Tumor core needle (18 Gauge, or wider) biopsy
  - 2 Tumor core needle (18 Gauge, or wider) biopsies in Cryostore®
  - Fine needle aspiration (25 Gauge) in Cryostore

- Complete blood cell count (CBC) will include hemoglobin, platelets, white blood cell (WBC) count, monocytes, absolute neutrophil count (ANC), absolute lymphocyte count (ALC),
- Biochemical tests: Sodium, potassium, glucose, serum creatinin, creatinine clearance according to MDRD/Cockcroft-Gault formula, AST, ALT, total bilirubin and alkaline phosphatases
- Lactate dehydrogenase (LDH), ferritine
- Fibrinogen, Prothrombin time (PT), activated Partial Thromboplastin Time (aPTT)
- C-reactive protein (CRP)
- HIV, Syphilis, HBV (Ag HBs, Ac anti-HBs, Ac anti-HBc and viral DNA) and HCV serologies
  Close monitoring of patients, with previous HBV or HVC infection, is expected to be performed, for clinical and laboratory signs of HBV or HCV reactivation, including monitoring of liver function tests and / or viral load according to local guidelines.

- Cerebral MRI
- Exploratory lumbar puncture:
  - Mandatory if prior history of CNS involvement by lymphoma or if clinically indicated (suspicion of CNS involement)
  - Otherwise optional
- Bone marrow biopsy and aspirate within 28 days of enrollment:
  - Mandatory if unexplained cytopenias or suspicion of bone marrow involvement
  - Otherwise optional
- ECG
- Echocardiography or isotopic method to determine resting ejection fraction
- Questionnaires of quality of life (EORTC QLQ-C30, QLQ-NHL-HG29, and EQ-5D-5L)
- Ann Arbor staging (Appendix 06)
- IPI score (Appendix 07)
- Pregnancy test on all women of child-bearing potential (within 15 days prior enrollment)
- Check on availability of PET-scan at relapse
- Schedule a neurological consultation prior to Axi-cel injection

9.5 Enrollment and Leukapheresis

If patient meet all eligibility requirements for enrollment, inclusion of the patient will be confirmed and leukapheresis will be scheduled via the plateform “Kite Clinical Logistics”. All details will be provided in the Investigational Product Manual.

If needed, patients are allowed to receive corticosteroids between screening and leukapheresis but corticosteroids (per os or IV route) must be discontinued at least 7 days prior to leukapheresis.

Leukapheresis will occur after enrollment but the patient’s eligibility has to be reevaluated until the day of leukapheresis regarding the following:

- No evidence or suspicion of ongoing infection

In case of uncontrolled systemic fungal, bacterial, viral or other infection despite appropriate antibiotics or other treatment detected between confirmation of eligibility and the start of leukapheresis, leukapheresis must be delayed until the event resolves (if more than 7 days, the participation of the patient should be rediscussed with the Coordinating Investigators).

- Corticosteroids (optional) must be discontinued at least 7 days prior to leukapheresis.

Please refer to paragraph 10.3.2 for more information on corticosteroids.

The following assessments will occur on the leukapheresis collection day:

- Vital signs (heart rate, blood pressure and body temperature, oxygene saturation)
- Concomitant medication at enrollment
- Weight, clinical examination
- Complete blood cell count (CBC) will include hemoglobin, platelets, white blood cell (WBC) count with monocytes, absolute neutrophil count (ANC), absolute lymphocyte count (ALC),
- Biochemical tests: Sodium, potassium, glucose, serum creatinin, creatinine clearance according to MDRD/Cockcroft-Gault formula, AST, ALT, total bilirubin and alkaline phosphatases
- Lactate dehydrogenase (LDH) (with Upper Normal Value)
- Ferritin
- Fibrinogen, PT, aPTT
- CRP
- Blood samples for biological banking and studies (+/- 2 days). Cf. paragraph 11.3
- Leukapheresis according to local practice
  Patients will undergo leukapheresis to obtain T cells for the manufacturing of axicabtagene ciloleucel. Leukapheresed cells will be shipped to the manufacturing facility as described in the IP Manual.
- Adverse/Serious Adverse Event recording

Optional: if needed, patients may receive bridging therapy (corticosteroids or R-GEMOX, refer to Section 10.3) after leukapheresis which should be discontinued at least 2 days prior to pre-treatment PET-CT for corticosteroids and at least 14 days prior to initiating conditioning chemotherapy for R-GEMOX.

9.6 Pre-treatment PET scan

A pre-treatment PET-CT will be performed within 7 days before the start of conditioning chemotherapy.
Patients receiving a bridge with corticosteroids must discontinue corticosteroids at least 2 days before this PET-CT.

9.7 Conditioning Chemotherapy Administration (Day - 5 through Day - 3 before infusion of axi-cel)

Approximately 2-4 weeks after leukapheresis procedure, patient will proceed with conditioning chemotherapy.

Administration of CAR-T cells to patients with ongoing infection or inflammation, even if such processes are asymptomatic, increases the risk of high grade and fatal toxicity.
All efforts should be made to rule out such conditions prior to administration of axi-cel.
Signs, symptoms, or abnormal laboratory results attributed to the malignancy (eg “tumor fever,” elevated C- reactive protein [CRP]) are diagnosis of exclusion that require a documented work-up to establish.

Conditioning chemotherapy and Axi-cel infusion should be initiated only once it is reasonably assured that cell infusion can safely proceed.

Requirements for Initiation of Conditioning Chemotherapy:
If any of the following criteria are met prior to initiation of conditioning chemotherapy, then the check-up list must be performed to determine the potential cause if there is no identified source of infection.

- Temperature > 38°Celsius within 72 hours of conditioning chemotherapy
- CRP > 100 mg/L anytime between enrollment to start of conditioning chemotherapy should be discussed with the Coordinating Investigators
- WBC count or WBC differential that is suggestive of infectious process, and is observed between enrollment and the initiation of conditioning chemotherapy (eg WBC > 20G/L or rapidly increasing WBC)

- Additionally: If any screening assessments or procedures are repeated between confirmation of eligibility and the start of conditioning chemotherapy and results are outside the eligibility criteria (Section 8), then the condition must resolve prior to proceeding with conditioning chemotherapy
- Complete history and physical/clinical exam including cardiac, vascular, respiratory, gastrointestinal, integumentary, and neurological systems must not reveal evidence of infection/inflammation
- The patient must not have received systemic anti-microbials for the treatment of a known or suspected infection within 48 hours before conditioning chemotherapy (prophylactic use of anti-microbials is allowed)
- Treatment course of any antimicrobials given for known or suspected infection should be completed as per infectious disease consult (if applicable) recommendation before stopping or switching to prophylactic antimicrobials
- If the patient is confirmed to have an infectious process for which antimicrobials are not available (eg, viral pneumonia), the infection must be clinically resolved as determined by the investigator and in consultation with infectious disease service (if applicable)
- The most recently collected blood, urine, or other body fluid cultures must show no growth for at least 48 hours, and any other infectious workup performed (eg, bacterial, viral serologies, PCR, stool studies, imaging studies) must be negative. If clinical suspicion is for an infection for which cultures are unlikely to be positive within 48 hours (eg, fungal infection), adequate time must be allowed for cultures to become positive.

Once the above criteria are met, then the patient can proceed with conditioning chemotherapy.

The following procedures will be completed during Day – 5 to Day – 3:

	When
Vital signs (pulse, blood pressure, body temperature, oxygene saturation)	Day – 5, Day – 4, Day – 3
Clinical examination	Day – 5
Weight	Day – 5
Complete blood cell count (CBC) will include hemoglobin, platelets, white blood cell (WBC) count, monocytes, absolute neutrophil count (ANC), absolute lymphocyte count (ALC), Lymphocytes B (units/µL) and atypical lymphoid cells (G/L).	Day – 5, Day – 4, Day – 3
Biochemical tests: Sodium, potassium, glucose, serum creatinin, creatinine clearance according to MDRD/Cockcroft-Gault formula, AST, ALT, total bilirubin and alkaline phosphatases	Day – 5, Day – 4, Day – 3
Ferritin	Day – 5, Day – 4, Day – 3
Fibrinogen, PT, aPTT	Day – 5, Day – 4, Day – 3
LDH	Day – 5, Day – 4, Day – 3
Gamma globulins	Day - 5 (before chemotherapy administration)
CRP	Day – 5, Day – 4, Day – 3
Questionnaires of quality of life (EORTC QLQ-C30, QLQ-NHL-HG29, and EQ-5D-5L)	Day – 5
Blood samples for biological studies and banking – see Table in 11.3	Day – 5
Fludarabine (30 mg/m²/day) and cyclophosphamide (500 mg/m²/day) administration	Day – 5, Day – 4, Day – 3

For patients with creatinin clearance between 40 and 60 mL/min: Fludarabine will be reduced to 25 mg/m²/day (cyclophosphamide will remain unchanged at 500 mg/m²/day)	
Adverse/Serious Adverse Event reporting	Day – 5, Day – 4, Day – 3
Concomitant medications documentation	Day – 5, Day – 4, Day – 3

The 3 days of chemotherapy will be followed by 2 rest days (Day - 2 and Day - 1) and infusion of axi-cel at the prescribed target dose on Day 0.

9.8 Assessments during treatment

9.8.1 Requirements for Axicabtagene Ciloleucel Infusion

Administration of Axi-cel to patients with ongoing infection or inflammation, even if such processes are asymptomatic, increases the risk of high-grade adverse events and/or fatal toxicity. All efforts should be made to rule out such conditions prior to cell infusion.
Signs, symptoms, or abnormal laboratory results attributed to the malignancy (eg “tumor fever,” elevated C- reactive protein [CRP]) are diagnoses of exclusion that require a documented work-up to establish.

Axi-cel infusion should be initiated only once it is reasonably assured that the treatment can safely proceed.

If any of the following criteria are met prior to the initiation of axicabtagene ciloleucel infusion, then the potential cause should be determined if there is no identified source of infection.

- Temperature > 38°Celsius within 72 hours of axicabtagene ciloleucel infusion.
- CRP > 100 mg/L anytime between enrollment to start of conditioning chemotherapy should be discussed with the Coordinating Investigators
- WBC count or WBC differential, that is suggestive of infectious process, and is observed between enrollment and the initiation of axicabtagene ciloleucel infusion (eg, WBC > 20 G/L) and rapidly increasing WBC)
- Additionally: If any screening assessments or procedures are repeated between confirmation of eligibility and the start of axicabtagene ciloleucel infusion and results are outside the eligibility criteria (Section 8), then the condition must resolve prior to proceeding with axicabtagene ciloleucel infusion (except for peripheral blood cell counts that have been impacted by conditioning chemotherapy)
- Complete history and physical/clinical exam including cardiac, vascular, respiratory, gastrointestinal, integumentary, and neurological systems must not reveal evidence of infection/inflammation
- The patient must not have received systemic antimicrobials for the treatment of a known or suspected infection within 48 hours before axicabtagene ciloleucel infusion (prophylactic use of antimicrobials is allowed)
- Treatment course of any antimicrobials given for known or suspected infection should be complete as per infectious disease consult (if applicable) recommendation before stopping or switching to prophylactic antimicrobials
- If a patient is confirmed to have an infectious process for which antimicrobials are not available (eg, viral pneumonia), the infection must be clinically resolved as determined by the investigator in consultation with infectious disease service (if applicable).
- Most recently collected blood, urine, or other body fluid cultures must show no growth for at least 48 hours, and any other infectious workup performed must be negative. If clinical suspicion is for an infection for which cultures are unlikely to be positive within 48 hours (eg, fungal infection), adequate time must be allowed for cultures to become positive.

After the above criteria are met, then the patient can proceed with infusion of axicabtagene ciloleucel.

If the axicabtagene ciloleucel infusion is delayed > 1 week from the planned infusion date, please contact the Coordinating Investigators.

9.8.2 Monitoring After Axicabtagene Ciloleucel Infusion

The patients will remain at the hospital for at least 10 days after infusion. Patients should not be discharged from the hospital until all axicabtagene ciloleucel related non-hematological toxicities resolve to ≤ Grade 1 or return to baseline. Patients may be discharged with non-critical and clinically stable or improving toxicities (eg, renal insufficiency) even if > Grade 1, if deemed appropriate by the investigator. Patients should remain in a hospital for ongoing axicabtagene ciloleucel related fever, hypotension, hypoxia, or ongoing neurologic events > Grade 1 or if deemed necessary by the investigator.
Patients should be instructed to remain within proximity of the hospital for at least 28 days following Axi-cel infusion.

The following assessements will be performed during hospitalization (for a minimum of 10 days):

	When
B-cells and T-cells (CD4 and CD8) will be quantified at Day 0.	D0
Weight	Daily
Vital signs (pulse, blood pressure, body temperature, oxygene saturation)	At least, every 8 hours
Clinical examination	Daily
ECOG PS	Daily
Complete blood cell count (CBC) will include hemoglobin, platelets, white blood cell (WBC) count monocytes, absolute neutrophil count (ANC), absolute lymphocyte count (ALC)	Daily, Day 14
Biochemical tests: blood ionogram, serum creatinin, creatinin clearance according to MDRD/Cockcroft-Gault formula, AST, ALT, total bilirubin and alkaline phosphatases	Daily
LDH, Ferritin	Daily, Day 14
CRP	Daily, Day 14
Fibrinogen, PT, aPTT	D0 and twice a week until D10 (additional assessment at investigator's discretion)
Adverse/Serious Adverse Event reporting	Daily
Evaluation of Cytokine Release Syndrome and neurotoxicity (please refer to Section 14.5 and Appendices 14 to 18 for grading and management guidance)	Daily
Registration of concomitant medication taken	Daily
Lumbar puncture	In case of Grade ≥ 2 ICANS (ASTCT)
Tumor core needle (18 Gauge or wider) biopsies: 2 fixed FFPE + 1 snap frozen + 1 frozen in Cryostor (for patients having consented to these optional biopsies)	D2 (+/-1 day)
Fine needle tumor aspiration (25 Gauge) in Cryostor (n=1)) (for patients having consented to these optional biopsies)	D2 (+/-1 day)
Blood samples for biological studies and banking – see Table in 11.3	D0 D1 D3 (+/-1 day) D5 (+/-1 day) D7 (+/-1 day) D10 (+/-2 days) D14 (+/-1 day)
PET-scan (dynamic images acquisition with Siemens Vision camera as described in Appendix 13 can be performed by sites with this camera)	D14 (+/- 2 days)

9.9 End of treatment and permanent study discontinuation assessments

Leukapheresis without infusion of axicabtagene ciloleucel is considered as a permanent study discontinuation.

After completing axicabtagene ciloleucel infusion and completing the observation period, all patients will be followed in the follow-up period.

Patients will be evaluated within 1 month (+/- 4 days) of Axi-cel infusion or within 28 days following permanent treatment discontinuation:

- Physical examination and body weight
- Vital signs (pulse, blood pressure, body temperature)
- ECOG PS
- Complete blood cell count (CBC) will include hemoglobin, platelets, white blood cell (WBC) count with monocytes, absolute neutrophil count (ANC), absolute lymphocyte count (ALC). In addition, B-cells and T-cells (CD4 and CD8) will be quantified at Month 1.
- Biochemical tests: blood ionogram, serum creatinin, creatinin clearance according to MDRD/Cockcroft-Gault formula, AST, ALT, total bilirubin and alkaline phosphatases
- Adverse events and toxicities
- PET scan (Month 1) and disease status
- LDH, ferritine
- Gamma globulins
- Bone marrow aspirate and biopsy in patients with evidence of bone marrow involvement at baseline or with persistent unexplained cytopenias
- Questionnaires of quality of life (EORTC QLQ-C30, QLQ-NHL-HG29, and EQ-5D-5L) (M1)
- Serious adverse events
- Concomitant medications documentation
- Blood samples for biological studies and banking (M1) – see table 11.3 -

9.10 Follow-up assessments (between 3 months – 1 year)

9.10.1 Patients who have completed treatment or discontinued treatment due to reasons other than progressive disease or relapse (meaning patients who did not experienced progression or relapse)

After Axi-cel infusion, patients will be followed every 3 months during the first year.

An update of the following parameters will be done at Month 3 (+/- 7 days), Month 6 (+/- 2 weeks), Month 9 (+/- 2 weeks) and Month 12 (+/- 2 weeks):

- Physical examination and body weight
- Complete blood cell count (CBC) will include hemoglobin, platelets, white blood cell (WBC) count with monocytes, absolute neutrophil count (ANC), absolute lymphocyte count (ALC). In addition, B-cells and T-cells (CD4 and CD8) will be quantified at Month 3, 6, 9 and 12.
- ECOG Performance Status (see Appendix 05 "Performance status criteria")
- Ongoing treatment (including G-CSF, EPO, and transfusions)
- PET scan (Month 3, M6, M9, M12) and disease status
- LDH, ferritine
- Gamma globulins (Month 3, M6, M9, M12)
- Questionnaires of quality of life (EORTC QLQ-C30, QLQ-NHL-HG29, and EQ-5D-5L) (M3, M6, M12)
- Pregnancy test on all women of child-bearing potential (Month 6)
- Related serious adverse events
- Blood samples for biological studies and banking (M3, M6, M9, M12) – see table 11.3

9.10.2 Patients who experienced progressive disease or relapse

For relapse/progression determination and assessments (PET-scan and biopsy), please refer to Section "9.12 Progression/relapse".

Only survival status and other malignancies will be recorded at every evaluation period.

Update of following information will be recorded approximately every 6 months during the first year, then every year during the next 2 years.

- Overall survival
- Other malignancies

The patients will be followed as long as possible up to the end of study or until death.
Patients enrolled in the study who withdraws before study treatment start will not be followed.

9.11 Long term follow-up period assessments (≥ 1year)

The window for long term follow-up period assessments is +/- 2 weeks.
After the first year, the patients will be followed every year for up to 2 years with documentation of:

- Relapse (with date of relapse) versus remission status
- Overall survival,
- Questionnaires of quality of life (EORTC QLQ-C30, QLQ-NHL-HG29, and EQ-5D-5L) (M24 and M36)

After the end of protocol, patients will be followed until 15 years via DESCAR-T, french register of patients with malignant hemopathies and eligible for car-t cell therapy for:

- Late toxicities (AESI and SAE considered related to Axi-cel)
- Relapse and any new anti-lymphoma therapy,
- Death.

Visits will be done at M48, M60,10 and 15 years.

9.12 Progression/relapse

Relapse/progression will be determined as per Lugano classification 2014 criteria **(see Appendix 10** "Response criteria for lymphoma")

Progressive disease should be based on PET scan and/or clinical information.

A pathological confirmation should be performed which will include at least 4 tumor core needle biopsies (18 Gauge, or wider) with at least: 2 FFPE and 1 frozen biopsy for biological studies and the 4th for banking with Cryostore freezing medium.
Whenever possible, 1 Fine needle tumor aspiration of the tumor (25G) should also be performed and stored according to table 11.3 and laboratory manual.
Additionaly, blood samples for biological banking and biologicial studies will be performed at time of relapse/progression.

10 TREATMENTS

The Investigational Medicinal Product (IMP) is named axicabtagene ciloleucel.
The conditioning chemotherapy regimen used for this study will be fludarabine and cyclophosphamide.
Bridging therapy is optional and refers to treatment used to control a patient's disease prior to conditioning chemotherapy.

10.1 Investigational Medicinal Product description, storage and handling

10.1.1 Description

Axicabtagene ciloleucel is a CD19-directed genetically modified autologous T cell immunotherapy. To prepare axicabtagene ciloleucel, a patient's own T cells are harvested and genetically modified ex vivo by retroviral transduction to express a chimeric antigen receptor (CAR) comprising a murine anti-CD19 single chain variable fragment (scFv) linked to CD28 and CD3-zeta co-stimulatory domains. The anti-CD19 CAR T cells are expanded and infused back into the patient, where they can recognize and eliminate CD19-expressing target cells.
Axicabtagene ciloleucel is prepared from the patient's peripheral blood mononuclear cells, which are obtained via a standard leukapheresis procedure. The mononuclear cells are enriched for T cells and activated with murine anti-CD3 antibody in the presence of recombinant human IL-2, followed by transduction with the replication incompetent murine retroviral vector containing the anti-CD19 CAR transgene. The transduced T cells are expanded in cell culture, washed, formulated into a suspension, and cryopreserved. The finished product must pass all the release testing including sterility before Qualified Person (QP) release for shipping as a frozen suspension in a patient-specific infusion bag. The product is thawed prior to infusion using established methodology.

Refer to the most current IB and IP Manual.

10.1.2 Packaging and labeling

Axicabtagene ciloleucel is supplied cryopreserved in cryostorage bag. The product in the bag is slightly cloudy, with cream to yellow color. The cryostorage bag containing axicabtagene ciloleucel is delivered frozen in vapour phase of liquid nitrogen (LN_2) (≤150°C).
Each sterile single infusion bag contains a dispersion for infusion of chimeric antigen receptor (CAR)-positive T cells in approximately 68 mL. The target dose is 2.0×10^6 anti-CD19 CAR T cells per kg body weight, with a maximum of 2.0×10^8 CAR-positive viable T cells.

10.1.3 Storage conditions

The bag must be stored in vapor phase of liquid nitrogen and the product remains frozen until the patient is ready for treatment to assure viable live autologous cells are administered to the patient. Several inactive ingredients are added to the product to assure viability and stability of the live cells through the freezing, thawing, and infusion process.

10.1.4 Handling

Axicabtagene ciloleucel is a patient-specific (autologous) product and the intended patient will be identified by a unique patient ID number. Upon receipt, verification that the product and patient-specific labels match the patient's information (e.g., patient ID number) is essential. Do not infuse the product if the information on the patient-specific label does not match the intended patient.
The product should be thawed by qualified personel according to the IP Manual and administrated to the patient within 3 hours. The thaw start/stop time and axicabtagene ciloleucel administration start/stop time, will be noted in the patient medical record.

10.2 Treatment schedule and design

10.2.1 Bridging Therapy (administered after leukapheresis) – optional

Bridging therapy refers to treatment used to control a patient's disease or disease related inflammation prior to conditioning chemotherapy.

- Corticosteroids:

Information will be recorded in eCRF (dose per day).
Administration should be terminated at least 2 days before pre-treatment PET-scan.
For more information, please refer to paragraph 10.3.2.

- R-GEMOX

Administration of 1 or 2 cycles of R-GEMOX should be terminated 14 days before conditioning chemotherapy with at least 14 days between cycles of R-GEMOX. The dosage will be according local standard of care (usually Rituximab at 375 mg/m², gemcitabine at 1000 mg/m² and oxaliplatin at 100 mg/m²).
Information (date of cycle and dosage) will be recorded in eCRF.

10.2.2 Conditioning chemotherapy

Patients will receive a non-myeloablative conditioning regimen consisting of cyclophosphamide and fludarabine in order to induce lymphocyte depletion and create an optimal environment for expansion of axicabtagene ciloleucel in vivo. Patients will initiate conditioning chemotherapy with cyclophosphamide and fludarabine beginning on Day -5 through Day -3.

- Fludarabine

Fludarabine will be administered at 30 mg/m²/day during 3 consecutive days.

For patients with creatinin clearance between 40 and 60 mL/min: Fludarabine will be reduced to 25 mg/m²/day.

- Cyclophosphamide

Cyclophosphamide will be administered at 500 mg/m²/day during 3 consecutive days.

10.2.3 Axicabtagene Ciloleucel

All patients will receive axicabtagene ciloleucel infusion in the hospital followed by daily monitoring in the hospital for at least 10 days to monitor signs and symptoms of cytokine release syndrom and neurologic toxicities.
Patients should not be discharged from the hospital until all axicabtagene ciloleucel related non-hematological toxicities return to baseline. Patients may be discharged with non-critical and clinically stable or improving toxicities (eg, renal insufficiency) even if > Grade 1, if deemed appropriate by the investigator.
Patients should remain in a hospital for ongoing axicabtagene ciloleucel-related fever, hypotension, hypoxia, or ongoing central neurologic toxicities > Grade 1, or if deemed necessary by the investigator.

If infusion cannot be performed within 7 days after the conditioning chemotherapy regimen, this will be discussed with Coordinating Investigators on case by case basis.

10.3 Concomitant treatment

Any medication (except homeopathy, phytotherapy) and procedure from leukapheresis and up to 30 days after infusion of AxiCel will be considered as concomitant treatments.
All concomitant treatments and blood transfusions will be reported in eCRF.

10.3.1 Prohibited therapies

The following concomitant treatments are not permitted after Axi-cel infusion:

- Systemic anticancer agents.
- Other investigational anticancer therapies.

If a patient's clinical status requires administration of a prohibited concomitant medication or treatment, then the patient will be withdrawn from the study treatment.
The change in clinical status mandating the use of the medication in question must be reported as the reason for study drug discontinuation.

10.3.2 Restricted/allowed therapies

During screening period, corticosteroids (per os or IV route) may be used up to 1 mg/kg of prednisone or equivalent and must be discontinued at least 7 days prior to leukapheresis.

However, cutaneous administration of corticosteroids is allowed (within 7 days prior to leukapheresis).

Patients will be instructed not to take any additional medications (including over-the-counter products) during the course of the study without prior consultation with the investigator.
All therapies necessary for the patient management are permitted besides other antineoplasic agents for lymphoma. The use of antibiotics, growth factors and other supportive therapies is at the discretion of the treating physician and should be recorded in the case report form if pertinent and/or linked to adverse event.
Platelet and red blood cell transfusions are permitted, as necessary.

Prophylactic antiviral treatment with anti HBV therapy (such as entecavir or tenofovir) for Ab HBc + is mandatory.

Anti-infective prophylaxis: it is expected that these prophylactic antibiotic treatments will be established in consultation with the local infectious disease specialist, and that resorting to other anti-infective agents (antivirals, antiparasitics, antifungals) will be done in consultation with the relevant infectious disease specialists in the field.

10.4 Drug Dispensation

10.4.1 Responsibilities

The investigational site is an authorized treatment center. Specialized site staff at each site is trained to infuse Axi-cel and to properly monitor and care for patients.

The investigator, the Hospital Pharmacist, or other personnel allowed to store and dispense Investigational Medicinal Product (Axi-cel) are responsible for ensuring that the IP used in the clinical trial are securely maintained as specified by the Sponsor and in accordance with the applicable regulatory requirements. All Investigational Medical Products are stored in accordance with labeling and must be dispensed in accordance with the investigator's prescription. The investigator and the pharmacist are responsible of maintening an accurate record of Investigational Product issued and returned. **The product traceability at site must be available as it could be asked by the sponsor at any moment during the study.** Any quality issue noticed with the receipt or use of an Investigational Product (deficient IMP in condition, appearance, pertaining documentation, labeling, expiry date, etc.) should be promptly notified to the Sponsor, who will initiate a complaint procedure. Under no circumstances will the investigator supply Investigational Medicinal Product to a third party, allows the Investigational Medicinal Product to be used other than as directed by this Clinical Trial Protocol, or dispose of Investigational Product in any other manner.

10.4.2 Retrieval or destruction

All unused IPs will be destroyed by the pharmacist.

All IPs will be destroyed by the pharmacist. All destroyed treatments will have to be documented by the pharmacist (destruction certificate).

In case of a potential defect in the quality of Investigational Product, the Sponsor may initiate a recall procedure. In this case, the investigator or pharmacist will be responsible for promptly addressing any request made by the Sponsor, in order to recall Investigational Product and eliminate potential hazards.

10.4.3 Accountability and compliance

The investigator or pharmacist will inventory and acknowledge receipt of all shipments of the investigational medicinal product. The investigator or pharmacist will also keep accurate records of the volume of Axi-cel infused, the thaw start/stop time, and axicabtagene ciloleucel administration start/stop timefor each patient.

All unused investigational products and all medication containers will be destroyed by the study sites according to local procedure. The Sponsor will verify that a final report of drug accountability is maintained and archived in the investigator study file. Administration of the study treatment will be supervised by the investigator or sub-investigator.

10.5 Prophylactic measures

Investigators may prescribe any treatment deemed necessary to provide adequate supportive care, including growth factor support (eg, G-CSF, EPO), antibioprophylaxis and routine antiemetic prophylaxis except those medications listed in the excluded medication paragraph 10.3.1.
This will be recorded in eCRF.
All volume of blood required by patient during the study will be collected in a specific form in eCRF.

11 STUDY PROCEDURES

11.1 Screening and enrollment procedure

As soon as a patient has signed the ICF, he / she should be registered by the investigators or delegated site staff directly on the data capture system, through the internet network, using the address below.

Data Capture System (e-CRF): https://lysarc.ennov.com/EnnovClinical/login?etude=ALYCANTE

The patient creation should be done before starting the protocol assessments. The study site will receive back the registration number for the registered patient.

The investigator should fax **(+33 (0)4 06 27 40 13**) at the same time the following documents whatever the registration way used:

- LYSARC enrollment form
- pseudonymized copy of the initial/relapse pathology report with:
    - name and address of the pathologist having diagnosed the lymphoma easily identified
    - report reference number easily identified
    - remain only the first letter of the first name and the first letter of the last name, month and year of birth, if authorized by local regulation
    - delete hospital identification number and day of birth or more if required by local regulation)

LYSARC coordination center (Tel: +33 (0)4 72 66 93 33) will be the point of contact for any request.

11.2 Pathological diagnosis

The study requires a histological review of all cases enrolled in the trial. The aims of the centralized histopathological review will be to confirm the diagnosis of Relapsed/Refractory aggressive B-NHL lymphoma, according to the criteria of the updated WHO classification 2018 (S. Swerdlow et al.) for each patient. Histological criteria of inclusion and exclusion have been detailed in the current protocol.

At the time of enrollment, the centralization of the histopathological material review process will be organized by the LYSA-Pathology institute, Hopital Henri Mondor, Créteil, France (LYSA-P) and the review process will be performed with the help of Camille Laurent laboratory.

Therefore for each enrolled patient, tumor tissue blocks - or only when not possible - unstained slides and frozen tumor biopsy will have to be sent for analysis and confirmation of diagnosis to LYSA-P.

In eCRF we will also collect the histological report numbers for tumoral biopsies and Bone marrow biopsies (BOM).

11.2.1 Patient enrollment

At patient enrollment, the investigator will be requested to fax to LYSARC, with the enrollment form, a copy of the initial and relapse histopathological pseudonymized report on which the name and address of the pathologist having diagnosed the aggressive B-NHL lymphoma and the report reference number will be easily identified, as well as the pseudonymized bone marrow report when available (cf. § 11.1).

LYSARC will then fax/mail these documents to LYSA-P and to the referant pathologist.

11.2.2 Sample request

LYSARC/LYSAP will organize the centralization of the material including tumor samples at diagnosis and at relapse/progression.

All pseudonymized copy of pathological reports will be collected by LYSARC.

11.2.3 Sample centralisation at LYSA-P

All these materials will be sent in prepaid envelope (ambient) and centralized by LYSA-P at the following address:

LYSA-P, LYSA – ALYCANTE study
Hôpital Henri Mondor
51, avenue de Lattre de Tassigny
94010 Créteil
France

11.2.4 Sample review

At sample reception, a pathological review will be organized with the designated panel of pathologists for this study. All the cases will be reviewed by at least 2 expert hematopathologists and a consensus diagnosis will be set and registered in LYSA-P data base. This consensus diagnosis will then be sent to the clinical investigator and to the initial pathologist.
All the cases will be reviewed and characterized by the LYSA reviewers.
Initial +/- relapse and other tumor samples available will also be used to study the expression of markers known to influence the prognosis of aggressive B-NHL lymphoma.

DNA/RNA will also be extracted from the tumor samples to further characterize lymphoma prognostic biomarkers. RNA will be extracted from the frozen tumor sample for the biological study purposes. If frozen samples are not available RNA will be extracted from the tumor FFPE samples.

For the need of ancillary studies, samples will be kept temporarily to avoid a second request. Meanwhile, the block will be at the entire disposition of the initial anatomopathology laboratory under request if they need it.

11.3 Biological banking studies

The ALYCANTE study gives the opportunity to collect tumor and blood samples before, during and after treatment of aggressive B-NHL Lymphoma with Axi-Cel therapy. Biological samples collection will be performed in France and Belgium. Analysis of Axi-cel pharmacokinetics, pharmacodynamics, as well as some other specialized biomarkers analysis, will be performed by Kite (USA).

The following samples will be derivated from already existing material:

- Tumor blocks and slides from the diagnosis/first relapse or progression tumor block
- CAR-T cells products from infusion lot.

The following additional samples are recommended before treatment (mandatory if no tumoral tissue is available from diagnosis/relapse) with at least:

- On site FFPE inclusion of 2 core needle biopsies at screening
- On site inclusion of frozen sample of 1 needle biopsy at screening
- On site freezing of 2 core needle biopsies in Cryostore at screening

On site preparation of a cell pellet of pooled tumor fine needle aspiration in Cryostore at screening
The following samples are mandatory before and during treatment:

- On site preparation and freezing of PBMCs for pharmacokinetic analysis, through 11 timepoints and in case of relapse/progression
- On site preparation and freezing of serum, for cytokines analysis through 11 timepoints
- Fresh blood shipment for immunophenotypic and CyToF analyzes and PBMC banking, through 11 timepoints and in case of relapse/progression
- Fresh blood shipment for cell-free DNA through 9 timepoints and in case of relapse/progression

- Fresh blood shipment for TCR repertoire assessment throught 11 timepoints and in case of relapse/progression
- Bone marrow biopsy and aspiration are mandatory within 28 days of enrollment in patients with unexplained cytopenias or suspicion of bone marrow involvment
- Bone marrow biopsy and aspiration will have to be repeated at Month 1 in case of baseline involvement or if persistent unexplained cytopenias.

The following additional samples are recommended during treatment and in case of relapse/progression with at least:
- On site FFPE inclusion of 2 core needle biopsies at D2 (+/-1 day) and at relapse/progression
- On site inclusion of frozen sample of 1 core needle biopsy at D2 (+/-1 day) and at relapse/progression
- On site freezing of 1 core needle biopsy in Cryostore at D2 (+/-1 day) and at relapse/progression
- On site preparation of a cell pellet of pooled tumor fine needle aspiration in Cryostore at D2 (+/-1 day) and at relapse/progression

The following samples are collected in case of prior history of CNS involvement by lymphoma or if clinically indicated (suspicion of CNS involement) / otherwise optional and in case of ICANS ≥ grade 2 (ASTCT):
- On site preparation of CSF (cerebrospinal fluid)

Proposed analysis will support to study objectives of the trial (see section 6.3).

Requirements for preparation, shipment and material supply are described in the Biological Studies Manual and summarized hereafter.
LYSARC will organize the centralization of the biological materials including tumor samples, blood, CSF and bone marrow at diagnosis and/or during/after treatment and/or at relapse/progression.

11.3.1 Informed consents

The informed consent form (ICF) for participation to the study must be signed before taking any sample from the patient (except initial tumor biopsy). The study informed consent form will include all mandatory biological sampling. For all other biological sampling, a specific consent for biological samples collection and future use must be signed. Preservation of samples taken, analyzes and genetic researches on them are optional. They are governed by specific terms in Belgium:

- The patient must express his consent for biological samples collection. This agreement is mandatory for sample preparation and preservation. Terms have to be explained, proposed and the form signed before taking any sample.

- If genetic studies are planned, the patient must express his consent for studies on his genetic characteristics. This agreement is mandatory for analysis on the patient's constitutional DNA that would be extracted from banked samples thanks to the previous consent form for biological collection. Terms have to be explained, proposed and signed in addition of the consent for collection if previously agreed.

11.3.2 Biological banking and studies

Exploratory objectives and assessments on biological samples will be fully described in a separate document.

Material		Sampling	Schedule: At screening	At leukapherisis	D-5	D0 (before CAR-T)	D1	D2 +/-1	D3 +/-1	D5 +/-1	D7	D10 +/-2	D14	M1	M3	M6	M9	M12	Prog/Relapse	if ICANS ≥ grade 2 (ASTCT)	On site preparation	Destination
Archival tumor tissue (diag or relapse)	FFPE	Archival block	×																		FFPE inclusion	LYSA-P/Toulouse, FR
		≥ 10 unstained slides	×																		FFPE inclusion	LYSA-P/Toulouse, FR
		Immunostained slides	×																		FFPE inclusion	LYSA-P/Toulouse, FR
	FROZEN	Frozen biopsy	×																		Freezing	LYSA-P/Toulouse, FR
TUMOR biopsy (a)	FFPE	1st and 2nd core needle biopsy	×					×											×		FFPE inclusion	LYSA-P/Toulouse, FR
	FROZEN	3rd core needle biopsy	×					×											×		Flash freezing	LYSA-P/Toulouse, FR
	CRYOSTOR® FROZEN	4th core needle biopsy	×					×											×		Cryostor	LYSA-P/Rennes, FR
		5th core needle biopsy	×																		Cryostor	LYSA-P/Rennes, FR
		Fine needle aspiration	×					×											×		Cryostor	LYSA-P/Rennes , FR
BLOOD	frozen cells (c)	12mL, CPT heparinized			×	×				×	×	×	×	×	×	×	×	×	×		Immediate shipment	Kite, USA
	serum (c)	5mL SST tube (gold top)		× (b)	×	×	×		×	×	×	×	×	×	×						Gathering at the end of study	On site --> Kite, USA
	cells	Fresh blood 16ml, heparin tubes		× (b)	×	×					×	×	×	×	×	×	×	×	×		Immediate shipment	Rennes, FR
	DNA	Fresh blood 9mL Strek® tube		× (b)	×	×							×	×	×	×	×	×	×		Immediate shipment	Rennes, FR
PRODUCT	final product formulation	CAR-T cells products not infused				×																On site --> Rennes, FR
CSF	frozen	Cells, cytokines	× (d)																	×	Centrifuge, freezing	On site --> Rennes, FR
Maximum blood volume (mL)				30	42	42	5		5	17	33	33	42	42	42	37	37	37	37			

(a) Tumor biopsy: mandatory if no archival tumor tissue available, highly recommended otherwise

(b) +/- 2 days is allowed if timepoint does not occur on a working day

(c) Analysed by Kite. PBMC for cellular kinetics of CAR-T cells and serum for cytokines and RCR (replication-competent retrovirus).

(d) CSF: Mandatory if prior history of CNS involvement by lymphoma or if clinically indicated (suspicion of CNS involement) / otherwise optional

A missing timepoint does not cancel following samples.

Cryopreserved manufacturing retains will be taken at Leukapheresis and for final product formulation to enable translational research described. Samples will support PK analysis and exploratory analysis of product phenotypes, T-cell fitness and product function. These specific samples and any other derivatives from these samples may be stored by Kite up to 15 years to address exploratory research scientific questions related to the treatment or disease under study. Each patient will have the right to have the sample material destroyed at any time by contacting the investigator who, in turn, can contact the central laboratory. The investigator should provide the sponsor with the study and subject number so that the sample can be located and destroyed.

For patients who withdraw consent, any samples that were not requested to be returned or destroyed will remain with the sponsor, and any data that may be generated will be entered in the study database.

11.3.3 Process

11.3.3.1 On site frozen samples

Instructions for preparation, shipment and material supply for

- frozen sample of core needle biopsy and FNA,
- tumor cells preparation and freezing in Cryostor system (possibility of freezing in 90%FCS/10%DMSO if cryostor system not available),
- infused CAR-T sampling

are described in the Biological Studies Manual.

Frozen CSF (supernatant and cells) dedicated to CAR-T quantification will be gathered at least at the end of the study by LYSARC with a special carrier respecting dry ice storage. All samples will be then shipped and analyzed at:

CRB Biothèque Hématologie

CHU Pontchaillou de Rennes
Batiment Médico-Technique
Hématologie Biologique 1er étage
2, rue Henri Le Guilloux
35043 Rennes - FRANCE

Kite Pharma, Inc.
2400 Broadway
Santa Monica, CA 90404

11.3.3.2 Centralized fresh samples

11.3.3.2.1 Centralized fresh samples – to Rennes, FR

At each indicated timepoint (11 and relapse/prog) take a 16mL blood sample on 4 Heparin-Lithium tubes and 9mL blood sample on Streck tube. Fresh blood shipment must be planned and ordered by site depending on patient's visits. Pickup orders must be sent to the carrier the working day before the day of visit and sampling, taking into account vacation days:

Laboratoire SITI
CHU Pontchaillou de Rennes
Batiment Médico-Technique
1er étage
2, rue Henri Le Guilloux
35043 Rennes - FRANCE

Material is provided by LYSARC in "biological kits" including material, labels, tracking forms and pickup orders. An instruction manual is provided during SIV and on demand to detail procedures for sample drawing, preparation and shipment. First kits will be complete and supplied during Site Initiation Visits, then new kits will be automatically resupplied after each enrollment.

11.3.3.2.2 Centralized fresh samples – to Kite, USA

At each indicated timepoints (11 and relapse/prog) take a 12 ml blood sample on 3 CPT Heparin-Lithium tubes. Fresh blood shipment must be planned and ordered by site depending on patient's visits, and send to:

Kite Pharma, Inc.
2400 Broadway
Santa Monica, CA 90404

Material is provided by KITE in "biological kits" including material, labels, tracking forms and pickup orders. An instruction manual is provided during SIV and on demand to detail procedures for sample drawing, preparation and shipment. First kits will be complete and supplied during Site Initiation Visits, then new kits will be automatically resupplied after each enrollment.

11.3.3.3 On site frozen samples for pharmacology. Shipment to USA

- Instructions for preparation, shipment and material supply for cytokines samples (serum) are described in the Biological Studies Manual

- Specific shipment will be organized for analysis at

Kite Pharma, Inc.
2400 Broadway
Santa Monica, CA 90404

11.4 PET scan Review

A central review of the PET scan is organized for this study and is mandatory.

For each patient when applicable, the following data and images will be reviewed by a panel of PET experts:

- Baseline PET scans (at 1st relapse/progression)
- Pre treatment PET scans (before lymphodepletion)
- PET Day 14 (If the center is equiped with a Siemens vision PET/CT, a dynamic acquisition must be performed. This specific acquisition corresponds to the fully-automated acquisition protocol of Siemens FlowMotion Multiparametric PET Suite Patlak and is described on the Appendix 13)
- PET Month 1, 3, 6, 9, 12

The review processing is described on the Appendix 12.

11.5 Quality of life questionnaires

- Quality of life (EORTC QLQ-C30, EQ-5D-5L, EORTC QLQ-NHL-HG29)

The EORTC QLQ-C30, EQ-5D-5L and EORTC QLQ-NHL-HG29 questionnaires will be measured as described in section 20.2 schedule of Evaluations (study flow-chart). Details of the questionnaires are precise in appendix 08.
The ***EORTC QLQ-C30*** is a validated self-rating cancer-specific questionnaire including 30 items. It is composed of multi-item and single-item scales. The QLQ-C30 includes:

- One scale for Global Health Status / Quality of Life (QOL)
- Five Functional Scales:
  - Physical Functioning;
  - Role Functioning;
  - Emotional Functioning;
  - Cognitive Functioning;
  - Social Functioning.
- Nine Symptom Scales:
  - Fatigue;
  - Nausea/Vomiting;
  - Pain;
  - Dyspnea;
  - Insomnia;
  - Appetite Loss;
  - Constipation;
  - Diarrhea;
  - Financial Difficulties.

All scales and single items meet the standards for reliability. The reliability and validity of the questionnaire is highly consistent across different language-cultural groups. One score is generated per scale following the recommendation

of the EORTC and standardized on a 0 to 100 scale in order that a high score reflect a high Global Health Status/QoL score, a high functional level and a high symptomatic level.

The ***EQ-5D-5L*** has two components, the EQ-5D-5L descriptive system and the EQ-5D-5L visual analog scale (VAS). The EQ-5D system comprises five dimensions: mobility, self-care, usual activities, pain/discomfort and anxiety/depression. Each dimension has five levels: no problems, slight problems, moderate problems, severe problems and extreme problems. The patient is asked to indicate his/her health state by ticking the box next to the most appropriate statement in each of the five dimensions. This decision results in a 1-digit number that expresses the level selected for that dimension. The digits for the five dimensions can be combined into a 5-digit number that describes the patient's health state. A single summary index is obtained by applying a formula that attaches weights to each of the levels in each dimension (Chevalier et al, 2013).
The EQ VAS records the patient's self-rated health on a vertical visual analogue scale, marked 0 (worst imaginable health state) to 100 (best imaginable health state). The VAS can be used as a quantitative measure of health outcome that reflect the patient's own judgement.

The **QLQ-NHL-HG29** is a validated self-rating module specific to high grade non hodgkin's Lymphoma including 29 items. The items are conceptualized in several multi-item scales. The QLQ-NHL-HG29 includes:

- Symptom burden
- Physical condition/fatigue
- Worries/fears on health and functioning
- Emotional impact
- Neuropathy

For each scale, one score is generated following the recommendation of the EORTC and standardized on a 0 to 100 scale in order that a high score reflect a high symptomatic level.

12 STUDY COMMITTEES

12.1 Centralized Review Committee (CRC)

The Centralized Review Committee (CRC) will perform a blinded, independent assessment of the metabolic response by comparison with pre-treatment PET/CT (before lymphodepletion) and the corresponding date of the progression or relapse, during the first year, for each patient according to the Deauville scale and Lugano Classification (2014), measures of ΔSUVmax and LYRIC criteria will be also recorded as exploratory. The CRC will be composed of an independent review of all PET scans performed by two independent nuclear physicians (with an additional in case of discrepancy).

13 CRITERIA FOR PERMANENT TREATMENT DISCONTINUATION OF THE STUDY

13.1 Permanent treatment discontinuation

Circumstances that lead to permanent treatment discontinuation of a patient from the trial must be reported by the investigator on the appropriate CRF page.

Criteria for patient permanent treatment discontinuation include (but are not limited to):

- Death,
- toxicity of study treatment, that would be, in the investigator's opinion, detrimental to the patient's well-being
- lymphoma progression,
- concomitant disease,
- major protocol violation, including initiation of alternate anti-neoplasic therapy.

Patients who are in permanent treatment discontinuation should however remain in the trial for the purpose of follow-up and data analysis, with the exception of patients who withdrew their consent. Any patient who discontinues before completing the study will be encouraged to return to the study centre within 4 weeks for an evaluation.

13.2 Withdrawal of Consent

Patients are free to withdraw from the study at any time without prejudice to their treatment. When a patient decides to withdraw from the study, he/she should always be contacted in order to obtain information about the reason for withdrawal and to record any adverse events. When patient agrees, he/she should return for a study visit at the time of, or soon after withdrawal, and the relevant assessments should be performed.

It should be asked to the patient if he/she agrees that his/her data continue to be collected until the end of the study. If the patient explicitly states his/her wish not to contribute further data to the study, the relevant LYSARC contact should be informed and the withdrawal of consent should be documented by the investigator in the patient's case report form. However, data up to the time of consent withdrawal will be included in the data reported for the study.

13.3 Patients Lost to Follow up

Every effort will be made to contact patients who fail to return for scheduled visits. A patient is considered lost to follow-up if no information has been obtained when the last patient has completed the clinical phase of the study. During this time site investigator must document at least 3 attempts to contact the patient either by phone or letter.

13.4 Discontinuation of the study

The sponsor reserves the right to stop the trial at any time. The investigators will be informed of this decision in writing. Study discontinuation will also be declared to CA and EC according to local regulation.

The same applies to any investigator wanting to discontinue his/her participation to the trial. The investigator must immediately inform the sponsor in writing of this decision.

14 SAFETY PARAMETERS

14.1 Definitions

14.1.1 Adverse Events

An **adverse event** (AE) is any untoward medical occurrence in a patient or clinical investigation patient administered a pharmaceutical product and which does not necessarily have to have a causal relationship with this treatment.
An AE can therefore be any unfavorable and unintended sign (including an abnormal laboratory finding, for example), symptom, or disease temporally associated with the use of a medicinal product, whether or not considered related to the medicinal product. This includes any occurrence that is new in onset or aggravated in severity or frequency from the baseline condition.

14.1.2 Serious Adverse Events

A **serious adverse event** (SAE) is any untoward medical occurrence that at any dose:

- Results in death
- Is life-threatening (the term "life-threatening" in the definition of "serious" refers to an event in which the patient was at risk of death at the time of the event ; it does not refer to an event which hypothetically might have caused death if it were more severe)
- Requires inpatient hospitalization (hospitalization is defined as an inpatient admission, regardless of length of stay) or prolongation of existing hospitalization
- Results in persistent or significant disability/incapacity (the term “persistent or significant disability or incapacity” means that there is a substantial disruption of person’s ability to carry out normal life functions.)
- Is a congenital anomaly/birth defect
- Is a medically significant event.

Medical and scientific judgment should be exercised in deciding whether expedited reporting is appropriated in other situations, such as important medical events that may not be immediately life-threatening or result in death or hospitalization but may jeopardize the patient or may require intervention to prevent one of the outcomes listed in the definition above.
The term “severe” is a measure of intensity, thus a severe AE is not necessarily serious. For example, “nausea of several hours duration” may be severe but may not be clinically serious.

14.1.3 Intensity

The intensity of the AE or SAE will be graded by the investigator according to the Common Terminology Criteria for Adverse Events (CTCAE) grading system v5.0 in the toxicity categories that have recommended grading (see investigator’s file or online at http://ctep.cancer.gov/protocolDevelopment/electronic_applications/ctc.htm).
AEs not listed on this grading system will be graded according to the five-point system below:

- Mild (grade 1): Discomfort noticed but no disruption of normal daily activity
- Moderate (grade 2): Discomfort sufficient to reduce or affect normal daily activity
- Severe (grade 3): Incapacitating with inability to work or perform normal daily activity
- Life-threatening (grade 4): Substantial risk of dying at time of event
- Death (grade 5)

14.2 Adverse Events reporting rules

AEs of grade ≥ 2 (CTCAE – version 5.0),all grades (ASBMT Consensus Grading and Lee, 2014) for Cytokine Release Syndrome and all grades (CTCAE and ASBMT Consensus Grading) for Neurologic Toxicity (ICANS) regardless of relationship to investigational product occurring **after leukapheresis procedure to 30 days after Axi-cel infusion will be recorded in the AE pages of the eCRF**. When associated to a SAE and regardless the time of occurrence and the grade, the AE must be reported as “Adverse Event” in the appropriate eCRF pages.

Whenever possible, symptoms should be grouped as a single syndrome or diagnosis. The investigator should specify the date of onset, intensity, action taken regarding Axi-cel, corrective therapy given, outcome of all AEs and his opinion as to whether the AE can be related to the Axi-cel.

All events that meet one or more criteria of seriousness (see Section 14.1.2) will be reported as SAE (see Section 14.3).

General AE reporting rules:

- Non-serious AE will be reported through eCRF
- Any episode of any grade of toxicity, related to a SAE must be reported as "Adverse Event" in the appropriate eCRF pages regardless the time of occurrence
- Signs, symptoms and physical findings indicative of lymphoma or progression of lymphoma are not to be reported as "Adverse Event"
- AE with onset date after new lymphoma treatment administration are not to be reported
- "Alopecia" toxicity (any grade) will never be reported as "Adverse event"
- AEs will be considered ended (recovered without sequelae) when recovered to a grade 0 or baseline
- In case of screening failure, at least AEs corresponding to SAEs will be reported in the AEs pages of eCRF
- When a medical history resolves or decreases at a grade lower than baseline, the new grade will be the new reference grade for following AEs
- For laboratory abnormalities, the laboratory test to be taken as reference will be the one performed nearest to the **Axi-cel infusion**
- Error in Treatment Administration**:** An incorrect administration of Axi-cel is not itself an adverse event, but it may result in an adverse event and should be reported as an AE.

Abnormal laboratory values reporting rules:

If a laboratory abnormality is one component of a diagnosis or syndrome (e.g., alkaline phosphatase and bilirubin 5 × ULN associated with cholecystitis), only the diagnosis (e.g., cholecystitis) or syndrome should be recorded on the AE page/screen of the eCRF. If the abnormality was not a part of a diagnosis or syndrome, then the laboratory abnormality should be recorded as the AE.

An abnormal laboratory value (≥ grade 2 only) which is not a component of a diagnosis or syndrome is considered as an AE if the abnormality:

- results in discontinuation from the study; or
- requires treatment, modification/ interruption of IP dose, or any other therapeutic intervention; or
- is judged to be of significant clinical importance.

The investigator has to notify in the patient medical file all the abnormal laboratory values considered as clinically significant (write next to each abnormal laboratory value assessed as clinically significant "CS" or precise it in the medical report).

Regardless of severity grade, only laboratory abnormalities that fulfill a seriousness criterion need to be documented as a serious adverse event.

Second primary malignancies (SPM) reporting rules:

Second primary malignancies will be monitored as events of interest and must be reported as serious adverse events. This includes any SPM, regardless of causal relationship to study treatment (Axi-cel), occurring at any time for the duration of the study, from the leukapheresis procedure up to the end of follow-up period.

Events of SPM are to be reported using the SAE report form and must be considered an "Important Medical Event" even if no other serious criteria apply; these events must also be documented in the appropriate page(s) of the eCRF and subject's source documents. Documentation on the diagnosis of the SPM must be provided at the time of reporting as a serious adverse event (e.g., any confirmatory histology or cytology results, X-rays, CT scans, etc.).

14.3 Serious Adverse Events reporting rules

All events that meet one or more criteria of seriousness (see Section 14.1.2) occurring **after leukapheresis procedure to end of treatment evaluation (30 days after Axi-cel infusion),** whatever the grade, will be reported as SAE regardless:

- the relationship to the study treatment
- the administration of new lymphoma therapy
- disease progression*

**Events with a criterion of severity have to be reported as SAE regardless of disease progression if they are not related to it.*

A SAE that occurs after this time, including during the follow-up period, **if considered related to Axi-cel**, will be reported.

Whenever possible, symptoms should be grouped as a single syndrome or diagnosis. The investigator should specify the date of onset, intensity, action taken regarding trial medication, corrective therapy given, outcome of all SAEs and his/her opinion as to whether the SAE can be related to the study drugs.

General SAE reporting rules:

- Any episode of any grade of toxicities, which meets one of the seriousness criteria must be reported as "Serious Adverse Event" in the appropriate SAE form
- The following events are not to be reported as SAE if require hospitalization less than 8 days:
  - Hematological toxicities (anemia, thrombocytopenia, leucopenia, neutropenia), febrile neutropenia, nausea, vomiting
  - Life-threatening and fatal events should be reported as SAE regardless the duration of hospitalization
- Signs, symptoms and physical findings indicative of lymphoma or progression of lymphoma are not to be reported as "Serious Adverse Event". However in case the patient had progressed and a SAE occurred, if the cause of SAE is not lymphoma, it is a SAE to report.
- SAE with onset date after new lymphoma treatment administration are not to be reported, except if considered related to Axi-cel or to the research
- "Alopecia" toxicity (any grade) will never be reported as "Serious Adverse Event"
- Hospitalizations **not to be considered** as SAEs are:
  - Planned hospital admissions or surgical procedures for an illness or disease which existed before the patient was enrolled in the study or before Axi-cel infusion are not to be considered SAEs unless the condition deteriorated in an unexpected manner during the study (e.g., surgery was performed earlier than planned).
  - A procedure for protocol therapy administration or protocol/disease-related investigations. Hospitalization or prolonged hospitalization for a complication will be reported as an SAE
  - Routine treatment or monitoring of the studied indication (e.g., administration of blood or platelet transfusion) not associated with any deterioration in condition. Hospitalization or prolonged hospitalization for a complication remains a reportable SAE
  - Hospitalization or prolongation of hospitalization for technical, practical, or social reasons, in absence of an AE
  - Emergency outpatient treatment or observation that does not result in admission, unless fulfilling other seriousness criteria above (section 14.1.2)

14.3.1 Obligations of the investigator

In a case of SAE the investigator must immediately (within 24 hours):

- **Complete SAE form with all relevant information regarding SAE**
- **SEND the SAE pages to**

LYSARC Pharmacovigilance department
FAX: +33 (0)3 59 11 01 86
Email: pharmacovigilance@lysarc.org

All SAE forms must be dated and signed by the responsible Investigator or one of his/her authorized staff Members.

- May attach the photocopy of all examinations carried out and the dates on which these examinations were performed. Care should be taken to ensure that the patient's identity is protected and the patient's identifiers in the Clinical study are properly mentioned on any copy of source document. For laboratory results, include the laboratory normal ranges.
- Follow up of any SAE that is fatal or life-threatening should be provided within one calendar week.

For SAEs, the following must be assessed: relationship to study drug (Axi-cel), action taken, and outcome to date. The assessment of whether there is a reasonable possibility of a causal relationship is usually made by the investigator; it can be one of two possibilities:

- Unrelated (no reasonable possibility)
- Related (reasonable possibility)

Items to be considered when assessing the relationship of a SAE to the study drug are:

- Temporal relationship of the onset of the event to the initiation of the study drug
- The course of the event, considering especially the effect of discontinuation of study drug or reintroduction of study drug ifapplicable
- Whether the event is known to be associated with the study drug or with other similar treatments
- The presence of risk factors in the study patient known to increase the occurrence of the event presence of non-study drug-related factors which are known to be associated with the occurrence of the event.
- If there is no clear evidence suggesting a causal link but the influence of other factors is unlikely
- Whether an alternative etiology has been identified
- Mechanism of action of the study drug
- Biological plausibility

14.3.2 Obligations of the Sponsor

During the course of the study, the Sponsor will report in an expedited manner all SAEs that are both unexpected and at least reasonably related to study drugs, to the EMA, Health Authorities, Ethic Committees in each country in accordance with international and local regulations. The causality assessment given by the investigator should not be downgraded by the sponsor. If the sponsor disagrees with the investigator's causality assessment, the opinion of both the investigator and the sponsor should be provided with the report.

The expectedness of a serious adverse reaction will be determined by the Sponsor according to the reference safety information (Investigator's Brochure) of the study drug.

LYSARC Pharmacovigilance department will report all safety information from the trial in the Development Safety Update Reports and will notify the reports to the Health Authorities and Ethics Committees in accordance with international and local regulations.

14.4 Follow up of AEs and SAEs

All AEs and SAEs should be monitored until it is resolved or is clearly determined to be due to a patient's stable or chronic condition or underlying condition, regardless of relationship with IMP and whether the patient received a new anti-lymphoma therapy or not. If the patient left the trial or after study end, only AEs and SAEs possibly related to study will be followed up. Any additional information known after the event has been initially reported should be sent to LYSARC as soon as information becomes available.

Patients who are in permanent study treatment discontinuation due to any AE will be followed at least until the outcome is determined even if it implies that the follow-up continues after the patient has left the trial.

Ongoing adverse events thought to be related to IMP will be followed until the event is resolved to baseline grade, or is assessed by the investigator as stable or new anti-lymphoma treatment is initiated for progression of the underlying disease.

14.5 Adverse Events of Special Interest

The following AEs are considered as of special interest and require attention from investigator if occurring and should be reported after leukapheresis procedure **to the end of follow-up** of the patient. In addition, they have to be reported as soon as possible, but preferably no later than 2 weeks, in the eCRF, irrespective of the seriousness criteria, and from the Grade 1. They have to be signaled to LYSARC Pharmacovigilance using the AESI form. If the definition of SAE is met, they should be reported using the SAE form only.

- **Cytokine Release Syndrome (CRS)**

Nearly all patients will experience some degree of CRS.
Please ensure that a minimum of 4 doses of tocilizumab, an anti interleukin-6 (IL-6) receptor blocking antibody, are available for each patient prior to infusion of Axi-cel.
Patients should be monitored daily for signs and symptoms of CRS for at least 10 days following infusion. After that, the patient should be monitored at the investigator's discretion.

The clinical presentation of macrophage activation syndrome/hemophagocytic lymphohistiocytosis is similar to that of CRS, with similar initial therapeutic treatment. In the event of non-response to treatment for CRS, the need of re-adjust treatment and initied alternative therapy should be quickly assessed and discussed with the Coordinating Investigators.

For CRS grading, please refer to **Appendix 14 - ASTCT CRS Grading** and **Appendix 15 - CRS Grading Scale (Excluding Neurologic Events) per Lee, 2014**. Both scales of grading should be used.
For management of CRS, please refer to the **Appendix 16** and Investigator's Brochure. CRS will be managed based on the Lee criteria (Appendix 15 & 16) but patients will also have to be graded with the ASTCT grading system (Appendix 14) for record.

- **Neurotoxicities**

This product is a CAR T-cell and some CAR T-cells have been associated with unexplained deaths from cerebral edema.
Moreover, combination with fludarabine can also induce neurotoxicity.
Patients should be monitored at least daily for 10 days at the investigational site following infusion for signs and symptoms of neurologic toxicity. After the first 10 days following the infusion, the patient should be monitored at the investigator's discretion. Monitoring of vital signs and organ functions should be considered depending on the severity of the reaction.
The neurotoxicities will be graded by the investigator according to CTCAE and ASTCT Grading for Neurologic Events (**Appendix 17 - ASTCT Grading for Neurologic Events**).
For management of neurotoxicities, please refer to the **Appendix 18** and Investigator's Brochure. Neurotoxicity will be managed based on CTCAE grading system but patients will also have to be graded with the ASTCT grading system (Appendix 17) for record.

- **Prolonged/delayed cytopenia** (beyond month 1)

All blood transfusion will be recorded in eCRF

- **Hypogammaglobulinemia**

B-cell aplasia leading to hypogammaglobulinaemia can occur in patients receiving treatment with Axi-cel. Immunoglobulin levels should be monitored after treatment with Axi-cel and managed using infection precautions, antibiotic prophylaxis, and immunoglobulin replacement.

- **Documented infections**

It includes any infection associated with a germ (bacteria, virus, fungus, parasite) or developed in an organ system (even in the absence of an identified germ).
This includes bacteremia and excludes positive tests without symptoms and therefore without an associated clinical situation.

- **Emergent secondary malignancy**

They should always be reported using the SAE form.

14.6 Pregnancy

14.6.1 Females of Childbearing Potential

This protocol defines a female of childbearing potential as a sexually mature woman who: 1) has not undergone a hysterectomy or bilateral oophorectomy or 2) has not been naturally postmenopausal (amenorrhea following cancer therapy does not rule out childbearing potential) for at least 12 consecutive months (i.e., has had menses at any time in the preceding 12 consecutive months).

The highly effective birth control methods are the following:

- combined (estrogen and progestogen containing) hormonal

- contraception associated with inhibition of ovulation:

- oral
  - intravaginal
  - transdermal

- progestogen-only hormonal contraception associated with inhibition of ovulation:
  - oral
  - injectable
  - implantable

- intrauterine device (IUD)

- intrauterine hormone-releasing system (IUS)

- bilateral tubal occlusion

- vasectomized partner

- sexual abstinence

The duration of the contraception should be during treatment and for at least 6 months after conditioning chemotherapy dosing or axicabtagene ciloleucel dosing, whichever is later.
Pregnancies and suspected pregnancies (including a positive pregnancy test regardless of age or disease state) of a female patient occurring within 30 days after the patient's Axi-cel infusion are considered events to be reported immediately to LYSARC Pharmacovigilance on the appropriate Pregnancy Form.

LYSARC fax number +33 (3) 59 11 01 86
Email: Pharmacovigilance@lysarc.org

The female should be referred to an obstetrician/gynecologist preferably one experienced in reproductive toxicity for further evaluation and counseling.

The Investigator will follow the female patient until completion of the pregnancy and must notify LYSARC immediately about the outcome of the pregnancy (either normal or abnormal outcome).

If the outcome of the pregnancy is abnormal (i.e., spontaneous or therapeutic abortion) the Investigator should report the abnormal outcome as an AE. If the abnormal outcome meets any of the serious criteria, it must be reported as an SAE within 24 hours of the Investigator's knowledge of the event using the SAE Report Form or approved equivalent form.

All neonatal deaths that occur within 30 days of birth should be reported, regardless of causality, as SAEs. In addition, any infant death after 30 days that the Investigator(s) suspects to be related to the in-utero exposure to the study drug should also be reported to LYSARC within 24 hours of the Investigator's knowledge of the event using SAE form.

14.6.2 Male patients

For female partner in childbearing potential of a male patient taking study drug, same highly effective birth control methods are applicable.

It is recommended for males included in this trial to not father a child for 6 months after conditioning chemotherapy dosing or axicabtagene ciloleucel dosing, whichever is longer.

If a female partner of a male patient taking study drug becomes pregnant, the male patient taking study drug should notify the Investigator, and the pregnant female partner should be advised to call her healthcare provider immediately.

If a pregnancy related event is reported in a female partner of a male patient, the investigator should determine whether the female partner is willing to release her medical information to LYSARC Pharmacovigilance and allow the pregnancy related event to be followed-up to completion.

15 GENERAL STATISTICAL CONSIDERATIONS

15.1 Primary endpoint

The primary endpoint is the complete metabolic response (CMR) at 3 months from Axi-cel infusion (without additional anticancer therapy) based on investigator disease assessment (INV). The CMR is the number of responder patients divided by the number of patients included in the analysis set. Assessment will be based on the Lugano classification. The primary analysis will be performed on the modified Full Analysis set (cf §15.5). A sensitivity analysis will be performed on the Full Analysis Set (cf §15.5).

15.2 Secondary efficacy endpoints

Secondary efficacy endpoints will include:

- Complete Metabolic response (CMR) determined by central review (IRC)

Complete Metabolic response at 3 months from Axi-cel infusion (without additional anticancer therapy) based on central review (IRC). Assessment of response will be based on the Lugano classification. The categorization of the patients according to response assessment will be performed as follows:

- CMR = Responder
- PMR = Non-responder
- SD/PD = Non-responder
- Missing evaluation = Non-responder

- Event-free survival (EFS) at 3, 6 and 12 months from leukapheresis based on investigator disease assessment (INV) and by central review (IRC)

EFS is defined as the time between leukapheresis and:
- any event preventing Axi-cel infusion if Axi-cel is never infused, or
- death, disease progression, or instauration of a new lymphoma therapy for lymphoma progression after Axi-cel infusion.

Patients without documented event at the time of analysis will be censored at the time of last visit with adequate assessment.

- Modified EFS (mEFS) at 6 and 12 months leukapheresis based on investigator disease assessment (INV) and by central review (IRC)

mEFS is defined as the time between leukapheresis and:
- any event preventing Axi-cel infusion if Axi-cel is never infused, or
- death, disease progression, or instauration of a new lymphoma therapy for lymphoma progression after Axi-cel infusion or failure to achieve a CMR at 6 months post-CAR infusion.

Patients without documented event at the time of analysis will be censored at the time of last visit with adequate assessment.

- Best objective response (CMR and PMR)

Best objective response is defined as the percentage of CMR+PMR determined investigator disease assessment (INV) among all patients between D14 and M12 from Axi-cel infusion. Patient without response assessment (due to whatever reason) will be considered as non-responder. The categorization of the patients according to response assessment will be performed as follows:

Evaluation at D14, M1, M3, M6 and M12	Best overall response (CMR+PMR)
CMR	Responder
PMR	Responder
SD/PD	Non-responder
Missing evaluation	Non-responder

- Duration of response (DOR)

Duration of response is defined as the time from attainment of PMR or CMR to the date of first documented disease progression/relapse (based on investigator disease assessment (INV)) or death from any cause. Patients alive and free of progression will be censored at the time of last visit with adequate assessment.

- Progression-free survival (PFS)

PFS is defined as the time from Axi-cel infusion to the first observation of documented disease progression/relapse (based on investigator disease assessment (INV)) or death due to any cause. If a patient has not progressed or died, PFS will be censored at the time of last visit with adequate assessment.

- Overall survival (OS)

Overall survival will be measured from date of Axi-cel infusion to the date of death from any cause. Alive patients will be censored at their last follow-up date.

- Quality of life (EORTC QLQ-C30, EQ-5D-5L ,EORTC QLQ-NHL-HG29)

The compliance to QoL questionnaire will be described at baseline and each follow-up assessment. QoL scores obtained from each questionnaire will be described at baseline using mean and standard deviation. Mean change from baseline, including all patients with at least baseline and one post-baseline assessment, will be described at each follow-up assessment. A linear mixed model will be also explored. The minimal clinically important difference (MCID) to interpret the results will be fixed to 10 points for each scale of the EORTC (Osoba et al., JNCI 1998), 0.08 points for the EQ-5D utility score and 7 points for the EQ-5D VAS (Pickard AS et al, 2007). The percentage of patients in deterioration, stable or improved compared to the baseline will be reported at each follow up time point using the MCID value to qualify the change. The time until definitive deterioration (TUDD) will also be assessed and defined as the time from inclusion in the study to the first clinically significant difference compared to the baseline, without further clinically significant improvement or death. Median TUDD will be reported with its 95% confidence interval.

15.3 Secondary safety endpoints

- **Adverse Events**

All adverse events will be described.

Adverse events observed will be classified using MedDRA System Organ Class and Preferred Term. The severity of the toxicities will be graded according to the NCI CTCAE v5.0 whenever possible; NCI CTCAE v3.0 will be used to grade tumor flare reaction.

The frequency of adverse events will be tabulated by MedDRA System Organ Class and Preferred Term. In the by-patient analysis, a patient having the same event at the same grade more than once will be counted only once. Adverse events will be summarized by NCI CTCAE grade. Adverse events leading to discontinuation from treatment, events classified as NCI CTCAE grade 3 or 4 study-drug-related events, deaths, and SAEs will be tabulated and listed separately. By-patient listings of all AEs, SAEs, and their attributes will be provided.

AEs leading to death, and pregnancies will also be displayed in a separate table and a by-patient listing.

- **Deaths**

All deaths will be listed and also summarized by cause of death.

15.4 Exploratory endpoints

- Correlation between Total Metabolic Tumor Volume (TMTV) pre-Axi-cel infusion and efficacy/toxicity
- Correlation between early metabolic response (Day 14) and efficacy/toxicity
- Histologic, phenotypic, genomic, transcriptomic, and molecular characteristics of malignant cells and tumor microenvironment
- Cell product characteristics (*in vitro* proliferative potential, TCR repertoire, immunophenotype…) and cellular kinetics *in vivo* (such as peak concentration and persistence)
- Immune response markers in tumor and blood such as cytokine levels, immune cells, and TCR repertoire.
- Plasma cfDNA concentration
- Immune-escape mechanisms including resistance of tumor cells to T-cell killing and tumor-induced immune suppression.

15.5 Analysis sets

15.5.1 Enrolled Set (ES)

The Enrolled Set (ES) will include all patients having signed their informed consent.

15.5.2 Full Analysis Set (FAS)

The Full Analysis Set (FAS) will include all patients having signed their informed consent and with a confirmed enrollment. This set will be used in a sensitivity analysis to ensure the robustness of the results obtained with the modified Full Analysis Set.

15.5.3 Modified Full Analysis Set (mFAS)

The modified Full Analysis Set (mFAS) includes all patients in the FAS with infusion of Axi-cel. This set will be used for demographic and baseline characteristics and efficacy analysis.

15.5.4 Safety Set (SS)

The Safety Set (SS) will include all patients having signed their informed consent and who received infusion of Axi-cel. This set will be used for safety analysis.
Note: For the purpose of this study, the modified Full Analysis set and the Safety set are the same.

15.5.5 QoL Set

The QoL population may vary depending on the subscale analyzed and the number of patients with evaluable scores for that subscale. Evaluable scores will be defined separately for each measure and will be summarized in the Statistical Analysis Plan. Patients will be included in analysis of change from baseline if they have an assessment at baseline and at least one post-baseline.

15.5.6 Age subgroup

An age-based subgroup will be set up to split patients under the age of 70 (strictly) from others. The aim of this subgroup is to assess if the main criterion is reached in the patients aged ≥ 70 years (more fragile patients) as in the other set of patients (age <70) with a sufficient power.

15.5.7 ECOG subgroup

An ECOG-based subgroup will be set up to split patients with ECOG 0-1 from others (only if percent of patients with ECOG≥2 is greater than 10%). The main criterion would be analysed.

15.6 Statistical methods

15.6.1 Patients replacement

No patient with Axi-cel infusion will be replaced even if no assessment is performed at 3 months from Axi-cel infusion.

15.6.2 General Approach

Continuous data will be summarized in tables displaying sample size, mean, standard deviation, median, range; quartiles will also be presented when considered relevant.

Categorical data will be described in counts and percentages (of non missing data)

Response rates (according to Lugano 2014): will be expressed with 90% confidence limits (to be consistent with one side 5% level of significance) according to Pearson-Clopper method. The number and percent of patients falling into each category of response will be provided.

Time to event will be performed using Kaplan-Meier method and comparison between categories will be made with the Log-Rank test. A Cox proportional hazard model will be used to estimate the hazard ratio (HR) and associated 95% CI. Survival probabilities, median survival and quartiles will be estimated with their 95% CI. Survival curves will be provided.

The EORTC QLQ-C30 questionnaire will be analyzed according to the functional scores and the recommendations in the EORTC scoring manual. All these recommendations will be described in the Statistical Analysis Plan.

The **EQ-5D-5L questionnaire** will be analyzed according to the recommendations in the scoring manual. All these recommendations will be described in the Statistical Analysis Plan.

The **EORTC QLQ-NHL-HG29** questionnaire will be analyzed according to the recommendations in the EORTC scoring manual. All these recommendations will be described in the Statistical Analysis Plan.

15.6.3 Efficacy Analysis

The efficacy analyses will be based on the Modified Full Analysis set.

15.6.4 Safety Analysis

The safety analysis will be based on the Safety set.

15.7 Sample size

The primary endpoint in the study is the Complete Metabolic Response (CMR) at 3 months from Axi-cel infusion based on investigator disease assessment (INV).
Sample size calculation was performed with EAST 6.5 using an exact single-stage phase II design[3].
No interim analysis is planned.
It is hypothesized that the 3 months-CMR is 12% with SOC (based on historical controls) and 34% with Axi-cel infusion.
The assumptions are as follows:

- improvement of the CMR at 3 months from 12% to 34%
- one-sided alpha: 5%
- Power: 96%

Based on these assumptions, 40 evaluable patients are needed. Patients are considered as evaluable if patients are included in the modified Full Analysis Set. Assuming a 10% of dropt out (i.e patients in the Full Analysis Set but not undergoing Axi-cel infusion), an overall sample size of approximatively 44 patients needs to be enrolled. Patients having signed their informed consent and with a confirmed enrollment (FAS) will be included in this study.

It is necessary to ensure these assumptions are similar between patients who are over and under the age of 70.
It is hypothesized that distribution will be equivalent in both age subgroups.
In order to have a sufficient power in each subgroup, a minimum of 25 patients is required (power of 80%) and 30 patients are expected (power of 85%). Thirty (30) evaluable patients in each subgroup will allow to have a sufficient power and to prevent possible unbalanced age distribution.
This will require the addition of 20 evaluable patients. Assuming a 10% of drop-out, 22 additionnal patients need to be enrolled.
Enrollment will be stopped once 60 patients have been infused with Axi-Cel (mFAS).

15.8 Analyses

15.8.1 Interim analysis

No interim analysis is planned for CMR rate (primary endpoint).

15.8.2 Final analysis

<table>
<tr><th>Calendar of analysis</th><th>mFAS - 40 patients
Cut-off</th><th>mFAS – 62 patients
Cut-off</th></tr>
<tr><td>Main criterion analysis</td><td>3 months after 40th infused patients</td><td rowspan="2">6 months after 62 infused patients*</td></tr>
<tr><td>Secondary endpoints analysis</td><td>6 months after 62 infused patients*</td></tr>
<tr><td>Update of the secondary endpoints analysis</td><td colspan="2">12 months after 62 infused patients</td></tr>
<tr><td rowspan="2">Update of the survival endpoints analysis</td><td colspan="2">24 months after 62 infused patients*</td></tr>
<tr><td colspan="2">36 months after 62 infused patients**</td></tr>
</table>

* to avoid multiple analysis
** combined with end of study analysis

Main criterion analysis

The final CMR analysis will be performed when the concerned patients (40 then 62 for subgroup analyses) have been performed the investigator disease assessment at 3 months from Axi-cel infusion.

Secondary endpoints analysis (CMR rate by IRC, EFS, ORR, DOR, PFS, OS)

It will occur when all evaluable patients have been performed the investigator disease assessment at 6 months from Axi-cel infusion, have died, have withdrawn consent, or are lost to follow-up, whichever occurs first.
For exploratory purpose, the CMR according to the investigator assessment will be analyzed on the mFAS by age subgroup on the 60 evaluable patients. Secondary endpoints will also be analyzed by age subgroup.

Update of the secondary endpoints analysis (CMR rate by IRC, EFS, ORR, DOR, PFS, OS)

It will occur when all evaluable patients have been performed the investigator disease assessment at 12 months from Axi-cel infusion, have died, have withdrawn consent, or are lost to follow-up, whichever occurs first.

Update of the survival endpoints analysis

It will occur when all patients having received Axi-cel infusion have been performed the investigator disease assessment at 24 months from Axi-cel infusion, have died, have withdrawn consent, or are lost to follow-up, whichever occurs first.

End of study analysis

Update of the survival endpoints will be performed at the end of study when all patients having received Axi-cel infusion have completed follow-up, have died, have withdrawn consent, or are lost to follow-up, whichever occurs first.

16 STUDY MONITORING

16.1 Responsibilities of investigators

The investigators undertake to perform the study in accordance with the Declaration of Helsinki, Good Clinical Practices-ICH E6-R2 and guidelines for the monitoring of clinical investigations, and specifically for European countries either European 2001/20/CE and 2005/28/CE directives/ Clinical trials.

The investigators ensure compliance with respect to the investigational drug schedule, visit schedule and procedures required by the study. The investigators agree to provide all information requested in the case report form in an accurate and legible manner according to instructions provided.

As may be required by the local legislation, the investigators will check that the patients are directly or indirectly affiliated to the national health insurance or coverage system if there is any.

16.2 Responsibilities of the sponsor

The sponsor (LYSARC) of this study has responsibilities towards health authorities to take all reasonable steps to ensure the proper conduct of the study as regards ethics, study adherence, integrity and validity of the data recorded on the case report forms. Thus, the main duty of the sponsor project leader and of the Sponsor clinical research support team (LYSARC) is to help the investigator maintaining a high level of ethical, scientific, technical and regulatory quality in all aspects of the study.

At regular intervals during the study, the site will be contacted, through site visits, letters or telephone calls, by a representative of the monitoring team (LYSARC) to review study progress, investigator and patient adherence to study requirements and any emergent problems.

The frequency of site contact/visits, and data monitored are defined in the monitoring plan developed specifically for the study.

16.3 Source document requirements

According to the guidelines on Good Clinical Practice, the sponsor representative will check the case report form entries against the source documents following the study monitoring plan. These personnel, bound by professional secrecy, will not disclose any personal identity or personal medical information.

16.4 Use and completion of electronic case report form (eCRF)

An electronic Case Report Form (eCRF) will be completed for each study patient. It is the investigator's responsibility to ensure the accuracy, completeness, legibility and timeliness of the data reported in the patient's eCRF available at the following website:

https://lysarc.ennov.com/EnnovClinical/login?etude=ALYCANTE

Source documentation supporting the eCRF data should indicate the paitent's participation in the study and should document the dates and details of study procedures, adverse events and patient status.

The investigator and study site staff will receive system documentation, training and support for the use of the eCRF.

Use and completion of eCRF will be carried out according to the instructions provided in the data entry and monitoring guidelines...

The system will be secured to prevent unauthorized access to the data or the system. This will include the requirement of a user ID and password to enter or change data. These user ID and password transmitted by LYSARC to study sites staff are personal and confidential. The investigator has to maintain a list of individuals who are authorized to enter or correct data. All data entry and corrections are recorded in the audit trail (date of data entry/correction, name of person, type of action).

17 ETHICAL AND REGULATORY STANDARDS

17.1 Ethical principles

This study is in accordance with the principles laid down by the 18th World Medical Assembly (Helsinki, 1964) and subsequent amendments and will be conducted according to GCP-ICH E6-R2 guidelines.

17.2 Laws and regulations

This study is performed also in accordance with applicable laws and regulations of each country involved in the trial, as well as any applicable guidelines.

All data of the patients collected by the sponsor will be pseudonymised.

17.3 Informed consent

It is the responsibility of the investigator to obtain informed consent in compliance with national requirements from each patient prior to entering the trial or, where relevant, prior to evaluating the patient's suitability for the study.

The informed consent document used by the investigator for obtaining patient's informed consent must be reviewed and approved by LYSARC prior to Ethics Review Committee submission.

As LYSARC participates to the French “Plan Cancer”, the informed consent document will be reviewed by a patient committee (*Ligue contre le cancer*).

The investigator must explain to potential patient the aims, methods, reasonable anticipated benefits and potential hazards of the trial and any discomfort it may entail. Patients will be informed that they are free not to participate in the trial and that they may withdraw consent to participate at any time. They will be told which alternative treatments are available if they refuse to take part and that such refusal will not prejudice future treatment.

The consent form will include a statement by which the patients allow the sponsor's duly authorized personnel (trial monitoring team) to have direct access to source data which supports data on the case report forms (e.g. patient's medical file, appointment books, original laboratory records, etc.).

The patient should receive a signed and dated copy of the informed consent form and patient information leaflet. The enrollment process will be documented in each patient’s medical records.

For biological studies and/or genetic studies, a specific informed consent form will be signed and dated by patients.

17.4 Ethics Review Committee and Competent Authorities submission

The Sponsor must submit this study to country Ethics Review Committee(s), and Competent Authorities. It is required to forward a copy of written signed opinions / approvals to investigators.

18 ADMINISTRATIVE PROCEDURES

18.1 Curriculum vitae

An updated signed copy of the curriculum vitae of each investigator and sub-investigator will be provided to LYSARC prior to their involvement in the study. This CV should mention the current professional address of the investigator, his education, professional experience, medical licence number, participation to clinical researches and training to Good Clinical Practices.

18.2 Confidentiality agreement

All goods, materials, information (oral or written) and unpublished documentation provided to the investigators (or any company acting on their behalf), inclusive of this study, the patient case report forms are the exclusive property of LYSARC.

They may not be given or disclosed by the investigator or by any person within his authority either in part or in totality to any unauthorized person without the prior written formal consent of LYSARC.

It is specified that the submission of this study and other necessary documentation to the Ethics Review Committee or a like body is expressly permitted, the Ethics Committee members having the same obligation of confidentiality.

The investigator shall consider as confidential and shall take all necessary measures to ensure that there is no breach of confidentiality in respect of all information accumulated, acquired or deduced in the course of the trial, other than that information to be disclosed by law.

18.3 Record retention in investigating sites

The investigator must maintain all study records, patient files and other source data for the maximum period of time permitted by the hospital, institution or private practice. The investigators will maintain locally a personal patient identification list (patient numbers with the corresponding patient names) to enable records to be identified.

However national regulations should be taken into account, the longest time having to be considered.

For trials performed in the European Union, the investigator is required to arrange for the retention of the patient identification codes for at least 25 years after the completion or discontinuation of the trial.

Any site will notify the sponsor before destroying any data or records.

18.4 Data Collection

Patients and Investigators personal data will be collected according to European General Data Protection Regulation 2016/679. Only data strictly required to reach the research objectives or to fulfill regulatory requirements will be collected and stored securely. Patient data will be pseudonymised and the correspondence patient code/patient identity will be maintained only in the patient medical record. Patients and Investigators will be informed about their rights of access, correction, opposition and how to exercise their rights before data are collected. They will also be informed about the potential re-use or transfer of their data, including transfer out of European Union, to academic or industrial partners of the Sponsor.

18.5 Ownership of data and use of the study results

The sponsor has the ownership of all data and results collected during this study. In consequence the sponsor or any third Party either appointed by the Sponsor or having concluded a specific agreement with the Sponsor, reserves the right to use the data of the present study, either in the form of case report forms (or copies of these), or in the form of a report, with or without comments and with or without analysis, in order to submit them to the health authorities of any country.

The Investigator is committed to give his support to any requests for a patent or any property title based on, or illustrated with the results of the present Study for any country.

18.6 Publication

The results of the trial will be published after complete data collection and evaluation. Partial or preliminary results can be published beforehand. Publication is to be initiated by the two coordinating investigators in charge of the study with approval of partner if applicable.

Any publication in the form of a lecture, poster or article must be basically approved by the LYSA Scientific Committee.

The authors will be proposed (according to the updated LYSA publication rules) by the coordinating investigators in charge of the study, and finally endorsed by the LYSA Steering Committee.

All study data and publications are the property of LYSA/LYSARC.

18.7 Insurance compensation

The sponsor certifies having taken out appropriate liability insurance policy which covers the Sponsor, the investigators and his co-workers and which is in accordance with the local laws and requirements. Specific statements will be contained in appendix where needed.

A certificate of insurance will be provided to the investigator in countries in which this document is required.

The Investigator(s) will remain responsible towards the Sponsor of any fault or misconduct regarding the performance of the Study.

18.8 Company audits and inspections by regulatory agencies

For the purpose of ensuring compliance with good clinical practice and regulatory agency guidelines it may be necessary to conduct a site audit or an inspection.

By signing this protocol, the investigator agrees to allow LYSARC its representative, and drug regulatory agencies to have direct access to his study records for review. These personnel, bound by professional secrecy, will not disclose any personal identity or personal medical information.

These audits involve the review of source documents supporting the adequacy and accuracy of data gathered in CRF, review of documentation required to be maintained, and checks on drug accountability.

LYSARC will in all cases help the investigator prepare for an inspection by any regulatory agency.

18.9 Clinical study report

The sponsor will declare the trial end to Competent Authorities and Ethics Committees according to local regulations.

A summary of the study results will be prepared under the responsibility of the sponsor, within one year after the end of the study and will be forwarded to each Principal Investigator, Competent Authorities and Ethics Committees and posted on Authorities' website if required by local regulations.

A suitable study report will be also prepared under the responsibility of the sponsor, within one year after the end of the study if required by local regulations.

18.10 Protocol amendments

It is specified that the appendices attached to this study and referred to in the main text of this study, form an integral part of the study.

No changes or amendments to this study may be made by the investigator or by the sponsor after the study has been agreed to and signed by both parties unless such change(s) or amendment(s) have been fully discussed and agreed upon by the coordinating investigator and LYSARC and study partner.

Approval / opinion of amendments by Ethics Review Committee(s) and/or Competent Authorities are required prior to their implementation, unless there are overriding safety reasons.

If the change or deviation increases risk to the study population, or adversely affects the validity of the clinical investigation or the patient's rights, full approval / advice must be obtained prior to implementation. For changes that do not involve increased risk or affect the validity of the investigation or the patient's rights, approval / advice may be obtained by expedited review, where applicable.

Any change agreed upon will be recorded in writing, the written amendment will be signed by the investigator and by the sponsor and the signed amendment will be appended in the Investigator Study File.

In some instances, an amendment may require a change to a consent form. The investigator must receive approval / advice of the revised consent form prior to implementation of the change. In addition, changes to the case report forms, if required, will be incorporated in the amendment.

Modifications done between different versions of the protocol are listed below:

**Version**	**Modifications**
V1.0	Initial version
V2.0	Upon request of competent authority in France (ANSM): Addition of the HCT-CI index in the study procedures of the protocol; addition of Cerebral MRI; update of exclusion criteria 17 & 19; addition of pregnancy testing at the end of relevant systemic exposure; addition of systematic neurological consultation before CAR-T cells infusion; updated of the section Adverse Events of Special Interest
V2.1	Upon request of competent authority in France (ANSM):<br>Addition of recommendation for males included in this trial to not father a child for 6 months after conditioning chemotherapy dosing or axicabtagene ciloleucel dosing, whichever is longer ; addition of close monitoring of patients, with previous HBV or HVC infection
V3.0	Addition of 20 patients ; clarification about SAEs and AEs follow-up ; clarification on biopsies and bone marrow samples

19 REFERENCES

Aaronson NK, Ahmedzai S, Bergman B, et al. The European Organization for Research and Treatment of Cancer QLQ-C30: a quality-of-life instrument for use in international clinical trials in oncology. Journal of the National Cancer Institute. Mar 03 1993;85(5):365-376.

Cazelles C, Belhadj K, Vellemans H, et al. Rituximab Plus Gemcitabine and Oxaliplatin (R-GemOx) in Refractory/Relapsed (R/R) DLBCL. a Real Life Study in Patients Ineligible for Autologous Transplantation. Blood. 2019;134(Supplement_1):4115–4115.

Chevalier J, de Pouvourville G. Valuing EQ-5D using time trade-off in France. Eur J Health Econ. févr 2013;14(1):57-66.

Crump M, Neelapu SS, Farooq U, et al. Outcomes in refractory diffuse large B-cell lymphoma: results from the international SCHOLAR-1 study. Blood. 2017;130(16):1800–1808.

Gisselbrecht C, Glass B, Mounier N, et al. Salvage Regimens With Autologous Transplantation for Relapsed Large B-Cell Lymphoma in the Rituximab Era. J. Clin. Oncol. 2010;28(27):4184–4190.

Jennison, C. and Turnbull, B.W. (2000). Group Sequential Methods with Applications to Clinical Trials. Chapman & Hall pp 235-244

Josting A, Reiser M, Rueffer U, et al. Treatment of Primary Progressive Hodgkin's and Aggressive Non-Hodgkin's Lymphoma: Is There a Chance for Cure? J. Clin. Oncol. 2000;18(2):332–332.

D.W. Lee et al. ASTCT Consensus Grading for Cytokine Release Syndrome and Neurologic Toxicity Associated with Immune Effector Cells. Biol Blood Marrow Transplant 25 (2019) 625 – 638

Locke FL, Ghobadi A, Lekakis LJ, et al. Outcomes by prior lines of therapy (LoT) in ZUMA-1, the pivotal phase 2 study of axicabtagene ciloleucel (Axi-Cel) in patients (Pts) with refractory large B cell lymphoma. J. Clin. Oncol. 2018;36(15_suppl):3039–3039.

Locke FL, Ghobadi A, Jacobson CA, et al. Long-term safety and activity of axicabtagene ciloleucel in refractory large B-cell lymphoma (ZUMA-1): a single-arm, multicentre, phase 1–2 trial. Lancet Oncol. 2019;20(1):31–42.

Mounier N, El Gnaoui T, Tilly H, et al. Rituximab plus gemcitabine and oxaliplatin in patients with refractory/relapsed diffuse large B-cell lymphoma who are not candidates for high-dose therapy. A phase II Lymphoma Study Association trial. Haematologica. 2013;98(11):1726–1731.

Neelapu SS, Locke FL, Bartlett NL, et al. Axicabtagene ciloleucel CAR T-cell therapy in refractory large B-Cell lymphoma. N. Engl. J. Med. 2017;377(26):2531–2544.

Neelapu SS, Jacobson CA, Oluwole OO, et al. Outcomes of older patients in ZUMA-1, a pivotal study of axicabtagene ciloleucel in refractory large B-cell lymphoma. Blood. 2020;

Pasquini MC, Locke FL, Herrera AF, et al. Post-Marketing Use Outcomes of an Anti-CD19 Chimeric Antigen Receptor (CAR) T Cell Therapy, Axicabtagene Ciloleucel (Axi-Cel), for the Treatment of Large B Cell Lymphoma (LBCL) in the United States (US). Blood. 2019;134(Supplement_1):764–764.

Philip T, Guglielmi C, Hagenbeek A, et al. Autologous Bone Marrow Transplantation as Compared with Salvage Chemotherapy in Relapses of Chemotherapy-Sensitive Non-Hodgkin's Lymphoma. N. Engl. J. Med. 1995;333(23):1540–1545.

Pickard AS, Neary MP, Cella D. Estimation of minimally important differences in EQ-5D utility and VAS scores in cancer. Health Qual Life Outcomes 2007; 5: 70.

Sorror ML, Maris MB, Storb R, et al. Hematopoietic cell transplantation (HCT)-specific comorbidity index: a new tool for risk assessment before allogeneic HCT. Blood. 2005;106(8):2912–2919.

Sorror ML, Logan BR, Zhu X, et al. Prospective Validation of the Predictive Power of the Hematopoietic Cell

Transplantation Comorbidity Index: A Center for International Blood and Marrow Transplant Research Study. Biol. Blood Marrow Transplant. 2015;21(8):1479–1487.

van de Poll-Franse L, Oerlemans S, Bredart A, Kyriakou C, Sztankay M, Pallua S, Daniëls L, Creutzberg C, Cocks K, Malak S, Caocci G, Molica S, Chie W, Efficace F; EORTC Quality of Life Group. International development of four EORTC disease-specific quality of life questionnaires for patients with Hodgkin lymphoma, high- and low-grade non-Hodgkin lymphoma and chronic lymphocytic leukaemia. Qual Life Res. 2018 Feb;27(2):333-345. doi: 10.1007

20 APPENDICES

20.1 Appendix 01: Study Design

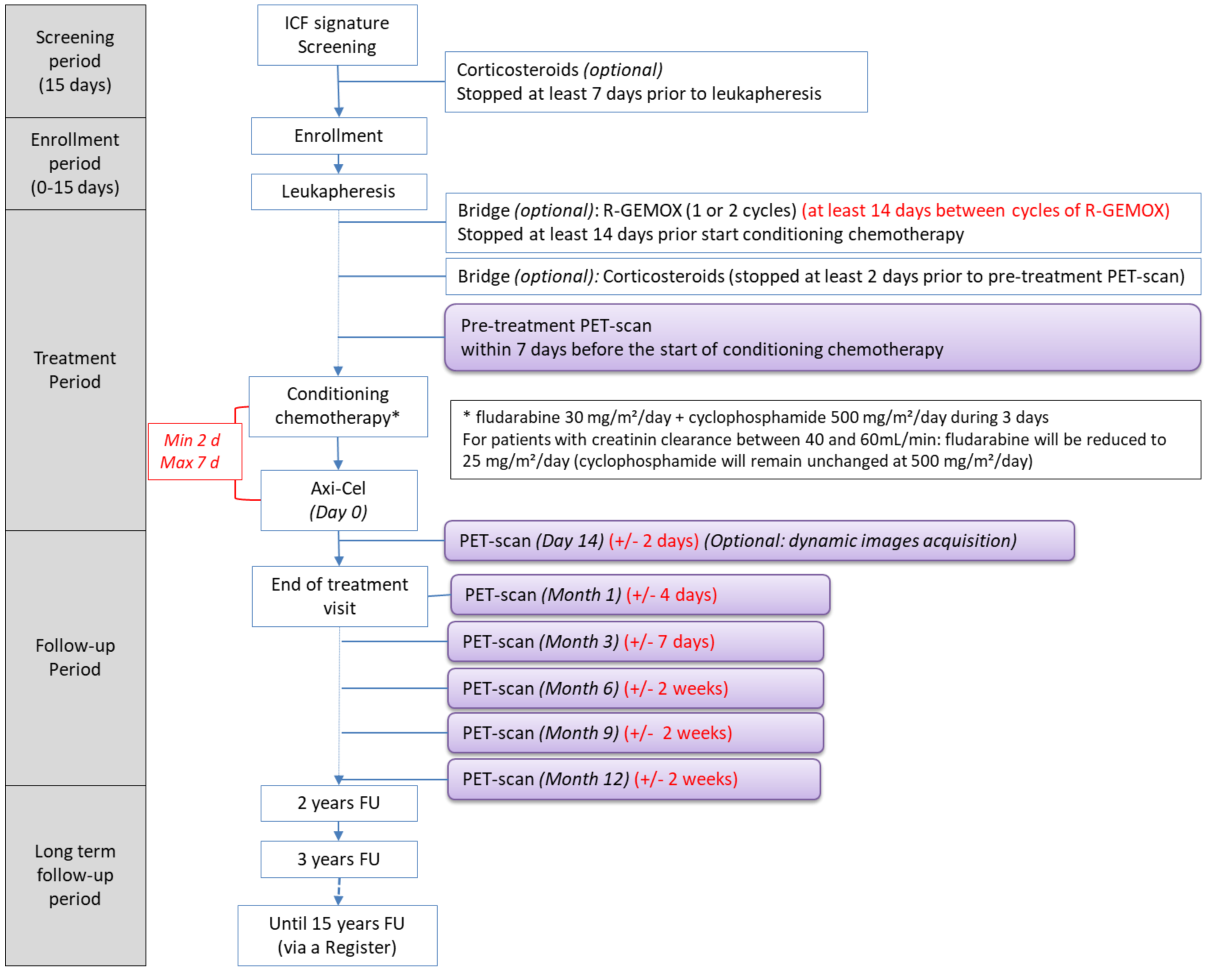

20.2 Appendix 02: Schedule of Assessments (study flow-chart)

	Screening	Enrollment Leukapheresis	Conditioning chemotherapy			Administration Axi-Cel and FU post administration		Follow-up								Relapse/ progression
Date (days or months)	*Within 15 days of enrollment*	*2-4 weeks before cond chemotherapy*	*-5*	*-4*	*-3*	*Day 0*	*1 to 10 (until end of hospitalization)*	*D14 (+/- 2 d)*	*Month 1 (+/- 4 d)*	*M3 (+/- 7 d)*	*M6 (+/- 14 d)*	*M9 (+/- 14 d)*	*M12 (+/- 14 d)*	*M24 (+/- 14 d)*	*M36 (+/- 14 d)*	
Written informed consent	X															
Inclusion/exclusion criteria review	X	X	X			X										
Patient characteristics (a)	X															
Clinical examination (b)	X	X	X			X	X		X	X	X	X	X			
Vital signs (heart rate, blood pressure and body temperature and oxygen saturation)	X	X	X	X	X	X	X (k)		X (k)							
Weight		X	X			X	X		X	X	X	X	X			
ECG	X															
Echocardiography	X															
Fresh tumor biopsy	X (l)						X (l)									X
HIV, HBV, HCV, syphilis serologies	X															
Blood cell counts (c)	X	X	X	X	X	X	X	X	X	X	X	X	X			
Samples for biological banking and biological analysis	See Appendix 03															
Pregnancy test	X										X					
Biochemical tests (d)	X	X	X	X	X	X	X		X	X	X	X	X			
LDH, ferritin	X	X	X	X	X	X	X	X	X	X	X	X	X			
Coagulation (fibrinogen, PT, aPTT)	X	X	X	X	X	X	X (m)									
CRP	X	X	X	X	X	X	X	X								
Gamma globulins			X						X	X	X	X	X			
Leukapheresis		X														
PET Scan (e)	X (e)	X (e)						X	X	X	X	X	X			X
Cerebral MRI and neurological consultation (f)	X															

	Screening	Enrollment Leukapheresis	Conditioning chemotherapy			Administration Axi-Cel and FU post administration		Follow-up								Relapse/ progression
Date (days or months)	*Within 15 days of enrollment*	*2-4 weeks before cond chemotherapy*	*-5*	*-4*	*-3*	*Day 0*	*1 to 10 (until end of hospitalization)*	*D14 (+/- 2 d)*	*Month 1 (+/- 4 d)*	*M3 (+/- 7 d)*	*M6 (+/- 14 d)*	*M9 (+/- 14 d)*	*M12 (+/- 14 d)*	*M24 (+/- 14 d)*	*M36 (+/- 14 d)*	
Bone marrow biopsy and aspirate (g)	X								X							
Lumbar puncture (h)	X						X (h)									
Quality of life questionnaires	X		X						X	X	X		X	X	X	
Bridging (optional) (i)		X														
Fludarabine and cyclophosphamide administration			X	X	X											
Concomitant treatment (j)		X	X	X	X	X	X	X	X							
Survival Status														X	X	
Toxicities		Continuous report								AESI, related SAEs						

(a): Age, gender, weight, height, BSA, relevant medical history, B symptoms, history of the NHL
(b) including ECOG performance status, IPI score (at screening only), Ann Arbor staging (at screening only), HCT-CI score (screening only)
(c): Blood cell count will include hemoglobin, platelets, white blood cell (WBC) count with differential, absolute neutrophil count (ANC), and absolute lymphocyte count (ALC). In addition, B-cells and T-cells (CD4 and CD8) will be quantified at Day 0, and Month 1, 3, 6, 9 and 12.
(d): Biochemical tests will include sodium, potassium, glucose, serum creatinin, creatinine clearance according to MDRD/Cockcroft-Gault formula, AST, ALT, total bilirubin and alkaline phosphatases
(e): At screening: availability of baseline PET-scan (done before study participation to document the relapsed or refractory disease after first-line chemoimmunotherapy) should be checked and should be provided.
Pre-treatment PET-scan: should be done within 7 days before the start of conditioning chemotherapy. At follow-up, PET-CT should be performed at day 14 and month 1, 3, 6, 9 and 12 after Axi-Cel infusion (day 0)
(f): Neurological consultation: to be done prior Axi-cel infusion
(g): Bone marrow biopsy and aspirate within 28 days of enrollment should be performed in patients with unexplained cytopenias or suspicion of bone marrow involvement. At M1 in patients with evidence of baseline bone marrow involvment or in case of persistent unexplained cytopenias
(h): if clinically indicated – mandatory if prior history of CNS involvement by lymphoma. Additionnal CSFsamples will be collected and banked from patients who experienced Grade ≥ 2 ICANS (ASTCT) to enable evaluation of inflammatory cytokine/chemokine levels and presence of CAR-T cells. As applicable, lymphocyte populations residing in the CSF, or other biological samples, may also be monitored for the prupose of understanding the safety profile of axicabtagene ciloleucel.
(i): Bridging therapy (optional) administered after leukapheresis and completed prior to initiating conditioning chemotherapy.
R-GEMOX (1 or 2 cycles) (at least 14 days between cycles of R-GEMOX) - Stopped at least 14 days prior to start of conditioning chemotherapy
Or Steroids (stopped at least 2 days prior to pre-treatment PET-scan)
(j): Concomitant therapy consists of any therapy used by a patient in addition to protocol-mandated treatment from leukapheresis to end of treatment visit
Before enrollment / leukapheresis - optional steroids: must be discontinued at least 7 days prior leukapheresis
(k): at least every 8 hours during hospitalization
At Month 1: only heart rate, blood pressure and body temperature
(l): if no achival tumor tissue is available for central pathology review and exploratory/ancillary studies: mandatory at screening
Tumor biopsy: Optional at Day 2 (+/- 1 day)
(m): twice of week at minimum until end of hospitalization (additional assessment at investigator's discretion)

20.3 Appendix 03: Schedule of biological banking samples

Material		Sampling	Schedule																	if ICANS ≥ grade 2 (ASTCT)	On site preparation	Destination
			At screening	At leukapherisis	D-5	D0 (before CAR-T)	D1	D2 +/-1	D3 +/-1	D5 +/-1	D7	D10 +/-2	D14	M1	M3	M6	M9	M12	Prog/Relapse			
Archival tumor tissue (diag or relapse)	FFPE	Archival block	×																		FFPE inclusion	LYSA-P/Toulouse, FR
	FFPE	≥ 10 unstained slides	×																		FFPE inclusion	LYSA-P/Toulouse, FR
	FFPE	Immunostained slides	×																		FFPE inclusion	LYSA-P/Toulouse, FR
	FROZEN	Frozen biopsy	×																		Freezing	LYSA-P/Toulouse, FR
TUMOR biopsy (a)	FFPE	1st and 2nd core needle biopsy	×					×											×		FFPE inclusion	LYSA-P/Toulouse, FR
	FROZEN	3rd core needle biopsy	×					×											×		Flash freezing	LYSA-P/Toulouse, FR
	CRYOSTOR® FROZEN	4th core needle biopsy	×					×											×		Cryostor	LYSA-P/Rennes, FR
	CRYOSTOR® FROZEN	5th core needle biopsy	×																		Cryostor	LYSA-P/Rennes, FR
	CRYOSTOR® FROZEN	Fine needle aspiration	×					×											×		Cryostor	LYSA-P/Rennes , FR
BLOOD	frozen cells (c)	12mL, CPT heparinized			×	×				×	×	×	×	×	×	×	×	×	×		Immediate shipment	Kite, USA
	serum (c)	5mL SST tube (gold top)		× (b)	×	×	×		×	×	×	×	×	×	×						Gathering at the end of study	On site --> Kite, USA
	cells	Fresh blood 16ml, heparin tubes		× (b)	×	×					×	×	×	×	×	×	×	×	×		Immediate shipment	Rennes, FR
	DNA	Fresh blood 9mL Strek® tube		× (b)	×	×							×	×	×	×	×	×	×		Immediate shipment	Rennes, FR
PRODUCT	final product formulation	CAR-T cells products not infused				×																On site --> Rennes, FR
CSF	frozen	Cells, cytokines	× (d)																	×	Centrifuge and freezing	On site, --> Rennes, FR
Maximum blood volume (mL)				30	42	42	5		5	17	33	33	42	42	42	37	37	37	37			

(a) Tumor biopsy: mandatory if no archival tumor tissue available, highly recommended otherwise

(b) +/- 2 days is allowed if timepoint does not occur on a working day

(c) Analysed by Kite. PBMC for cellular kinetics of CAR-T cells and serum for cytokines and RCR (replication-competent retrovirus).

(d) CSF: Mandatory if prior history of CNS involvement by lymphoma or if clinically indicated (suspicion of CNS involement) / otherwise optional

A missing timepoint does not cancel following samples.

20.4 Appendix 04: Body Surface Area calculation

The algorithm to be used in this study is Mosteller formula (1987):
**BSA = √ [(Height (cm) x Weight (kg))/3600]**

20.5 Appendix 05: Performance Status Criteria

The following table presents the ECOG performance status scale:

ECOG Performance Status Scale	
**Grade**	**Description**
**0**	Normal activity. Fully active, able to carry on all pre-disease performance without restriction
**1**	Symptoms but ambulatory. Restricted in physically strenuous activity, but ambulatory and able to carry out work of a light or sedentary nature (eg, light housework, office work).
**2**	In bed <50% of the time. Ambulatory and capable of all self-care, but unable to carry out any work activities. Up and about more than 50% of waking hours.
**3**	In bed >50% of the time. Capable of only limited self-care, confined to bed or chair more than 50% of waking hours.
**4**	100% bedridden. Completely disabled. Cannot carry on any self-care. Totally confined to bed or chair.

Source: Oken MM, Creech RH, Tormey DC, Horton J, Davis TE, McFadden ET et al. Toxicity and response criteria of the Eastern Cooperative Oncology Group. Am J Clin Oncol 1982; 5 (6):649-55.

DocuSign Envelope ID: 4C1EC433-1B7A-4CD2-B02E-B91666107FC1

20.6 Appendix 06: Ann Arbor staging

Stage I:

- **I**: Involvement of a single lymph node region
- **IE**: Localized involvement of a single extralymphatic organ or site.

Stage II:

- **II**: Involvement of 2 or more lymph node regions on the same side of the diaphragm
- **IIE**: Localized involvement of a single associated extralymphatic organ or site and its regional lymph nodes with or without other lymph node regions on the same side of the diaphragm

Stage III:

- **III**: Involvement of lymph node regions on both sides of the diaphragm
- **IIIE**: Involvement of lymph node regions on both sides of the diaphragm accompanied by localized involvement of an extralymphatic organ or site
- **IIIS**: Involvement of lymph node regions on both sides of the diaphragm accompanied by involvement of the spleen
- **IIIS+E**: Both IIIS+IIIE

Stage IV:

- **IV**: Disseminated (multifocal) involvement of 1 or more extralymphatic sites with or without associated lymph node involvement or isolated extralymphatic organ involvement with distant (non regional) nodal involvement
- **IVE**: Extranodal lymphoid malignancies arise in tissues separate from, but near, the major lymphatic aggregates.

Source: American Joint Committee on Cancer. Non Hodgkin's lymphoma. In: AJCC Staging Manual. 5th ed. Philadelphia, PA: Lippincott-Raven;1997:289-294.

20.1 Appendix 07: International Prognostic Index (IPI)

For IPI, score 1 point for each of the following risk factors:

Age	> 60
Lactate dehydrogenase (LDH) level	> normal
Ann Arbor stage	III-IV
Performance status (PS)	2-4
Extra-nodal involvement	more than 1 site

**RISK GROUPS**	**Number of Factors**
Low	0-1
Low intermediate	2
High intermediate	3
High	4-5

Source: The international Non Hodgkin Lymphoma prognostic factor project. A predictive model for aggressive non Hodgkin lymphoma. New England Journal of Medicine 1993; 329:987-994.

DocuSign Envelope ID: 4C1EC433-1B7A-4CD2-B02E-B91666107FC1

20.2 Appendix 08: Quality of life questionnaires

EORTC QLQ-C30 (version 3)

We are interested in some things about you and your health. Please answer all of the questions yourself by circling the number that best applies to you. There are no "right" or "wrong" answers. The information that you provide will remain strictly confidential.

Please fill in your initials: αααα
Your birthdate (Day, Month, Year): βδβδβχχδ
Today's date (Day, Month, Year): 31 βδβδβχχδ

		NOT AT ALL	A LITTLE	QUITE A BIT	VERY MUCH
1.	Do you have any trouble doing strenuous activities, like carrying a heavy shopping bag or a suitcase?	1	2	3	4
2.	Do you have any trouble taking a long walk?	1	2	3	4
3.	Do you have any trouble taking a short walk outside of the house?	1	2	3	4
4.	Do you need to stay in bed or a chair during the day?	1	2	3	4
5.	Do you need help with eating, dressing, washing yourself or using the toilet?	1	2	3	4

During the past week:

		NOT AT ALL	A LITTLE	QUITE A BIT	VERY MUCH
6.	Were you limited in doing either your work or other daily activities?	1	2	3	4
7.	Were you limited in pursuing your hobbies or other leisure time activities?	1	2	3	4
8.	Were you short of breath?	1	2	3	4
9.	Have you had pain?	1	2	3	4
10.	Did you need to rest?	1	2	3	4
11.	Have you had trouble sleeping?	1	2	3	4
12.	Have you felt weak?	1	2	3	4
13.	Have you lacked appetite?	1	2	3	4
14.	Have you felt nauseated?	1	2	3	4
15.	Have you vomited?	1	2	3	4
16.	Have you been constipated?	1	2	3	4

Please go on to the next page

DURING THE PAST WEEK:	NOT AT ALL	A LITTLE	QUITE A BIT	VERY MUCH
17. Have you had diarrhea?	1	2	3	4
18. Were you tired?	1	2	3	4
19. Did pain interfere with your daily activities?	1	2	3	4
20. Have you had difficulty in concentrating on things, like reading a newspaper or watching television?	1	2	3	4
21. Did you feel tense?	1	2	3	4
22. Did you worry?	1	2	3	4
23. Did you feel irritable?	1	2	3	4
24. Did you feel depressed?	1	2	3	4
25. Have you had difficulty remembering things?	1	2	3	4
26. Has your physical condition or medical treatment interfered with your <u>family</u> life?	1	2	3	4
27. Has your physical condition or medical treatment interfered with your <u>social</u> activities?	1	2	3	4
28. Has your physical condition or medical treatment caused you financial difficulties?	1	2	3	4

For the following questions please circle the number between 1 and 7 that best applies to you

29. How would you rate your overall <u>health</u> during the past week?

1	2	3	4	5	6	7
Very poor						Excellent

30. How would you rate your overall <u>quality of life</u> during the past week?

1	2	3	4	5	6	7
Very poor						Excellent

EQ-5D-5L

Under each heading, please tick the ONE box that best describes your health TODAY.

MOBILITY

I have no problems in walking about ❑
I have slight problems in walking about ❑
I have moderate problems in walking about ❑
I have severe problems in walking about ❑
I am unable to walk about ❑

SELF-CARE

I have no problems washing or dressing myself ❑
I have slight problems washing or dressing myself ❑
I have moderate problems washing or dressing myself ❑
I have severe problems washing or dressing myself ❑
I am unable to wash or dress myself ❑

USUAL ACTIVITIES *(e.g. work, study, housework, family or leisure activities)*

I have no problems doing my usual activities ❑
I have slight problems doing my usual activities ❑
I have moderate problems doing my usual activities ❑
I have severe problems doing my usual activities ❑
I am unable to do my usual activities ❑

PAIN / DISCOMFORT

I have no pain or discomfort ❑
I have slight pain or discomfort ❑
I have moderate pain or discomfort ❑
I have severe pain or discomfort ❑
I have extreme pain or discomfort ❑

ANXIETY / DEPRESSION

I am not anxious or depressed ❑
I am slightly anxious or depressed ❑
I am moderately anxious or depressed ❑
I am severely anxious or depressed ❑
I am extremely anxious or depressed ❑

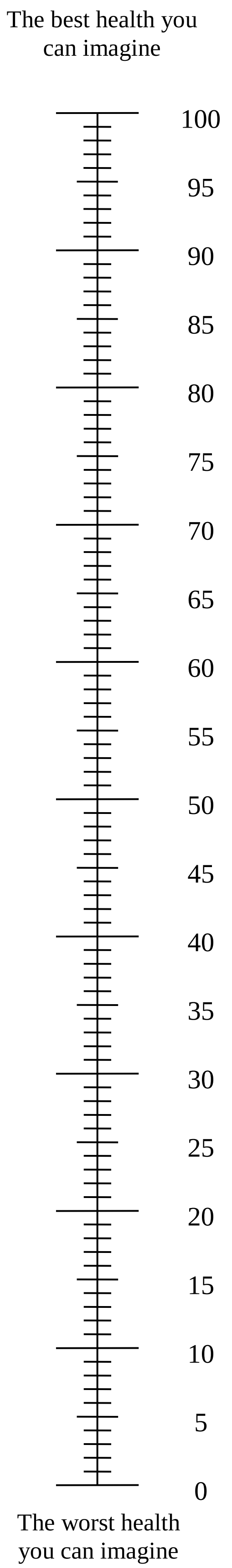

- We would like to know how good or bad your health is TODAY.
- This scale is numbered from 0 to 100.
- 100 means the <u>best</u> health you can imagine.
  0 means the <u>worst</u> health you can imagine.
- Please mark an X on the scale to indicate how your health is TODAY.
- Now, write the number you marked on the scale in the box below.

YOUR HEALTH TODAY = ☐

EORTC QLQ-NHL-HG29

Patients sometimes report that they have the following symptoms or problems. Please indicate the extent to which you have experienced these symptoms or problems during the past week. Please answer by circling the number that best applies to you.

During the past week:	Not at All	A Little	Quite a Bit	Very Much
31. Have you had muscle weakness?	1	2	3	4
32. Have you had aches or pains in your muscles or joints?	1	2	3	4
33. Have you had aches or pains in your bones?	1	2	3	4
34. Have you had a dry cough?	1	2	3	4
35. Have you had a dry mouth?	1	2	3	4
36. Have you had problems with your sense of taste?	1	2	3	4
37. Have you felt ill or unwell?	1	2	3	4
38. Have you had tingling hands or feet?	1	2	3	4
39. Have you had numbness in your fingers or toes?	1	2	3	4

During the past week:	Not at All	A Little	Quite a Bit	Very Much
40. Have you had shortness of breath on exertion?	1	2	3	4
41. Have you felt you had setbacks in your physical condition?	1	2	3	4
42. Have you had a lack of energy?	1	2	3	4
43. Have you felt drowsy?	1	2	3	4
44. Have you had sudden tiredness?	1	2	3	4

During the past week:	Not at All	A Little	Quite a Bit	Very Much
45. Have you had mood changes?	1	2	3	4
46. Have you felt a lack of confidence in your body?	1	2	3	4
47. Have you been dissatisfied with how your body functions?	1	2	3	4
48. Have you had difficulty accepting limitations due to the disease?	1	2	3	4

Please go on to next page

**During the past 4 weeks:**	**Not at All**	**A Little**	**Quite a Bit**	**Very Much**
49. Have you worried about picking up an infection?	1	2	3	4
50. Have you worried about your health in the future?	1	2	3	4
51. Have you worried about recurrence of your disease?	1	2	3	4
52. Have you worried about becoming chronically ill?	1	2	3	4
53. Have you worried about becoming dependent on others?	1	2	3	4
54. Have you worried about getting another type of cancer?	1	2	3	4
55. Have you worried about your treatment causing future health problems?	1	2	3	4
56. Have you worried about damage to your heart and blood vessels?	1	2	3	4
57. If applicable: Have you had problems at your work or place of study due to the disease?	1	2	3	4
58. If applicable: Have you worried about not being able to continue working or your education?	1	2	3	4
59. If applicable: Have you been concerned about your ability to have children?	1	2	3	4

20.3 Appendix 09: Hematopoietic Cell Transplantation-specific Comorbidity Index (HCT-CI)

Adapted from Sorror et al. Blood 2005

Comorbidity	Definitions of comorbidities HCT-CI	HCT-CI weighted scores
Arrhythmia[1]	Atrial fibrillation or flutter, sick sinus syndrome, or ventricular arrhythmias	1
Cardiac	Coronary artery disease [2], congestive heart failure, myocardial infarction, or EF ≤ 50%	1
Inflammatory bowel disease[3]	Crohn disease or ulcerative colitis	1
Diabetes[4]	Requiring treatment with insulin or oral hypoglycemic but not diet alone	1
Cerebrovascular disease[5]	Transient ischemic attack or cerebrovascular accident	1
Psychiatric disturbance[6]	Depression or anxiety requiring psychiatric consult or treatment	1
Hepatic, mild	Chronic hepatitis, bilirubin > upper reference limit to 1.5 x ULN, or<br>AST/ALT > upper reference limit to 2.5 x ULN	1
Obesity	Patients with a body mass index > 35 kg/m^2	1
Infection	Requiring continuation of antimicrobial treatment after day 0	1
Rheumatologic[7]	SLE, RA, polymyositis, mixed CTD, or polymyalgia rheumatica	2
Peptic ulcer	Requiring treatment	2
Moderate/severe renal[8]	Serum creatinine > 2 mg/dL, on dialysis, or prior renal transplantation	2
Moderate pulmonary[9]	DLco and/or FEV1 66%-80% or dyspnea on slight Activity	2
Prior solid tumor[10]	Treated at any time point in the patient's past history, excluding nonmelanoma skin cancer	3
Heart valve disease[11]	Except mitral valve prolapse	3
Severe pulmonary	DLco and/or FEV1 ≤ 65% or dyspnea at rest or requiring oxygen	3
Moderate/severe hepatic	Liver cirrhosis, bilirubin > 1.5 x ULN, or AST/ALT > 2.5<br>x ULN	3

1. Arrhythmia is to be scored only if **required specific treatment** at any time point in the patient's past medical history. Lack of any details on treatment for a prior arrhythmia **should not preclude** scoring the arrhythmia as comorbidity. However, if careful review of the medical chart or evaluation of patient's medical history raises doubt whether a treatment was given or not for an arrhythmia, **no score** should be assigned.
2. One or more vessel-coronary artery stenosis requiring medical treatment, stent, or bypass
3. Inflammatory bowel disease comorbidity is to be scored only if requiring treatment at study entry.
4. Diabetes or steroid-induced hyperglycemia is to be scored only if required continuous treatment with insulin or oral hypoglycemic drugs within the 4 weeks period prior to the start of conditioning regimen. Patients treated with diet only are not to be scored for this comorbidity. Patients who previously received treatment for diabetes or steroid-induced hyperglycemia but stopped these medication 4 weeks before the start of conditioning regimen are not to be scored for this comorbidity.
5. Cerebro-vascular disease is to be scored if happened at any time point in the past medical history.

6. Psychiatric disturbance comorbidity is to be scored if required continuous treatment during the 4 weeks period prior to start of conditioning regimen. Patients who are receiving only "as needed" medications for any of the above disorders are not to be scored for this comorbidity.
7. Rheumatologic comorbidity is to be scored only if required specific treatment at study entry.
8. Renal transplantation that happened at any time point in the patient's past medical history is to be scored for renal comorbidity.
9. Shortness of breath has to be assessed during a clinic visit within two weeks prior to the start of conditioning regimen.The requirement for intermittent or continuous oxygen supplementation is to be scored if occurred during the entire 4 weeks duration prior to the start of conditioning regimen. Among patients who receive a bronchodilator challenge during pulmonary function tests, only the pre-bronchodilator values are to be considered for evaluation of pulmonary comorbidity.
10. Patients with prior solid tumor that never required specific treatment **are not** to be scored for this comorbidity.
11. Impairment of heart valve function, whether stenosis or insufficiency, has to be of at least a moderate degree to be considered for this comorbidity.

20.4 Appendix 10: Response Criteria for Lymphoma – Lugano Classification

Revised Criteria for Response Assessment		
**Response and Site**	**PET-CT–Based Response**	**CT-Based Response**
**Complete**	**Complete metabolic response**	**Complete radiologic response (all of the following)**
Lymph nodes and extralymphatic sites	Score 1, 2, or 3_ with or without a residual mass on 5 Point Scale†<br>It is recognized that in Waldeyer's ring or extranodal sites with high physiologic uptake or with activation within spleen or marrow (eg, with chemotherapy or myeloid colony-stimulating factors), uptake may be greater than normal mediastinum and/or liver. In this circumstance, complete metabolic response may be inferred if uptake at sites of initial involvement is no greater than surrounding normal tissue even if the tissue has high physiologic uptake	Target nodes/nodal masses must regress to ≤ 1.5 cm in LD<br>No extralymphatic sites of disease
Non-measured lesion	Not applicable	Not applicable
Organ enlargement	Not applicable	Regress to normal
New lesions	None	None
Bone marrow	No evidence of FDG-avid disease in marrow	Normal by morphology; if indeterminate, IHC negative
**Partial**	**Partial metabolic response**	**Partial remission (all of the following)**
Lymph nodes and extralymphatic sites	Score 4 or 5† with reduced uptake compared with baseline and residual mass(es) of any size<br>At interim, these findings suggest responding disease<br>At end of treatment, these findings indicate residual disease	≥ 50% decrease in SPD of up to 6 target measurable nodes and extranodal sites<br>When a lesion is too small to measure on CT, assign 5 mm x 5 mm as the default value<br>When no longer visible, 0 x 0 mm<br>For a node > 5 mm x 5 mm, but smaller than normal, use actual measurement for calculation
Non-measured lesion	Not applicable	Absent/normal, regressed, but no increase
Organ enlargement	Not applicable	Spleen must have regressed by > 50% in length beyond normal
New lesions	None	None
Bone marrow	Residual uptake higher than uptake in normal marrow but reduced compared with baseline (diffuse uptake compatible with reactive changes from chemotherapy allowed). If there are persistent focal changes in the marrow in the context of a nodal response, consideration should be given to further evaluation with MRI or biopsy or an interval scan	Not Applicable
**No Response or stable disease**	**No metabolic response**	**Stable Disease**
Target nodes/nodal masses, extranodal lesions	Score 4 or 5 with no significant change in FDG uptake from baseline at interim or end of treatment	< 50% decrease from baseline in SPD of up to 6 dominant, measurable nodes and extranodal sites; no criteria for progressive disease are met
Non-measured lesion	Not applicable	No increase consistent with progression
Organ enlargement	Not applicable	No increase consistent with progression
New lesions	None	None
Bone marrow	No change from baseline	Not applicable
**Progressive disease**	**Progressive Metabolic Response**	**Progressive disease at least 1 of the following**
Individual target nodes/nodal masses	Score 4 or 5 with an increase in intensity of uptake from baseline and/or	PPD progression

Extranodal lesions	New FDG-avid foci consistent with lymphoma at interim or end-of-treatment assessment	An individual node/lesion must be abnormal with:<br>LDi > 1.5 cm and<br>Increase by ≥ 50% from PPD nadir and<br>An increase in LDi or SDi from nadir<br>0.5 cm for lesions ≤ 2 cm<br>1.0 cm for lesions > 2 cm<br>In the setting of splenomegaly, the splenic length must increase by > 50% of the extent of its prior increase beyond baseline (eg, a 15-cm spleen must increase to > 16 cm). If no prior splenomegaly, must increase by at least 2 cm from baseline<br>New or recurrent splenomegaly
Nonmeasured lesion	None	New or clear progression of preexisting nonmeasured lesions
New lesions	New FDG-avid foci consistent with lymphoma rather than another etiology (eg, infection, inflammation). If uncertain regarding etiology of new lesions, biopsy or interval scan may be considered	Regrowth of previously resolved lesions<br>A new node > 1.5 cm in any axis<br>A new extranodal site >1.0 cm in any axis; if < 1.0 cm in any axis, its presence must be unequivocal and must be attributable to lymphoma<br>Assessable disease of any size unequivocally attributable to lymphoma
Bone Marrow	New or recurrent FDG-avid foci	New or recurrent involvement

Source: Bruce D. Cheson, Richard I. Fisher, Sally F. Barrington, Franco Cavalli, Lawrence H. Schwartz, Emanuele Zucca, and T. Andrew Lister. J Clin Oncol 2014;32(27):3059-68.

20.5 Appendix 11: Deauville criteria for PET analysis

Deauville criteria is a scoring system and it will be used for intermediate evaluation (at the end of cycles 2, 4 and 6) and during maintenance period.

We will use a 5 points scale (adapted from the Deauville workshop in Leukemia & Lymphoma, August 2009; 50(8): 1257–1260), with new modifications (mainly 4 and 5 scales).

It includes visual and quantitative analysis.

- **1**. No uptake.
- **2**. Uptake < mediastinum.
- **3**. Uptake > mediastinum but < liver.
- **4**. Uptake moderately more than liver uptake, at any site.
- **5**. Markedly increased uptake at any site and/or new sites of disease.

TEP positive is defined by scale level 4 and 5 (as described above)

TEP negative is defined by scale level 1, 2 and 3.

20.6 Appendix 12: PET SCANs

FDG PET/CT imaging should follow the standardized protocol elaborated by EANM organization. In particular, careful attention should be paid to the scheduled protocol (1 hour between FDG administration and PET acquisition), the glycemic status and, for each patient, unchanged technical parameters of acquisition.

Source: FDG PET/CT: EANM procedure guidelines for tumour imaging: version 2.0 Ronald Boellaard et al. November 2014 Eur J Nucl Med Mol Imaging DOI 10.1007 / s00259-014-2961-x (available on http://www.eanm.org/publications/guidelines/2015_GL_PET_CT_TumorImaging_V2.pdf)

20.6.1 Timing of FDG PET scans

- A Pre treatment FDG PET/CT (PET-PreCART) is mandatory. Pre-treatment PET-CT will be performed within 7 days before the start of conditioning chemotherapy.
- A FDG PET/CT (PET_D14) has to be performed 14 days (+/- 2 days) after CAR-T injection. A dynamic images acquisition with Siemens Vision camera has described in Appendix 13 can be performed by sites with this camera
- A FDG PET/CT (PET_M1) has to be performed 1 month after CAR-T injection.
- A FDG PET/CT (PET_M3) has to be performed 3 month after CAR-T injection.
- A FDG PET/CT (PET_M6) has to be performed 6 month after CAR-T injection.
- A FDG PET/CT (PET_M9) has to be performed 9 month after CAR-T injection.
- A FDG PET/CT (PET_M12) has to be performed 12 month after CAR-T injection.

20.6.2 Patient preparation

- Patients are not allowed to consume any food or sugar for at least 6 h prior to the start of the PET study (i.e. with respect to time of injection of FDG).
- Adequate pre-hydration is important to ensure a sufficiently low FDG concentration of FDG in urine (fewer artifacts) and for radiation safety reasons (for example, 1 l of water in the 2 h prior to injection).
- Parental nutrition and intravenous fluids containing glucose should be discontinued at least 4 h before the PET/CT examination. In addition, the infusion used to administer intravenous pre-hydration must not contain any glucose.
- During the injection of FDG and the subsequent uptake phase the patient should remain seated or recumbent and silent to minimize FDG uptake in muscles.
- Blood glucose level must be measured prior to administering FDG:

- If plasma glucose level is <7 mmol/l (or <120 mg/dl) the FDG PET study can be performed (recommended)
- But if plasma glucose level is ≥7 mmol/l (or ≥ 120 mg/dl) and ≤ 8 mmol/l (or ≤150mg/dl) the FDG PET study can be performed.
- If plasma glucose level is >8 mmol/l (or >150 mg/dl) the FDG PET study must be rescheduled or the patient excluded depending on the patient circumstances

- The following recommendations apply to patients with diabetes mellitus:

- type II diabetes mellitus (controlled by oral medication)
    - the PET study should preferably be performed in the late morning
    - patients must comply with the fasting rules indicated above
    - patients continue to take oral medication to control their blood sugar.
- type I diabetes mellitus and insulin-dependent type II diabetes mellitus
    - ideally, an attempt should be made to achieve normal glycemic values prior to the PET study, in consultation with the patient and his/her attending medical doctor
    - the PET study should be scheduled for late morning

- the patient should eat a normal breakfast at 7.00 a.m. and inject the normal amount of insulin.

- Height and body weight must be determined at first scan and weight must be measured directly prior to each PET study because body weight often changes during course of disease.

20.6.3 PET scanner technical requirements

- FDG-PET scanning should be performed with a combined PET/CT for an improved data interpretation. Unless specifically excluded for particular protocols.
- Each patient is preferably scanned on the same camera for baseline, intermediate and final study.

20.6.4 PET acquisition and reconstruction

- The 18F-FDG injected activity will be defined according to on-site rules but should be >3.5 MBq/kg (or recommended activity for more recent PET-CT technology (TOF).

It is especially important to ensure that the time between tracer administration and starting of PET acquisition will be the same (± 5 min) at each of the 7 PET scans

- The patient should be positioned with the arm elevated over the head and PET acquisition should cover at least from the mid-femora to the external auditory meatus.
- A whole body acquisition with attenuation correction (non contrast-enhanced CT) and with emission scans of at least 2 minutes per bed position (or less for more recent PET-CT technology (TOF) is started 60+/-10 minutes after FDG injection, starting from groin up to the head.
- FDG PET/CT imaging should follow the standardized protocol elaborated by EANM organization. In particular, a careful attention should be paid to maintain unchanged technical parameters of reconstruction within patient.
- A standard diagnostic CT scan with (i.v.) contrast agent may, if appropriate, be carried out according to standard radiological methods **after** the low-dose CT without contrast agent and PET acquisition.

20.7 Appendix 13: Acquisition protocol for PET_D14

A FDG PET/CT (PET_D14) can be performed 14 day after CAR-T injection by sites with Siemens Vision camera using the fully-automated acquisition protocol for Siemens FlowMotion Multiparametric PET Suite Patlak.

A factory default Patlak workflow is present when a valid Patlak license is present and is called "WholebodyDynamicCBM Patlak." The Parametric PET feature is only available for FlowMotion acquisitions and is not available for step and shoot acquisitions.
In the automated workflow the ^{18}F FDG is injected on the bed at time t0, and the PET acquisition is started at the same time or just before. The acquisition chronicle is very similar to a whole-body dynamic FlowMotion acquisition; it just contains an extra single-bed position PET scan after the CT. The same CT is used for both PET acquisitions to perform attenuation and scatter corrections.

The defaults for the two PET acquisitions and reconstructions are outlined below.

1. Single-bed PET acquisition centered over patient's heart
   a. a. 6-minute acquisition duration
   b. Reconstruction
      i. Dynamic and used to generate the bolus part of the input function
         • 12 frames x 5s, 6 frames x 10s, 8 frames x 30s
      ii. decay correction is set to injection time

2. Whole-body dynamic PET acquisition
   a. 74-minute acquisition duration
   b. 18 total passes
      i. 8 passes x 3 min
      ii. 10 passes x 5 min
         • Best practice is to keep the duration of the passes the same regardless of patient height, so bed speed should be faster for taller patients
   c. 1st reconstruction is the online whole-body dynamic reconstructions of each pass
      i. decay correction is set to injection time t0
   d. 2nd reconstruction
      i. Dynamic reconstruction with the same axial FOV and reconstruction parameters as the single-bed cardiac region reconstruction 1.b above
      ii. These input region "tail" dynamic images will be merged with the bolus input region images from 1.b above, and this dynamic DICOM series will be stored in the database
      iii. Decay correction is set to injection time t0
   e. 3rd reconstruction
      i. Creates the image volume where SUVs can be calculated by summing the last six dynamic frames (50-80 minutes p.i.)
   f. 4th reconstruction
      i. Patlak reconstruction
         • Passes the last six sinograms to the Patlak reconstruction algorithm (50-80 minutes p.i., so t*=50 min)
         – Note: minimum number of sinograms for a Patlak reconstruction is 3 and the maximum is 6
         • Uses the aorta input function by default
         • The MR_{FDG} and DV_{FDG} Patlak Parametric Volumes will be stored in the database
         – If the tracer was not ^{18}F FDG or the patient's blood glucose was not entered, then the slope parametric volume will have units of ml/min/ml

20.8 Appendix 14: ASTCT CRS Grading

CRS parameter	Grade 1	Grade 2	Grade 3	Grade 4
Fever[a]	Temperature ≥ 38° C	Temperature ≥ 38° C	Temperature ≥ 38° C	Temperature ≥ 38° C
With				
Hypotension	None	Not requiring vasopressors	Requiring a vasopressor with or without vasopressin	Requiring multiple vasopressors (excluding vasopressin)
And/or[b]				
Hypoxia	None	Requiring low-flow nasal cannula[c] or blow-by	Requiring high-flow nasal cannula[c], facemask, nonrebreather mask, or Venturi mask	Requiring positive pressure (eg, CPAP, BiPAP, intubation and mechanical ventilation)

Organ toxicities associated with CRS may be graded according to CTCAE v5.0 but they do not influence CRS grading.

[a] Fever is defined as temperature ≥ 38°C not attributable to any other cause. In patients who have CRS then receive antipyretic or anticytokine therapy such as tocilizumab or corticosteroids, fever is no longer required to grade subsequent CRS severity. In this case, CRS grading is driven by hypotension and/or hypoxia.

[b] CRS grade is determined by the more severe event: hypotension or hypoxia not attributable to any other cause. For example, a patient with temperature of 39.5° C, hypotension requiring 1 vasopressor, and hypoxia requiring low-flow nasal cannula is classified as Grade 3 CRS.

[c] Low-flow nasal cannula is defined as oxygen delivered at ≤ 6 L/minute. Low flow also includes blow-by oxygen delivery, sometimes used in pediatrics. High-flow nasal cannula is defined as oxygen delivered at > 6 L/minute.

20.9 Appendix 15 - CRS Grading Scale (Excluding Neurologic Events) per Lee, 2014

Grade	Symptoms
**Grade 1**	Symptoms are not lifethreatening and require symptomatic treatment only (fever, nausea, fatigue, headache, myalgias, malaise)
**Grade 2**	Symptoms require and respond to moderate intervention:<br>• Oxygen requirement <40% FiO2 OR<br>• Hypotension responsive to i.v. fluids or low dose of one vasopressor OR<br>• Grade 2 organ toxicity*
**Grade 3**	Symptoms require and respond to aggressive intervention:<br>• Oxygen requirement ≥ 40% FiO2 OR<br>• Hypotension requiring high-dose** or multiple vasopressors OR<br>• Grade 3 organ toxicity* or grade 4 transaminitis
**Grade 4**	Life-threatening symptoms:<br>• Requirement for ventilator support OR<br>• Grade 4 organ toxicity* (excluding transaminitis)
**Grade 5**	Death

*Grades 2-4 refer to CTCAE v4.0 grading

**High-dose vasopressors (all doses are required for ≥3 hours):

Pressor	Dose
Norepinephrine monotherapy	≥20 μg/min
Dopamine monotherapy	≥10 μg/kg/min
Phenylephrine monotherapy	≥200 μg/min
Epinephrine monotherapy	≥10 μg/min
If on vasopressin	Vasopressin + norepinephrine equivalent of ≥10 μg/min*
If on combination vasopressors (not vasopressin)	Norepinephrine equivalent of ≥20 μg/min*

*VASST Trial vasopressor equivalent equation: norepinephrine equivalent dose = [norepinephrine (μg/min)] + [dopamine (μg/kg/min) ÷ 2] + [epinephrine (μg/min)] + [phenylephrine (μg/min) ÷10].

20.10 Appendix 16 – Management of CRS (based on Lee criteria)

CRS Grade[a]	Supportive care	Tocilizumab	Corticosteroids	Follow-up
**Grade 1**<br>Symptoms require symptomatic treatment only (eg, fever, nausea, fatigue, headache, myalgia, malaise)	- Supportive care per institutional standard of care<br>- Closely monitor neurologic status	N/A	N/A	Not improving after 24 hours:<br>- Tocilizumab: As per Grade 2 guidance (below)<br>Not improving after 3 days:<br>- Dexamethasone 10 mg IV x 1
**Grade 2**<br>- Symptoms require and respond to moderate intervention.<br>- Oxygen requirement less than 40% FiO_2 or hypotension responsive to fluids or low dose of one vasopressor or Grade 2 organ toxicity	- Continuous cardiac telemetry and pulse oximetry as indicated<br>- IV fluids bolus for hypotension with 0.5 to 1.0 L isotonic fluids<br>- Vasopressor support for hypotension not responsive to IV fluids<br>- Supplemental oxygen as indicated	- Tocilizumab: 8 mg/kg IV over 1 hour (not to exceed 800 mg)<br>- Repeat tocilizumab every 8 hours as needed if not responsive to IV fluids or increasing supplemental oxygen; maximum of 3 doses in a 24-hour period.<br>- Maximum total of 4 doses if no clinical improvement in the signs and symptoms of CRS	- Dexamethasone 10 mg IV once daily	Improving<br>- Manage as above<br>- Continue corticosteroids until the event is Grade 1 or less, then quickly taper as clinically appropriate<br>Not improving<br>- Manage as appropriate grade below
**Grade 3**<br>- Symptoms require and respond to aggressive intervention.<br>- Oxygen requirement greater than or equal to 40% FiO_2 or hypotension requiring high-dose or multiple vasopressors or Grade 3 organ toxicity or Grade 4 transaminitis.	- Management in monitored care or intensive care unit	- Per Grade 2	- Dexamethasone 10 mg IV three times a day	Improving<br>- Manage as appropriate grade above<br>- Continue corticosteroids until the event is Grade 1 or less, then quickly taper as clinically appropriate<br>Not improving<br>- Manage as Grade 4 (below)<br>- Contact Coordinating Investigators

**Grade 4**<br>- Life-threatening symptoms.<br>- Requirements for ventilator support or continuous veno-venous hemodialysis (CVVHD)<br>- Grade 4 organ toxicity (excluding transaminitis)	- Per Grade 3<br>- Mechanical ventilation and/or renal replacement therapy may be required	- Per Grade 2	- Methylprednisolone 1000 mg IV once daily x 3 days	Improving<br>- Manage as above appropriate grade above<br>- Continue corticosteroids until the event is Grade 1 or less, then taper as clinically appropriate<br>Not improving<br>- Consider 1 gram twice a day to three times a day of methylprednisolone or alternative therapy[b]<br>- Contact Coordinating Investigators

Abbreviations: IV, intravenous; CRS, cytokine release syndrome; FiO2, fraction of inspired oxygen; N/A, not applicable.

a. Modified Lee et al 2014

b. Initiation of alternative therapy should be discussed with the Coordinating Investigators and includes (but is not limited to): anakinra, siltuximab, ruxolitinib, cyclophosphamide, IVIG and ATG

20.11 Appendix 17 -- ASTCT Consensus Grading for Neurologic Events

ASCT Immune effector cell-associated neurotoxicity syndrome (ICANS) consensus grading

Neurotoxicity Domain	Grade 1	Grade 2	Grade 3	Grade 4
ICE score[a]	7 – 9	3 - 6	0 - 2	0 (patient is unarousable and unable to perform ICE)
Depressed level of consciousness[b]	Awakens spontaneously	Awakens to voice	Awakens only to tactile stimulus	Patient is unarousable or requires vigorous or repetitive tactile stimuli to arouse. Stupor or coma
Seizure	N/A	N/A	Any clinical seizure focal or generalized that resolved rapidly or nonconvulsive seizures on EEG that resolve with intervention	Life-threatening prolonged seizure (> 5 min); or Repetitive clinical or electrical seizures without return to baseline in between
Motor findings[c]	N/A	N/A	N/A	Deep focal motor weakness such as hemiparesis or paraparesis
Elevated ICP/cerebral edema	N/A	N/A	Focal/local edema on neuroimaging[d]	Diffuse cerebral edema on neuroimaging; decerebrate or decorticate posturing; or cranial nerve VI palsy; or papilledema; or Cushing's triad

Abbreviation: ASBMT, American Society for Blood and Marrow Transplantation; ICE, Immune Effector Cell-Associated Encephalopathy; ICP, Intracranial Pressure; N/A, not applicable

Notes: ICANS grade is determined by the most severe event (ICE score, level of consciousness, seizure, motor findings, raised ICP/cerebral edema) not attributable to any other cause; for example, a patient with an ICE score of 3 who has a generalized seizure is classified as Grade 3 ICANS.

[a] A patient with an ICE score of 0 may be classified as Grade 3 ICANS if awake with global aphasia, but a patient with an ICE score of 0 may be classified as Grade 4 ICANS if unarousable.

[b] Depressed level of consciousness should be attributable to no other cause (eg, no sedating medication).

[c] Tremors and myoclonus associated with immune effector cell therapies may be graded according to CTCAE v5.0, but they do not influence ICANS grading.

[d] Intracranial hemorrhage with or without associated edema is not considered a neurotoxicity feature and is excluded from ICANS grading. It may be graded according to CTCAE v5.0

Immune Effector Cell-Associated Encephalopathy (ICE) score

Task	Direction	Score
Orientation	Orientation to year, month, city, hospital	4
Naming	Ability to name 3 objects (eg, point to clock, pen, button)	3
Following simple commands	Ability to follow simple commands (eg, “Show me 2 fingers” or “Close your eyes and stick out your tongue”)	1
Writing	Ability to write a simple sentence	1
Attention	Ability to count backwards from 100 by 10’s	1

Abbreviation: ICE, Immune Effector Cell-Associated Encephalopathy.
Scoring: 10, no impairment; 7-9, Grade 1 ICANS; 3-6, Grade 2 ICANS; 0-2, Grade 3 ICANS; 0 due to patient unarousable and unable to perform ICE assessment, Grade 4 ICANS.

20.12 Appendix 18 - Management of Neurologic Events (based on CTCAE grading)

Neurologic Event (Grading assessment CTCAE version 5.0)	Supportive Care	Tocilizumab	Corticosteroids	Follow-up
**Grade 1**				
Examples include:<br>• Somnolence-mild drowsiness or sleepiness<br>• Confusion-mild disorientation<br>• Encephalopathy-mild limiting of ADLs<br>• Dysphasia-not imparing ability to communicate	• Supportive care per institutional standard of care<br>• Closely monitor neurologic status<br>• Consider prophylactic levetiracetam[a]	Concurrent CRS:<br>Per Grade 1 CRS guidance from Table in Appendix 16	• Dexamethasone 10 mg IV x 1	Not improving after 2 days<br>• Repeat dexamethasone 10 mg IV x 1<br>• Continue supportive care
**Grade 2**				
Examples include:<br>• Somnolence-moderate, limiting instrumental ADLs<br>• Confusion-moderate disorientation<br>• Encephalopathy-limiting instrumental ADLs<br>• Dysphasia-moderate impairing ability to communicate spontaneously<br>• Seizure(s)	• Continuous cardiac telemetry and pulse oximetry as indicated<br>• Closely monitor neurologic status with serial neuro exams to include fundoscopy and Glasgow Coma Score. Consider neurology consult.<br>• Perform brain imaging (eg. MRI), EEG, and lumbar puncture (with opening pressure) if no contraindications	Concurrent CRS:<br>• Tocilizumab: 8 mg/kg IV over 1 hour (not to exceed 800 mg)<br>• Repeat tocilizumab every 8 hours as needed if not responsive to IV fluids or increasing supplemental oxygen; maximum of 3 doses in a 24-hour period.<br>• Maximum total of 4 doses if no clinical improvement in the signs and symptoms of CRS	• Dexamethasone 10 mg IV four times a day	Improving<br>• Manage as above<br>• Continue corticosteroids until the event is Grade 1or less, then quickly taper as clinically appropriate<br>Not improving<br>• Manage as appropriate grade below<br>• Consider contacting Coordinating Investigators

**Neurologic Event (Grading assessment CTCAE version 5.0)**	**Supportive Care**	**Tocilizumab**	**Corticosteroids**	**Follow-up**
**Grade 3**				
Examples include:<br>• Somnolence-obtundation or stupor<br>• Confusion-severe disorientation<br>• Encephalopathy-limiting self-care ADLs<br>• Dysphasia-severe recepive or expressive characteristics, imparing ability to read, write, or communicate intelligibly	• Management in monitored care or intensive care unit	• Per Grade 2	• Methylprednisolone 1000 mg IV once daily	Improving<br>• Manage as appropriate grade above<br>• Continue corticosteroids until the event is Grade 1or less, then taper as clinically appropriate<br>Not improving<br>• Manage as Grade 4 (below)<br>• Contact Coordinating Investigators
**Grade 4**				
• Life-threatenning consequences<br>• Urgent intervention indicated<br>• Requirement for mechanical ventilation<br>• Consider cerebral edema (refer to Investigator's Brochure for the Management of Cerebral Edema)	• Per Grade 3<br>• Mechanica ventilation may be required	• Per Grade 2	Methylprednisolone 1000 mg IV twice a day	Improving<br>• Manage as appropriate grade above<br>• Continue corticosteroids until the event is Grade 1or less, then taper as clinically appropriate<br>Not improving<br>• Consider 1 gram of methylprednisolone three times a day or alternative thearpy[b]<br>• Contact Coordinating Investigators

Abbrevations: ADL (activities of daily life), CRS (cytokine release syndrome), CTCAE (Common Terminology Criteria for Adverse Events), EEG (electroencephalogram), MRI (magnetic resonance immaging)

[a] Prophylactic levetiracetam recommendation applies to all grades

[b] Initiation of alternative therapy should be discussed with the Coordinating Investigators and includes (but is not limited to): anakinra, siltuximab, ruxolitinib, cyclophosphamide, IVIG and ATG
