# Supplementary material for: Axicabtagene ciloleucel as second-line therapy in large B cell lymphoma ineligible for autologous stem cell transplantation: a phase 2 trial
Source: Nat Med. 2023 Sep 14;29(10):2593–601. doi: 10.1038/s41591-023-02572-5 (PMC10579056; doi:10.1038/s41591-023-02572-5)
Supplement: Supplementary file 2 — Reporting Summary [file 41591_2023_2572_MOESM2_ESM.pdf]

Corresponding author(s): Roch Houot

Last updated by author(s): Aug 22, 2023

Reporting Summary

Nature Portfolio wishes to improve the reproducibility of the work that we publish. This form provides structure for consistency and transparency in reporting. For further information on Nature Portfolio policies, see our Editorial Policies and the Editorial Policy Checklist.

Please do not complete any field with "not applicable" or n/a. Refer to the help text for what text to use if an item is not relevant to your study.
For final submission: please carefully check your responses for accuracy; you will not be able to make changes later.

Statistics

For all statistical analyses, confirm that the following items are present in the figure legend, table legend, main text, or Methods section.

n/a	Confirmed	
☐	☒	The exact sample size (*n*) for each experimental group/condition, given as a discrete number and unit of measurement
☒	☐	A statement on whether measurements were taken from distinct samples or whether the same sample was measured repeatedly
☐	☒	The statistical test(s) used AND whether they are one- or two-sided<br>*Only common tests should be described solely by name; describe more complex techniques in the Methods section.*
☐	☒	A description of all covariates tested
☐	☒	A description of any assumptions or corrections, such as tests of normality and adjustment for multiple comparisons
☐	☒	A full description of the statistical parameters including central tendency (e.g. means) or other basic estimates (e.g. regression coefficient) AND variation (e.g. standard deviation) or associated estimates of uncertainty (e.g. confidence intervals)
☐	☒	For null hypothesis testing, the test statistic (e.g. *F*, *t*, *r*) with confidence intervals, effect sizes, degrees of freedom and *P* value noted<br>*Give P values as exact values whenever suitable.*
☒	☐	For Bayesian analysis, information on the choice of priors and Markov chain Monte Carlo settings
☒	☐	For hierarchical and complex designs, identification of the appropriate level for tests and full reporting of outcomes
☒	☐	Estimates of effect sizes (e.g. Cohen's *d*, Pearson's *r*), indicating how they were calculated

Our web collection on statistics for biologists contains articles on many of the points above.

Software and code

Policy information about availability of computer code

Data collection	Electronic Data Capture (EDC) system from Ennov version 8.1 (Ennov, Paris, France).
Data analysis	EAST version 6.5 (Cytel Inc., Cambridge, MA), SAS version 9.3 or higher (SAS Institute Inc., Cary, NC), and AdClin version 3.2.2 or higher (AdClin, Paris, France).

For manuscripts utilizing custom algorithms or software that are central to the research but not yet described in published literature, software must be made available to editors and reviewers. We strongly encourage code deposition in a community repository (e.g. GitHub). See the Nature Portfolio guidelines for submitting code & software for further information.

Data

Policy information about availability of data

All manuscripts must include a data availability statement. This statement should provide the following information, where applicable:

- Accession codes, unique identifiers, or web links for publicly available datasets
- A description of any restrictions on data availability
- For clinical datasets or third party data, please ensure that the statement adheres to our policy

This trial is currently ongoing. Requests for access to aggregate data and supporting clinical documents will be reviewed and approved by an independent review panel on the basis of scientific merit. The datasets generated and/or analyzed during the current study are not publicly available due to proprietary considerations.

All data provided are anonymized to respect the privacy of patients who have participated in the trial, in line with applicable laws and regulations. Data requests pertaining to the manuscript may be made to the corresponding author (Roch Houot; roch.houot@chu-rennes.fr). Requests will be processed within 12 weeks.

Human research participants

Policy information about studies involving human research participants and Sex and Gender in Research.

Reporting on sex and gender	Among analyzed patients (n=62), 15 females and 47 males based on their biological sex were included in this study. We evaluated investigator-assessed complete metabolic response (CMR) rate at 3 months according to biological sex, and found no significant difference in CMR rate between males and females.
Population characteristics	This study population is composed of patients with relapsed/refractory aggressive B-cell non-Hodgkin lymphoma who are ineligible to autologous stem cell transplantation (ASCT). Among treated and analyzed patients (n=62), 29 patients were aged less than 70 years old and 33 patients were 70 years old or more. In addition, 32.3% of patients had a hematopoietic cell transplantation-specific comorbidity index (HCT-CI) score ≥3.
Recruitment	Patients were recruited by principal investigators (hematologists) in 18 sites across France among their patients or among patients referred from other hospitals from March 2021 to May 2022. To participate in this study and before any non-routine baseline or screening evaluation, the investigators at each study site ensured that each patient was fully informed of the study and had signed a written informed consent. The patient's eligibility was evaluated during the screening period prior to enrollment and leukapheresis. Eligible patients were aged 18 years or older with histologically confirmed aggressive B-cell non-Hodgkin lymphoma, diagnosed according to the 2016 World Health Organization classification criteria as either diffuse large B-cell lymphoma, high-grade B-cell lymphoma, or follicular lymphoma grade 3B. Disease had to be refractory to or had relapsed no more than 12 months after the completion of first-line chemoimmunotherapy containing an anti-CD20 monoclonal antibody and an anthracycline-containing regimen. Patients must also have been ineligible for ASCT based on the investigator's assessment and at least one of the following criteria: age ≥65 years, HCT-CI score ≥3 (as reported by investigators), or prior ASCT (as first-line consolidation). No bias emerging from recruitment is expected. The patients were not compensated for their participation in the study.
Ethics oversight	This study protocol was approved by French Ethics Committee Est I (Dijon) N°20.07.08.66206.

Note that full information on the approval of the study protocol must also be provided in the manuscript.

Field-specific reporting

Please select the one below that is the best fit for your research. If you are not sure, read the appropriate sections before making your selection.

☒ Life sciences ☐ Behavioural & social sciences ☐ Ecological, evolutionary & environmental sciences

For a reference copy of the document with all sections, see nature.com/documents/nr-reporting-summary-flat.pdf

Life sciences study design

All studies must disclose on these points even when the disclosure is negative.

Sample size	Sample size calculation was performed with EAST 6.5 using an exact single-stage phase II design. No interim analysis is planned. We hypothesized that axicabtagene ciloleucel (axi-cel) would yield a CMR at 3 months of 34% compared to 12% with a historical standard of care estimated from a retrospective, real-world cohort. On the basis of this assumption, the initial sample size was calculated to be 40 infused patients, with 96% power and a 0.05 alpha level (one-sided). To enable a balanced comparison of the efficacy of axi-cel in different age subgroups (<70 and ≥70 years), with a power of 85%, and considering potential dropouts, the required sample size was increased to 62 patients.
Data exclusions	No data were excluded from the analysis.
Replication	Not applicable because the ALYCANTE study is the first study to assess the efficacy and safety of axi-cel as second-line therapy in patients with relapsed/refractory aggressive B-cell non-Hodgkin lymphoma who are deemed ineligible for ASCT.
Randomization	No randomization was performed for this study, as this was a single-arm, phase II study. A subgroup analysis of CMR at 3 months was conducted for prespecified covariates, and a Forest plot was provided.
Blinding	No blinding was performed, as there was only one treatment group in this study.

Reporting for specific materials, systems and methods

We require information from authors about some types of materials, experimental systems and methods used in many studies. Here, indicate whether each material, system or method listed is relevant to your study. If you are not sure if a list item applies to your research, read the appropriate section before selecting a response.

Materials & experimental systems

n/a	Involved in the study
☒	☐ Antibodies
☒	☐ Eukaryotic cell lines
☒	☐ Palaeontology and archaeology
☒	☐ Animals and other organisms
☐	☒ Clinical data
☒	☐ Dual use research of concern

Methods

n/a	Involved in the study
☒	☐ ChIP-seq
☒	☐ Flow cytometry
☒	☐ MRI-based neuroimaging

Clinical data

Policy information about clinical studies

All manuscripts should comply with the ICMJE guidelines for publication of clinical research and a completed CONSORT checklist must be included with all submissions.

Clinical trial registration

NCT04531046.

Study protocol

The full study protocol is provided in the Supplementary Information.

Data collection

Data are recorded by on-site clinical research associates in the EDC system, based on medical files. Patients were recruited from March 2021 to May 2022 in 18 centers across France (Supplementary Table 1). The data cutoff date was 19 January 2023.

Outcomes

Primary outcome: Investigator-assessed CMR at 3 months from the axi-cel infusion (without additional anticancer therapy) (using the Lugano response criteria).
Secondary outcomes :
- CMR at 3 months from the axi-cel infusion (without additional anticancer therapy) determined by central imaging review (using the Lugano response criteria)
- Objective response rate (ORR) at 3 months: Percentage of CMR and partial metabolic response determined by investigator disease assessment as well as central imaging review
- Best investigator-assessed ORR and CMR
- Best ORR and best CMR as assessed by the central review panel
- Investigator-assessed CMR at 6 months
- Event-free survival from leukapheresis based on investigator disease assessment
- Progression-free survival from axi-cel infusion based on investigator disease assessment
- Duration of response
- Overall survival from axi-cel infusion
- Safety of axi-cel infusion

This checklist template is licensed under a Creative Commons Attribution 4.0 International License, which permits use, sharing, adaptation, distribution and reproduction in any medium or format, as long as you give appropriate credit to the original author(s) and the source, provide a link to the Creative Commons license, and indicate if changes were made. The images or other third party material in this article are included in the article's Creative Commons license, unless indicated otherwise in a credit line to the material. If material is not included in the article's Creative Commons license and your intended use is not permitted by statutory regulation or exceeds the permitted use, you will need to obtain permission directly from the copyright holder. To view a copy of this license, visit http://creativecommons.org/licenses/by/4.0/
